# Spatial, temporal, and demographic patterns in prevalence of smoking tobacco use and initiation among young people in 204 countries and territories, 1990–2019

**DOI:** 10.1016/S2468-2667(21)00102-X

**Published:** 2021-05-28

**Authors:** Marissa B Reitsma, Luisa S Flor, Erin C Mullany, Vin Gupta, Simon I Hay, Emmanuela Gakidou

**Affiliations:** a Institute for Health Metrics and Evaluation, Department of Health Metrics Sciences, University of Washington, Seattle, WA, USA

## Abstract

**Background:**

Universally, smoking cessation rates among established smokers are poor. Preventing young people from starting use of and becoming addicted to tobacco products remains a key strategy to end the tobacco epidemic. Previous country-specific studies have found that initiation of smoking tobacco use occurs predominantly among young people and have found mixed progress in reducing the prevalence of smoking tobacco use among young people. Current and comparable estimates for all countries are needed to inform targeted interventions and policies.

**Methods:**

We modelled two indicators: prevalence of current smoking tobacco use among young adults aged 15–24 years, and the age at which current smokers aged 20–54 years in 2019 began smoking regularly. We synthesised data from 3625 nationally representative surveys on prevalence of smoking and 254 on age at initiation. We used spatiotemporal Gaussian process regression to produce estimates of the prevalence of smoking and age of initiation by sex, for 204 countries and territories for each year between 1990 and 2019.

**Findings:**

Globally in 2019, an estimated 155 million (95% uncertainty interval 150–160) individuals aged 15–24 years were tobacco smokers, with a prevalence of 20·1% (19·4–20·8) among males and 4·95% (4·64–5·29) among females. We estimated that 82·6% (82·1–83·1) of current smokers initiated between ages 14 and 25 years, and that 18·5% (17·7–19·3) of smokers began smoking regularly by age 15 years. Although some countries have made substantial progress in reducing the prevalence of smoking tobacco use among young people, prevalence in 2019 still exceeds 20% among males aged 15–24 years in 120 countries and among females aged 15–24 years in 43 countries.

**Interpretation:**

The fact that most smokers start smoking regularly before age 20 years highlights the unique window of opportunity to target prevention efforts among young people and save millions of lives and avert health-care costs in the future. Countries can substantially improve the health of their populations by implementing and enforcing evidence-based tobacco control policies that prevent the next generation from initiating smoking.

**Funding:**

Bloomberg Philanthropies.

## Introduction

More than 1 billion people were estimated to be current tobacco smokers in 2019, hence, in the coming decades, the massive health and economic costs of the tobacco epidemic are likely to increase.^1^, ^2^ Large cohort studies have found that, at minimum, 50% of long-term tobacco smokers will die from causes directly linked to smoking, and that tobacco smokers have an average life expectancy that is 10 years shorter than that of people who have never smoked.^3^, ^4^ The broad effects of tobacco use and exposure, from reduced productivity to increasing inequality, make tobacco control a priority for countries working to meet the global progress targets of the UN Sustainable Development Goals (SDGs) and the WHO monitoring framework for non-communicable diseases.^2^, ^5^, ^6^, ^7^, ^8^

Among established tobacco smokers, cessation leads to a substantial reduction in risk, with the largest reductions among individuals who stop smoking before age 40 years.^9^ Although progress has been made in improving cessation treatments and services, achieving high rates of cessation remains elusive worldwide. Despite most smokers intending to quit, fewer than 10% do so successfully and only 32% of the global smoking population has access to evidence-based cessation support services.^2^, ^10^, ^11^ The health loss associated with tobacco use will remain substantial for years to come, particularly in low-income and middle-income countries, unless countries are able to substantially reduce the number of new smokers initiating each year. Strong efforts to prevent smoking initiation through effective policy and targeted interventions can interrupt this pipeline and yield massive long-term health and economic benefits.^1^, ^7^, ^12^

Previous studies have found that initiation of smoking tobacco use occurs predominantly among young people.^9^, ^13^, ^14^, ^15^ Behavioural and biological studies suggest that young people are particularly susceptible to addiction, and that most adult smokers regret starting smoking.^16^, ^17^, ^18^ Despite this abundant motivating evidence, a study of tobacco use among adolescents aged 13–15 years across 143 countries found variable progress in reducing prevalence in this age group.^19^

**Research in context**

**Evidence before this study**

Previous studies found that initiation of smoking tobacco use occurs predominantly among young people. An analysis of adolescents aged 13–15 years across 143 countries using Global Youth Tobacco Surveys found mixed progress in reducing prevalence of smoking tobacco use in this age group. Additional evidence suggests that adolescents and young people are more susceptible to addiction than older age groups and multi-country surveys consistently find that most adult smokers regret their decision to begin smoking.

**Added value of this study**

Using data from the Global Burden of Diseases, Injuries, and Risk Factors Study 2019, we estimated the prevalence of smoking tobacco use among young people (aged 15–24 years), by sex, for 204 countries and territories from 1990 to 2019. Our results are based on an analysis of more than 3000 surveys and provide timely estimates of the current situation and historical trends using a modelling framework that was consistently applied across countries. Our estimates of prevalence among people aged 15–24 years complement estimates of prevalence among the aged 13–15 years group, which might underestimate the magnitude of prevalence because initiation continues to occur after age 15 years. Additionally, we estimated the mean age of initiation of smoking tobacco use and a complete distribution of initiation ages among current smokers for all countries. Using this distribution, we identified the crucial age window during which young people transition to becoming regular smokers in each country, providing locally relevant estimates of a novel indicator that can be used to target interventions and policies to prevent initiation.

**Implications of all the available evidence**

Across all countries and both sexes, we found clear and consistent evidence that young people transition to become regular smokers in a narrow age window spanning adolescence and young adulthood. With 89·1% of eventual smokers initiating smoking by age 25 years, ensuring that young people remain smoke-free through their mid-twenties should result in substantial reductions in the prevalence of smoking in the next generation. Considered alongside behavioural and biological evidence on nicotine addiction and the documented feeling of regret among most adult smokers, our findings refute tobacco industry claims that smoking is an informed choice. To protect susceptible young people from lifelong nicotine addiction, governments and societies must intensify their efforts at prevention and imminently adopt, implement, and enforce strong tobacco control policies and interventions that target prevention of initiation of smoking tobacco use among young people.

We aimed to examine patterns of smoking tobacco use among young people, by sex, for 204 countries and territories. We analysed prevalence of current smoking among people aged 15–24 years. We also estimated the mean age of initiation and characterised the full distribution of age at initiation among current smokers aged 20–54 years in 2019 to identify the age window in which people are becoming addicted to nicotine across the globe, and link results to opportunities for intervention that can change the course of the tobacco epidemic for the next generation.

## Methods

### Overview and definitions

We analysed prevalence data for current smoking tobacco use from the Global Burden of Diseases, Injuries, and Risk Factors Study (GBD) 2019 among young people (ie, aged 15–24 years), by sex, for 204 countries and territories from 1990 to 2019.^1^, ^20^ We defined current smoking tobacco use as use of any type of smoked tobacco product on a daily or occasional basis. Smoked tobacco products include manufactured cigarettes, hand-rolled cigarettes, cigars, cigarillos, pipes, shisha, and regional products such as bidis and kreteks. Methods for estimating the prevalence of current smoking tobacco use have been published separately.^1^ Additionally, we estimated the mean age of smoking initiation and characterised the complete distribution of ages when current smokers aged 20–54 years began smoking regularly. This study adheres to the Guidelines for Accurate and Transparent Health Estimates Reporting.^21^

### Data sources

We obtained data on prevalence of smoking tobacco use and age at initiation from a comprehensive database of nationally representative surveys on tobacco use compiled by GBD. This database is updated annually and the source list is available online through the Global Health Data Exchange. We collected data from surveys that reported smoking tobacco use among individuals aged 10 years and older collected between 1980 and 2019. Although we report data for individuals aged 15 years and older and from 1990 onwards, we included this additional age group and decade to inform time trends and age patterns of the model. Further details on inclusion criteria, survey case definitions, adjustment for non-standard case definitions, and other data processing are included in appendix 1 (pp 9–12) and have been published previously.^1^

For the estimation of prevalence of smoking tobacco use, we used 3625 nationally representative surveys with data on prevalence of smoking, of which 3085 had data on smoking among individuals aged 15–19 years and 2217 had data on smoking among individuals aged 20–24 years; most data sources covered both age groups. For the estimation of mean age at initiation, we used 254 nationally representative surveys with individual-level data on age of initiation (appendix 2 pp 3–8). All data included estimates of uncertainty, which we incorporated into the modelled estimates.

### Modelling

We used spatiotemporal Gaussian process regression to produce a time series, by location, age, and sex for mean age at initiation.^1^ This modelling approach is widely used for time-varying risk factors in GBD.^20^ This approach synthesises all available data sources, incorporates uncertainty of data into modelled estimates, and allows patterns observed in countries with ample data to inform estimates in similar countries that have little or no data. Reported 95% uncertainty intervals (UIs) are based on a sample of 1000 draws from the model. The lower bound is the 2·5th percentile and the upper bound is the 97·5th percentile of the distribution of draws.

After modelling the mean age at initiation, we estimated the complete distribution of age at initiation for each location, 5-year age group, and by sex. We use paired data on mean age at initiation and SD of age at initiation from the same survey, 5-year age group, and sex to fit a linear regression that predicts log(SD) from log(mean age at initiation). We used this regression to predict an estimated SD for each of the 1000 draws of mean age at initiation, by location, year, age, and sex. Together, we used these two parameters to characterise the distribution of age at initiation.

We used an ensemble approach to construct a probability density function for age at initiation, instead of using a single distribution, to allow for more flexibility to capture the shape of the empirical distribution. We constructed the ensemble distribution as a linear combination of eleven distributions (beta, exponential, gamma, inverse gamma, mirror gamma, gumbel, mirror gumbel, log-logistic, lognormal, normal, and Weibull), which were combined using weights that were selected to minimise the Kolmogorov-Smirnov test statistic.^20^ We optimised the values of the weights using individual-level data by region, age group, and sex. Optimising over region, age group, and sex prevents overfitting to a single dataset, while allowing the shape of the distribution to vary by demographic group. Using the estimated mean, SD, and ensemble weights, we constructed probability density functions and cumulative distribution functions of age at initiation for every location, year, age, and by sex at the 1000 draw level.

### Statistical analysis

We report prevalence of smoking tobacco use and number of smokers aged 15–24 years by location and sex in the year 2019, and analysed changes in these indicators between 1990 and 2019, all with their respective 95% UIs. Furthermore, we report mean age of initiation among current smokers aged 20–54 years in 2019, by sex and location, and the 10th and 90th percentiles of the distribution of initiation age. We chose the 20–54 years age range to avoid introducing downward bias in estimates of age at initiation, particularly in countries where there is a heavier tail on the distribution of initiation age (appendix 1 p 10). Changes between 1990 and 2019 were considered significant if the 95% UI of the change excluded zero.

We did all analyses using the R (version 3.6).

### Role of the funding source

The funder of the study had no role in study design, data collection, data analysis, data interpretation, or writing of the report.

## Results

**Figure 1:**
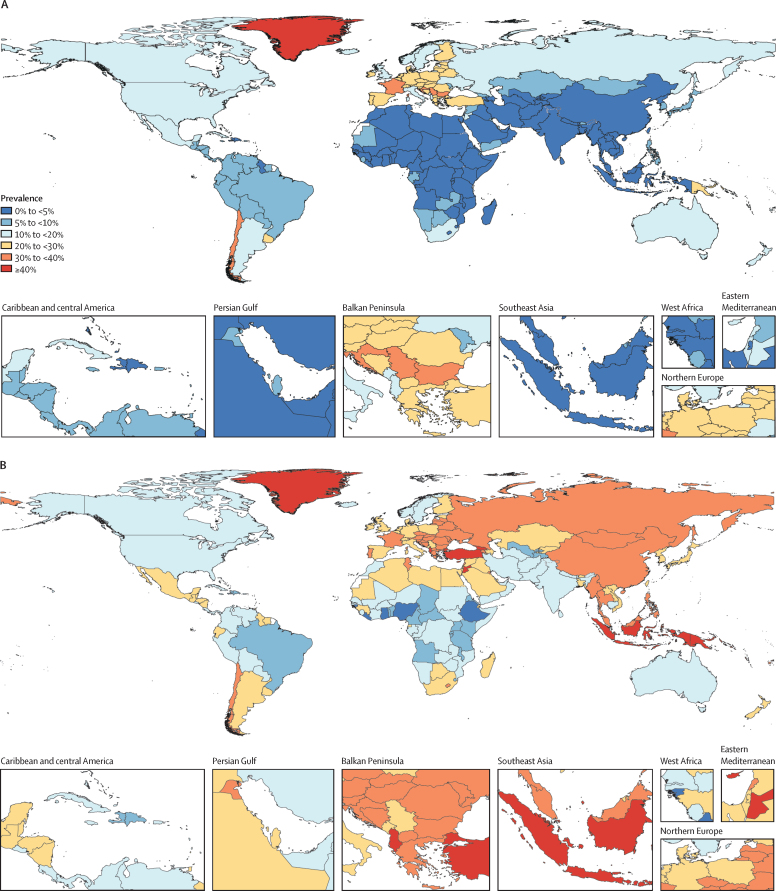
Prevalence of smoking tobacco use among females (A) and males (B) aged 15–24 years, in 2019

In 2019, an estimated 155 million (95% UI 150–160) individuals aged 15–24 years globally were tobacco smokers. Prevalence of smoking tobacco use in this age group was 20·1% (19·4–20·8) among males and 4·95% (4·64–5·29) among females (appendix 2 pp 9–17). Prevalence of smoking tobacco use exceeded 20% among males aged 15–24 years in 120 countries and exceeded 20% among females aged 15–24 years in 43 countries (figure 1; appendix 2 pp 9–17). In 12 locations in 2019, more than 33% of people aged 15–24 years were current smokers. Five of these locations were islands in the Pacific (Kiribati, Federated States of Micronesia, Papua New Guinea, Cook Islands, and Nauru), four were in Europe (Bulgaria, Croatia, Latvia, and France), and the last three locations were Chile, Turkey, and Greenland. Among countries with a population of more than 1 million people, prevalence of smoking tobacco use among males was highest in Timor-Leste (52·8% [48·8–57·0]), Papua New Guinea (46·7% [41·3–52·0]), and Turkey (45·6% [42·2–48·9]), and among females prevalence was highest in Bulgaria (39·7% [32·5–47·1]), Chile (39·2% [29·8–49·2]), and Croatia (34·9% [28·8–41·0]; appendix 2 pp 9–17). Among all 204 countries and territories included in this analysis, Federated States of Micronesia, Kiribati, Timor-Leste, Greenland, and Papua New Guinea had the highest prevalence among males aged 15–24 years, and Greenland, Nauru, Bulgaria, Chile, and Federated States of Micronesia had the highest prevalence among females aged 15–24 years (appendix 2 pp 9–17). The countries with the largest numbers of current tobacco smokers aged 15–24 years in 2019 were China (26·5 million [23·8–29·4]), India (19·8 million [16·7–23·2]), and Indonesia (9·91 million [9·18–10·7]; appendix 2 pp 18–26). The ten countries with the largest number of tobacco smokers aged 15–24 years in 2019 accounted for 55·9% of total tobacco smokers in this age group (table).

**Table:** Current smoking tobacco use in 2019 and initiation patterns in the ten countries with the largest populations of smokers aged 15–24 years in 2019, by sex

	**Number of current tobacco smokers aged 15–24 years (thousands)**		**Prevalence of smoking among individuals aged 15–24 years (%)**		**Mean age at initiation (years)**		**Age at initiation (10th–90th percentile; years)**	
	Females	Males	Females	Males	Females	Males	Females	Males
China	1610 (994–2630)	25 000 (22 300–27 500)	2·17% (1·34–3·55)	30·1% (27·0–33·2)	24·9 (23·8–26·0)	20·5 (20·1–20·9)	16–35	15–27
India	2520 (1440–3970)	17 300 (14 400–20 400)	1·98% (1·13–3·13)	12·6% (10·5–14·9)	22·1 (20·9–23·4)	20·6 (20·1–21·1)	14–35	14–29
Indonesia	491 (290–778)	9420 (8720–10 200)	2·24% (1·32–3·54)	41·7% (38·6–45·1)	20·8 (19·9–21·6)	18·3 (17·9–18·7)	14–30	13–24
USA	2800 (2180–3470)	4070 (3610–4630)	13·3% (10·3–16·4)	18·4% (16·3–21·0)	17·5 (17·1–17·9)	17·3 (17·0–17·6)	13–23	13–22
Turkey	1420 (1170–1700)	3120 (2890–3350)	22·0% (18·2–26·4)	45·6% (42·2–48·9)	20·0 (19·4–20·6)	17·3 (17·0–17·7)	14–27	13–22
Philippines	687 (482–956)	3540 (3170–3920)	6·70% (4·70–9·32)	33·1% (29·7–36·7)	22·9 (21·9–23·8)	17·9 (17·6–18·3)	15–35	13–24
Mexico	1100 (785–1450)	2910 (2590–3230)	10·1% (7·25–13·4)	27·0% (24·0–29·9)	18·9 (18·4–19·4)	16·9 (16·6–17·3)	13–26	12–22
Bangladesh	178 (93·9–302)	3720 (3230–4280)	1·14% (0·599–1·92)	26·1% (22·7–30·0)	26·3 (24·8–28·0)	18·3 (17·9–18·7)	14–40	13–25
Russia	1040 (815–1290)	2340 (2120–2550)	14·7% (11·5–18·2)	31·6% (28·7–34·4)	18·6 (18·2–19·0)	16·6 (16·3–16·9)	13–25	12–21
Pakistan	408 (227–701)	2800 (2270–3360)	1·83% (1·01–3·14)	11·9% (9·66–14·3)	22·1 (20·8–23·3)	20·0 (19·5–20·5)	14–35	14–29

Count data, prevalence data, and mean age at initiation are given to three significant figures. Data in parentheses are 95% uncertainty intervals. Prevalence and number of smokers are reported for individuals aged 15–24 years in 2019. Mean age of initiation window is reported for current smokers aged 20–54 years in 2019. The age window of initiation is defined as the range between the 10th and 90th percentiles of age at initiation.

**Figure 2:**
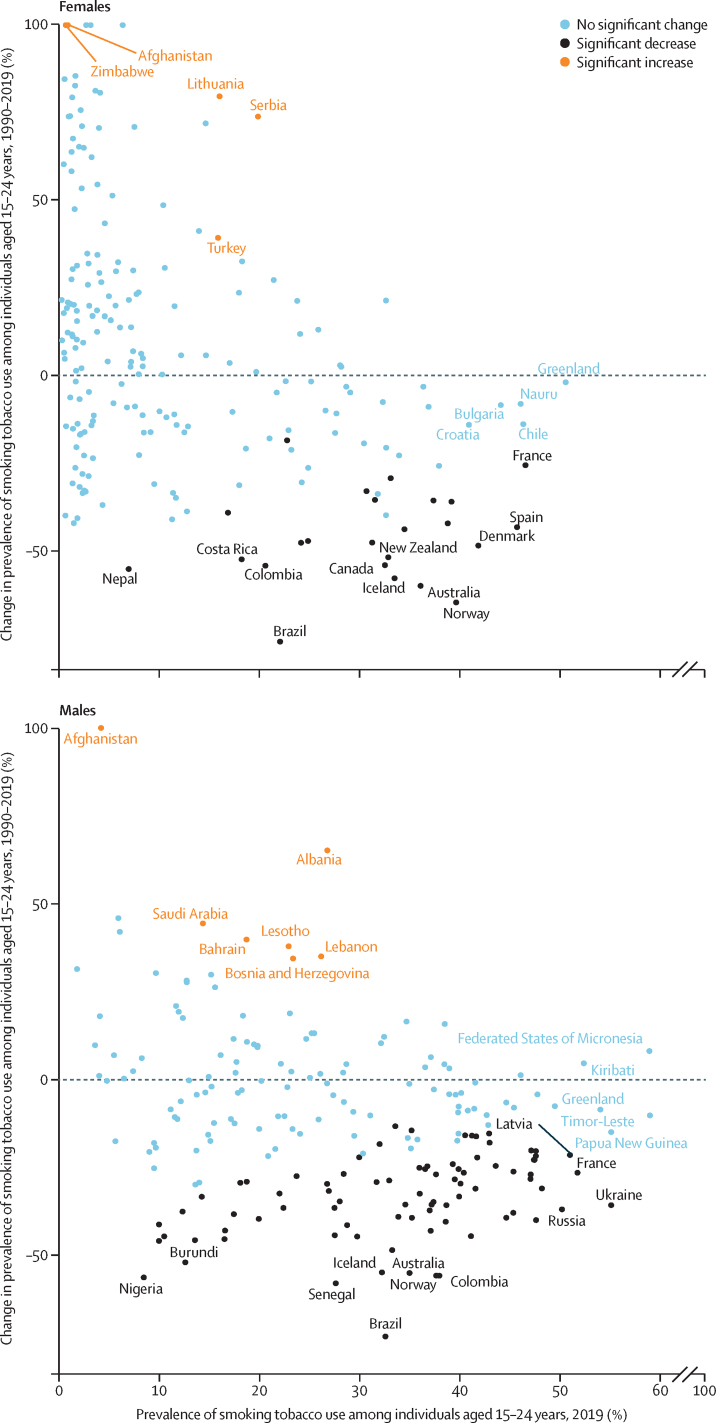
Prevalence of smoking tobacco use in 2019 *vs* percentage change in prevalence between 1990 and 2019, among individuals aged 15–24 years, by sex Percentage change exceeded 100% for five countries (Albania, El Salvador, Afghanistan, Zambia, and Zimbabwe) for females and one country (Afghanistan) for males. These values were capped at 100% for visualisation purposes. To avoid visual clutter, only locations with significant increases, significant and large decreases, and high prevalence are labelled.

Prevalence of smoking tobacco use decreased significantly between 1990 and 2019 among both males (32·9% [95% UI 29·9 to 35·9] decrease) and females (37·6% [32·2 to 42·7] decrease). Progress in reducing the prevalence of smoking varied substantially across countries (figure 2; appendix 2 pp 27–35). Of 204 countries and territories included in our analysis, only 81 (40%) had significant decreases in prevalence among individuals aged 15–24 years since 1990. The largest absolute decreases in the prevalence of smoking in this age group between 1990 and 2019 were observed in Norway (from 38·9% [36·0 to 41·7] in 1990 to 15·3% [12·8 to 18·3] in 2019; absolute change of –23·5 percentage points [–19·5 to –27·3]), Australia (from 35·8% [34·6 to 36·9] in 1990 to 15·1% [12·3 to 18·8] in 2019; absolute change of –20·6 percentage points [–16·9 to –27·3]), and Brazil (from 27·5% [25·2 to 30·0] in 1990 to 7·01% [5·85 to 8·31] in 2019; absolute change of –20·5 percentage points [–17·9 to –23·1]). 12 countries (Albania, Bosnia and Herzegovina, Serbia, Afghanistan, Lebanon, Bahrain, Lesotho, Saudi Arabia, El Salvador, Zambia, Antigua and Barbuda, and Uzbekistan) had significant increases in the prevalence of smoking in this age group between 1990 and 2019. The remaining 111 countries had no significant changes in prevalence over the past 30 years (appendix 2 pp 27–35).

More countries had significant reductions in the prevalence of smoking tobacco use between 1990 and 2019 in males (n=83) than had significant reductions in the prevalence of smoking in females (n=24; figure 2; appendix 2 pp 27–35). Significant reductions in the prevalence of smoking were observed in countries across all initial levels of prevalence in 1990. The effect of population growth was relatively greater than reductions in prevalence in smoking tobacco use in 72 countries for males and 41 countries for females, resulting in significant increases in the number of smokers aged 15–24 years between 1990 to 2019 (appendix 2 pp 36–44). India, Egypt, and Indonesia had the largest absolute increases in the number of male smokers aged 15–24 years (4·67 million [95% UI 1·31–8·12] in India, 1·24 million [0·864–1·59] in Egypt, and 1·22 million [0·206–2·22] in Indonesia). The largest absolute increases in the number of females smokers aged 15–24 years were seen in Turkey (468 000 [127–840]), Jordan (116 000 [66·2–174]), and Zambia (110 000 [51·1–183]; (appendix 2 pp 45–53).

**Figure 3:**
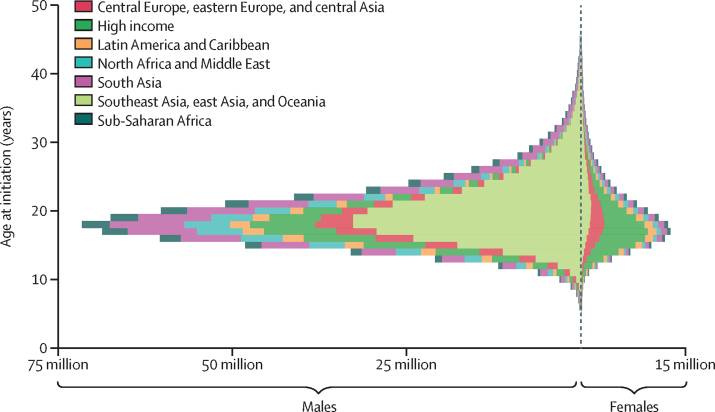
Individual-level distribution of age at initiation among current tobacco smokers aged 20–54 years in 2019, by super-region and sex The sizes of the bars indicate the total number of current tobacco smokers initiating at a given age. Data for males are displayed to the left of the vertical dashed line and data for females are displayed to the right.

**Figure 4:**
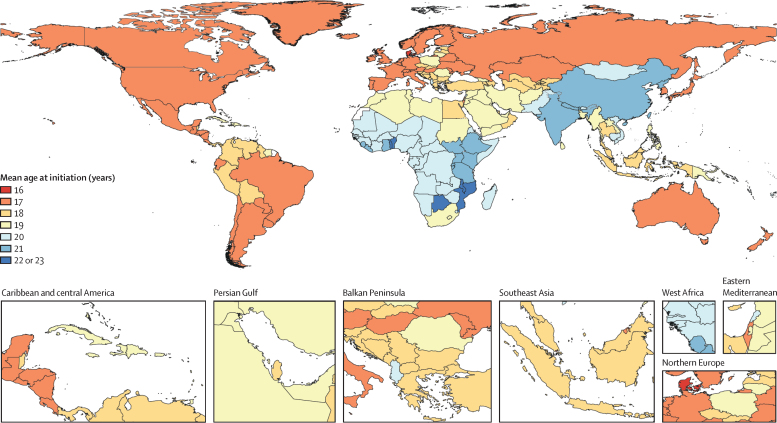
Mean age at initiation of regular smoking of tobacco among current smokers aged 20–54 years in 2019, both sexes combined

Globally, 89·1% (95% UI 88·5–89·7) of current smokers aged 20–54 years in 2019 began smoking tobacco regularly by age 25 years, and 82·6% (82·1–83·1) began smoking in the 12-year window between ages 14 and 25 years (figure 3). Across all countries, the mean age at initiation of regular smoking of tobacco was 19·2 years (19·1–19·4). An estimated 18·5% (17·7–19·3) of current smokers aged 20–54 years in 2019 began smoking regularly by age 15 years, while 65·5% (64·3–66·5) began smoking by age 20 years. Across countries, the mean proportion of current smokers aged 20–54 years in 2019 who initiated smoking by age 21 years was 76·6% (59·0–97·5). The youngest mean ages at initiation were observed in Europe and the Americas. The oldest mean ages at initiation were seen in east and south Asia and sub-Saharan Africa (figure 3). Across countries and for both sexes combined, the mean age at initiation ranged from 16·4 years (16·2–16·7) in Denmark to 22·5 years (22·0–23·1) in Togo (figure 4; appendix 2 pp 54–62). Among the ten countries with the largest numbers of smokers aged 15–24 years in 2019, the lowest mean ages at initiation were observed in Russia (16·6 years [16·3–16·9]) for males and in the USA (17·5 years [17·1–17·9]) for females. The highest mean ages at initiation were observed in India (20·6 years [20·1–21·1]) for males and Bangladesh (26·3 years [24·8–28·0]) for females (table).

Males began smoking earlier than females in 194 (95%) of 204 countries, although the magnitude of between-sex difference varies by region. The difference in mean age of initiation between males and females is as small as 0·3 years (95% UI 0·2–0·4) in countries in the high-income super-region and as large as 3·8 years (3·1–4·6) in Southeast Asia, east Asia, and Oceania super-region (appendix 2 pp 52–62). Countries with the largest difference between males and females in the mean age at initiation also had the largest difference in prevalence of smoking tobacco use between males and females (appendix 2 pp 27–35, 63). Notably, in countries where prevalence of smoking tobacco use among people aged 15–24 years has decreased significantly, the mean age of initiation has remained constant over time. Of 81 locations that had significant decreases in prevalence among this age group, mean age of initiation did not change significantly in 76 locations and mean age of initiation increased by less than 1 year in the remaining five locations.

## Discussion

Despite decades of accumulated evidence of the harmful lifelong consequences of smoking tobacco use, the prevalence of smoking among young people remains high in many parts of the world. In 2019, global prevalence of smoking tobacco use among males aged 15–24 years was 20·1% (95% UI 19·4–20·8) and among females was 4·95% (4·64–5·29). 65·5% (64·3–66·5) of all current smokers aged 20–54 years in 2019 began smoking by age 20 years, with 82·6% (82·1–83·1) of all current smokers initiating during a 12-year window between the ages of 14 and 25 years. This age range is the crucial window during which individuals develop nicotine addiction and transition to becoming established smokers. Tobacco will remain a problem for generations to come if initiation of smoking tobacco use in this age window is not substantially reduced.

Our findings are important for two reasons: first, smoking tobacco use is probably the only risk factor among the top five global risk factors for mortality for which such an opportunity for intervention during a short window of time in a person's life exists. Unlike other risk factors, such as obesity, diet, and hypertension, if an individual does not become a regular smoker by age 25 years, then they are unlikely to become a smoker. This finding alone highlights the unique opportunity for interventions that target prevention of smoking initiation to individuals aged 25 years and younger. Second, a comprehensive package of evidence-based tobacco control interventions exists that have been shown to be effective among young people.^22^, ^23^, ^24^, ^25^, ^26^, ^27^, ^28^ Hence, again, the risk factor of smoking tobacco use is unique compared with other risk factors, such as obesity, for which effective interventions have not yet been identified or implemented at a population level. Implementation and enforcement of strong tobacco control policies have led to substantial progress in protecting young people and reducing the number of young smokers in some countries. For example, Brazil had the largest decrease in prevalence of smoking tobacco use among individuals aged 15–24 years, with a decrease in prevalence of 74·5% (95% UI 69·0–78·9) from 27·5% (25·2–30·0) in 1990 to 7·01% (5·85–8·31) in 2019.^29^ The fact that mean age of initiation has remained consistent across time in countries that have experienced significant decreases in prevalence is encouraging evidence that interventions prevent smoking altogether, as opposed to only delaying the age at which people start smoking. Yet, persistently high prevalence of smoking tobacco use in young people in most countries, along with the increased use of e-cigarettes and vaping products that risk reversing progress,^30^ highlight an urgent need to invest more strongly in tobacco control to protect young people from nicotine addiction.

Effective interventions can be broadly grouped into two categories, demand reduction policies and supply-side restrictions.^24^ Strong implementation and enforcement of interventions are crucial to success in all settings.^24^, ^25^ The MPOWER framework was introduced by WHO in 2008 to guide implementation of effective demand reduction interventions, following the WHO Framework Convention on Tobacco Control entering into force and becoming international binding law in 2005.^2^ The framework recommends that countries enact comprehensive smoke-free policies; use health warning labels and anti-tobacco mass media campaigns; ban tobacco advertising, promotion, and sponsorship; and decrease the affordability of tobacco products to reduce demand and prevalence of smoking tobacco use. The recommended interventions have been shown to reduce initiation of smoking among young people, and these strategies are even more effective when implemented jointly.^22^, ^23^, ^24^, ^25^, ^26^, ^27^, ^28^ Despite notable progress in some countries, interference from the tobacco industry and waning political commitment have resulted in a large and persistent gap between knowledge and action on global tobacco control.^2^

Among tobacco control strategies, decreasing the affordability of tobacco products is one of the most effective mechanisms for reducing overall prevalence of smoking tobacco use, especially among young people who are particularly responsive to price changes.^23^, ^31^ Over the past decade, globally, 50% of countries decreased the affordability of tobacco products.^2^ Over the next decade, as countries look to accelerate progress towards to the SDGs, expanding and strengthening fiscal policy to reduce affordability of tobacco products is an essential component to preventing young people from starting to smoke.

As the tobacco industry innovates new ways to market their products, including leveraging social media to reach young people through marketing campaigns and so-called influencers, tobacco control strategies must also evolve.^30^, ^32^, ^33^ In the digital age, bans on advertising, promotion, and sponsorship recommended by MPOWER must extend to internet-based media, but as of 2018 only 25% of countries have comprehensively banned all forms of direct and indirect advertising.^2^ Closing these loopholes and partnering with key social media stakeholders to enforce such bans is crucial to protecting young people from the harmful effects of tobacco. Social media also presents opportunities for anti-tobacco media campaigns to connect with young people.^32^

Beyond the core demand-reduction interventions outlined in MPOWER, flavour bans and minimum age of purchase polices have gained momentum as tools for reducing initiation of smoking tobacco use in young people. Flavours have an important role in attracting young people to tobacco, an issue that has been exacerbated and exposed by the emergence of vaping.^34^, ^35^ Banning all flavours, including menthol, across all nicotine-containing products, including smoked tobacco products, smokeless tobacco products, and e-cigarette, vaping, and heated tobacco products is a promising approach to reducing demand among young people.^36^, ^37^ Despite the clear link to initiation among young people, in 2020, fewer than 60 countries have enacted flavour bans that range from partial bans of only some flavours to complete bans of all flavours. Increasing the minimum age of purchase of tobacco products is another policy option. Globally, the highest observed minimum age of purchase at the national level is 21 years, with six countries using this benchmark (the USA in 2019, Uganda in 2015, Honduras in 2011, Sri Lanka in 2006, Samoa in 2008, and Kuwait in 1995). Most countries have their legal purchase age set at either 16 or 18 years. We found most current smokers in 2019 began smoking regularly by age 21 years. Across countries, the average proportion of smokers who initiated by age 21 years is 76·6% (95% UI 59·0–97·5). Little empirical evidence exists on the effectiveness of increasing the minimum age of purchase on preventing smoking tobacco use among young people, with some subnational studies suggesting that increasing the age to 21 years leads to reductions in the prevalence of smoking among individuals younger than 21 years.^38^, ^39^ More data are needed to assess the effect of youth access laws.

The findings of this study should be interpreted alongside its limitations. First, smoking status was self-reported in all surveys used and so data are subject to response bias. As a result, in countries where social acceptability of smoking is low among any or all demographic groups, smoking might be under-reported. Second, age at initiation of smoking is subject to recall bias, particularly among older respondents. Third, because age at initiation of smoking was estimated among smokers, in demographic groups with low prevalence of smoking data are based on a small number of respondents, potentially resulting in large variance or outliers. Fourth, we report aggregated estimates on age at initiation of smoking for current smokers aged 20–54 years in 2019. Although we found that age at initiation was relatively stable over time, these aggregated estimates reflect initiation across four decades, and cohort effects might affect the results. Fifth, estimates in countries without data reflect regional data plus additional uncertainty. Sociocultural or policy differences might limit the appropriateness of these regional approximations in some cases. Additional data collection can improve these estimates in the future. Sixth, most surveys on smoking among young people are done in schools, which might not be representative of the general population. Seventh, surveys vary in their question wording and case definition. Although we adjusted for differences in case definition, these adjustments introduce additional uncertainty and might not fully remove biases. Finally, we focused only on smoked tobacco and did not include smokeless tobacco, e-cigarettes, vaping products, or heated tobacco products. Previous studies have suggested that non-cigarette tobacco products comprise an increasingly important portion of tobacco use among young people.^19^ As more data become available on these products in the coming years, analysing how they relate to initiation and tobacco use among young people will be crucial.

Initiation of smoking occurred predominantly before age 25 years in all 204 countries and territories included in our study. Protecting young people from becoming addicted to tobacco products is the only path to a tobacco-free generation. The evidence-based tobacco control strategies necessary to curb smoking initiation rates need to be adopted, implemented, and enforced with renewed vigour. In 2019, an estimated 155 million people aged 15–24 years—approximately 13% of this population globally—smoke tobacco regularly. To end the tobacco epidemic, countries must aggressively implement evidence-based strategies to prevent initiation and interrupt the steady stream of new smokers.

## Data sharing

To download the data used in these analyses, please visit the Global Health Data Exchange GBD 2019 website.

## Declaration of interests

We declare no competing interests.

## References

[1] GBD 2019 Tobacco Collaborators (2021). Spatial, temporal, and demographic patterns in tobacco smoking prevalence and attributable disease: a systematic analysis of 204 countries and territories from the Global Burden of Disease Study 2019. Lancet.

[2] WHO (2019). WHO report on the global tobacco epidemic 2019: offer help to quit tobacco use. http://www.who.int/tobacco/global_report/en/.

[3] Doll R, Peto R, Boreham J, Sutherland I (2004). Mortality in relation to smoking: 50 years' observations on male British doctors. BMJ.

[4] Jha P, Ramasundarahettige C, Landsman V (2013). 21st-century hazards of smoking and benefits of cessation in the United States. N Engl J Med.

[5] WHO Regional Office for South-East Asia (2017). Tobacco control for sustainable development. https://apps.who.int/iris/handle/10665/255509.

[6] WHO (2017). Tobacco control for sustainable development. https://apps.who.int/iris/handle/10665/255509.

[7] Goodchild M, Nargis N, Tursan d'Espaignet E (2018). Global economic cost of smoking-attributable diseases. Tob Control.

[8] Bilano V, Gilmour S, Moffiet T (2015). Global trends and projections for tobacco use, 1990-2025: an analysis of smoking indicators from the WHO Comprehensive Information Systems for Tobacco Control. Lancet.

[9] National Center for Chronic Disease Prevention and Health Promotion (US) Office on Smoking and Health (2014). The health consequences of smoking—50 years of progress: a report of the surgeon general.

[10] Babb S, Malarcher A, Schauer G, Asman K, Jamal A (2017). Quitting smoking among adults - United States, 2000–2015. MMWR Morb Mortal Wkly Rep.

[11] Hajek P, Phillips-Waller A, Przulj D (2019). A randomized trial of e-cigarettes versus nicotine-replacement therapy. N Engl J Med.

[12] Jha P, Peto R (2014). Global effects of smoking, of quitting, and of taxing tobacco. N Engl J Med.

[13] Freedman KS, Nelson NM, Feldman LL (2012). Smoking initiation among young adults in the United States and Canada, 1998–2010: a systematic review. Prev Chronic Dis.

[14] Marcon A, Pesce G, Calciano L (2018). Trends in smoking initiation in Europe over 40 years: a retrospective cohort study. PLoS One.

[15] National Center for Chronic Disease Prevention and Health Promotion (US) Office on Smoking and Health, National Center for Chronic Disease Prevention and Health Promotion (US) Office on Smoking and Health (2012). The epidemiology of tobacco use among young people in the United States and worldwide. Preventing tobacco use among youth and young adults: a report of the surgeon general.

[16] Kessler DA, Natanblut SL, Wilkenfeld JP (1997). Nicotine addiction: a pediatric disease. J Pediatr.

[17] Fong GT, Hammond D, Laux FL (2004). The near-universal experience of regret among smokers in four countries: findings from the International Tobacco Control Policy Evaluation Survey. Nicotine Tob Res.

[18] Sansone N, Fong GT, Lee WB (2013). Comparing the experience of regret and its predictors among smokers in four Asian countries: findings from the ITC surveys in Thailand, South Korea, Malaysia, and China. Nicotine Tob Res.

[19] Ma C, Xi B, Li Z (2021). Prevalence and trends in tobacco use among adolescents aged 13–15 years in 143 countries, 1999–2018: findings from the Global Youth Tobacco Surveys. Lancet Child Adolesc Health.

[20] GBD 2019 Risk factors Collaborators (2020). Global burden of 87 risk factors in 204 countries and territories, 1990–2019: a systematic analysis for the Global Burden of Disease Study 2019. Lancet.

[21] Stevens GA, Alkema L, Black RE (2016). Guidelines for Accurate and Transparent Health Estimates Reporting: the GATHER statement. Lancet.

[22] Henriksen L (2012). Comprehensive tobacco marketing restrictions: promotion, packaging, price and place. Tob Control.

[23] Ross H, Chaloupka FJ (2003). The effect of cigarette prices on youth smoking. Health Econ.

[24] Lantz PM, Jacobson PD, Warner KE (2000). Investing in youth tobacco control: a review of smoking prevention and control strategies. Tob Control.

[25] Levy DT, Friend KB (2002). Strategies for reducing youth access to tobacco: a framework for understanding empirical findings on youth access policies. Drugs Educ Prev Policy.

[26] Francis DB, Mason N, Ross JC, Noar SM (2019). Impact of tobacco-pack pictorial warnings on youth and young adults: a systematic review of experimental studies. Tob Induc Dis.

[27] Robertson L, Cameron C, McGee R, Marsh L, Hoek J (2016). Point-of-sale tobacco promotion and youth smoking: a meta-analysis. Tob Control.

[28] Song AV, Dutra LM, Neilands TB, Glantz SA (2015). Association of smoke-free laws with lower percentages of new and current smokers among adolescents and young adults: an 11-year longitudinal study. JAMA Pediatr.

[29] Portes LH, Machado CV, Turci SRB, Figueiredo VC, Cavalcante TM, Silva VLDCE (2018). Tobacco control policies in Brazil: a 30-year assessment. Cien Saude Colet.

[30] de Andrade M, Hastings G, Angus K (2013). Promotion of electronic cigarettes: tobacco marketing reinvented?. BMJ.

[31] Ding A (2003). Youth are more sensitive to price changes in cigarettes than adults. Yale J Biol Med.

[32] Freeman B (2012). New media and tobacco control. Tob Control.

[33] O'Brien EK, Hoffman L, Navarro MA, Ganz O (2020). Social media use by leading US e-cigarette, cigarette, smokeless tobacco, cigar and hookah brands. Tob Control.

[34] Carpenter CM, Wayne GF, Pauly JL, Koh HK, Connolly GN (2005). New cigarette brands with flavors that appeal to youth: tobacco marketing strategies. Health Aff (Millwood).

[35] Villanti AC, Johnson AL, Glasser AM (2019). Association of flavored tobacco use with tobacco initiation and subsequent use among US youth and adults, 2013–2015. JAMA Netw Open.

[36] WHO Study Group on Tobacco Product Regulation (TobReg) (2016). Advisory note: banning menthol in tobacco products. http://www.who.int/tobacco/publications/prod_regulation/menthol-advisory-note/en/.

[37] Courtemanche CJ, Palmer MK, Pesko MF (2017). Influence of the flavored cigarette ban on adolescent tobacco use. Am J Prev Med.

[38] Friedman AS, Wu RJ (2020). Do local tobacco-21 laws reduce smoking among 18 to 20 year-olds?. Nicotine Tob Res.

[39] Kessel Schneider S, Buka SL, Dash K, Winickoff JP, O'Donnell L (2016). Community reductions in youth smoking after raising the minimum tobacco sales age to 21. Tob Control.

