# Supplementary material for: Spatial, temporal, and demographic patterns in prevalence of smoking tobacco use and initiation among young people in 204 countries and territories, 1990–2019
Source: Lancet Public Health. 2021 May 28;6(7):e472–81. doi: 10.1016/S2468-2667(21)00102-X (PMC8251503; doi:10.1016/S2468-2667(21)00102-X)
Supplement: Supplementary appendix 1 [file mmc1.pdf]

THE LANCET
Public Health

Supplementary appendix 1

This appendix formed part of the original submission and has been peer reviewed. We post it as supplied by the authors.

Supplement to: Reitsma MB, Flor LS, Mullany EC, Gupta V, Hay SI, Gakidou E. Spatial, temporal, and demographic patterns in prevalence of smoking tobacco use and initiation among young people in 204 countries and territories, 1990–2019. *Lancet Public Health* 2021; published online May 27. http://dx.doi.org/10.1016/S2468-2667(21)00102-X.

Supplementary Methods

Spatial, temporal, and demographic patterns in prevalence of smoking tobacco use and initiation among young people in 204 countries and territories, 1990-2019

Marissa B Reitsma, ScB[1], Luisa S Flor, PhD[1], Erin Mullany BS[1], Vin Gupta, MD, MSc[1], Simon I Hay DPhil, DSc[1], Emmanuela Gakidou, PhD[1*]

[1]Institute for Health Metrics and Evaluation, Department of Health Metrics Sciences, University of Washington, Seattle, WA, USA

Table of Contents

Geographies Estimated 3

Case Definitions 9

Demographics 10

Inclusion Criteria 11

Data extraction 12

Adjustment for Non-Standard Case Definitions 13

Modeling Smoking Prevalence and Age of Initiation 15

References 16

GATHER Checklist 17

Geographies Estimated

Using results from the Global Burden of Diseases, Injuries, and Risk Factors Study 2019 (GBD 2019), we report estimates for 204 countries and territories (Table SM1). These countries and territories are nested in 21 aggregate regions and seven aggregate super-regions. Standard regions and super-regions in the GBD are defined based on a combination of epidemiologic patterns and spatial distance.

Table SM1. Geographic Hierarchy

Location	Super-Region	Region
**Armenia**	Central Europe, Eastern Europe, and Central Asia	Central Asia
**Azerbaijan**	Central Europe, Eastern Europe, and Central Asia	Central Asia
**Georgia**	Central Europe, Eastern Europe, and Central Asia	Central Asia
**Kazakhstan**	Central Europe, Eastern Europe, and Central Asia	Central Asia
**Kyrgyzstan**	Central Europe, Eastern Europe, and Central Asia	Central Asia
**Mongolia**	Central Europe, Eastern Europe, and Central Asia	Central Asia
**Tajikistan**	Central Europe, Eastern Europe, and Central Asia	Central Asia
**Turkmenistan**	Central Europe, Eastern Europe, and Central Asia	Central Asia
**Uzbekistan**	Central Europe, Eastern Europe, and Central Asia	Central Asia
**Albania**	Central Europe, Eastern Europe, and Central Asia	Central Europe
**Bosnia and Herzegovina**	Central Europe, Eastern Europe, and Central Asia	Central Europe
**Bulgaria**	Central Europe, Eastern Europe, and Central Asia	Central Europe
**Croatia**	Central Europe, Eastern Europe, and Central Asia	Central Europe
**Czechia**	Central Europe, Eastern Europe, and Central Asia	Central Europe
**Hungary**	Central Europe, Eastern Europe, and Central Asia	Central Europe
**Montenegro**	Central Europe, Eastern Europe, and Central Asia	Central Europe
**North Macedonia**	Central Europe, Eastern Europe, and Central Asia	Central Europe
**Poland**	Central Europe, Eastern Europe, and Central Asia	Central Europe
**Romania**	Central Europe, Eastern Europe, and Central Asia	Central Europe
**Serbia**	Central Europe, Eastern Europe, and Central Asia	Central Europe
**Slovakia**	Central Europe, Eastern Europe, and Central Asia	Central Europe
**Slovenia**	Central Europe, Eastern Europe, and Central Asia	Central Europe
**Belarus**	Central Europe, Eastern Europe, and Central Asia	Eastern Europe
**Estonia**	Central Europe, Eastern Europe, and Central Asia	Eastern Europe
**Latvia**	Central Europe, Eastern Europe, and Central Asia	Eastern Europe
**Lithuania**	Central Europe, Eastern Europe, and Central Asia	Eastern Europe
**Republic of Moldova**	Central Europe, Eastern Europe, and Central Asia	Eastern Europe

**Russia**	Central Europe, Eastern Europe, and Central Asia	Eastern Europe
**Ukraine**	Central Europe, Eastern Europe, and Central Asia	Eastern Europe
**Australia**	High-income	Australasia
**New Zealand**	High-income	Australasia
**Brunei Darussalam**	High-income	High-income Asia Pacific
**Japan**	High-income	High-income Asia Pacific
**Republic of Korea**	High-income	High-income Asia Pacific
**Singapore**	High-income	High-income Asia Pacific
**Canada**	High-income	High-income North America
**Greenland**	High-income	High-income North America
**United States of America**	High-income	High-income North America
**Argentina**	High-income	Southern Latin America
**Chile**	High-income	Southern Latin America
**Uruguay**	High-income	Southern Latin America
**Andorra**	High-income	Western Europe
**Austria**	High-income	Western Europe
**Belgium**	High-income	Western Europe
**Cyprus**	High-income	Western Europe
**Denmark**	High-income	Western Europe
**Finland**	High-income	Western Europe
**France**	High-income	Western Europe
**Germany**	High-income	Western Europe
**Greece**	High-income	Western Europe
**Iceland**	High-income	Western Europe
**Ireland**	High-income	Western Europe
**Israel**	High-income	Western Europe
**Italy**	High-income	Western Europe
**Luxembourg**	High-income	Western Europe
**Malta**	High-income	Western Europe
**Monaco**	High-income	Western Europe
**Netherlands**	High-income	Western Europe
**Norway**	High-income	Western Europe
**Portugal**	High-income	Western Europe
**San Marino**	High-income	Western Europe
**Spain**	High-income	Western Europe
**Sweden**	High-income	Western Europe
**Switzerland**	High-income	Western Europe
**United Kingdom**	High-income	Western Europe
**Bolivia (Plurinational State of)**	Latin America and Caribbean	Andean Latin America

**Ecuador**	Latin America and Caribbean	Andean Latin America
**Peru**	Latin America and Caribbean	Andean Latin America
**Antigua and Barbuda**	Latin America and Caribbean	Caribbean
**Bahamas**	Latin America and Caribbean	Caribbean
**Barbados**	Latin America and Caribbean	Caribbean
**Belize**	Latin America and Caribbean	Caribbean
**Bermuda**	Latin America and Caribbean	Caribbean
**Cuba**	Latin America and Caribbean	Caribbean
**Dominica**	Latin America and Caribbean	Caribbean
**Dominican Republic**	Latin America and Caribbean	Caribbean
**Grenada**	Latin America and Caribbean	Caribbean
**Guyana**	Latin America and Caribbean	Caribbean
**Haiti**	Latin America and Caribbean	Caribbean
**Jamaica**	Latin America and Caribbean	Caribbean
**Puerto Rico**	Latin America and Caribbean	Caribbean
**Saint Kitts and Nevis**	Latin America and Caribbean	Caribbean
**Saint Lucia**	Latin America and Caribbean	Caribbean
**Saint Vincent and the Grenadines**	Latin America and Caribbean	Caribbean
**Suriname**	Latin America and Caribbean	Caribbean
**Trinidad and Tobago**	Latin America and Caribbean	Caribbean
**United States Virgin Islands**	Latin America and Caribbean	Caribbean
**Colombia**	Latin America and Caribbean	Central Latin America
**Costa Rica**	Latin America and Caribbean	Central Latin America
**El Salvador**	Latin America and Caribbean	Central Latin America
**Guatemala**	Latin America and Caribbean	Central Latin America
**Honduras**	Latin America and Caribbean	Central Latin America
**Mexico**	Latin America and Caribbean	Central Latin America
**Nicaragua**	Latin America and Caribbean	Central Latin America
**Panama**	Latin America and Caribbean	Central Latin America
**Venezuela (Bolivarian Republic of)**	Latin America and Caribbean	Central Latin America
**Brazil**	Latin America and Caribbean	Tropical Latin America
**Paraguay**	Latin America and Caribbean	Tropical Latin America
**Afghanistan**	North Africa and Middle East	North Africa and Middle East
**Algeria**	North Africa and Middle East	North Africa and Middle East
**Bahrain**	North Africa and Middle East	North Africa and Middle East
**Egypt**	North Africa and Middle East	North Africa and Middle East
**Iran (Islamic Republic of)**	North Africa and Middle East	North Africa and Middle East
**Iraq**	North Africa and Middle East	North Africa and Middle East

**Jordan**	North Africa and Middle East	North Africa and Middle East
**Kuwait**	North Africa and Middle East	North Africa and Middle East
**Lebanon**	North Africa and Middle East	North Africa and Middle East
**Libya**	North Africa and Middle East	North Africa and Middle East
**Morocco**	North Africa and Middle East	North Africa and Middle East
**Oman**	North Africa and Middle East	North Africa and Middle East
**Palestine**	North Africa and Middle East	North Africa and Middle East
**Qatar**	North Africa and Middle East	North Africa and Middle East
**Saudi Arabia**	North Africa and Middle East	North Africa and Middle East
**Sudan**	North Africa and Middle East	North Africa and Middle East
**Syrian Arab Republic**	North Africa and Middle East	North Africa and Middle East
**Tunisia**	North Africa and Middle East	North Africa and Middle East
**Turkey**	North Africa and Middle East	North Africa and Middle East
**United Arab Emirates**	North Africa and Middle East	North Africa and Middle East
**Yemen**	North Africa and Middle East	North Africa and Middle East
**Bangladesh**	South Asia	South Asia
**Bhutan**	South Asia	South Asia
**India**	South Asia	South Asia
**Nepal**	South Asia	South Asia
**Pakistan**	South Asia	South Asia
**China**	Southeast Asia, East Asia, and Oceania	East Asia
**Democratic People's Republic of Korea**	Southeast Asia, East Asia, and Oceania	East Asia
**Taiwan (Province of China)**	Southeast Asia, East Asia, and Oceania	East Asia
**American Samoa**	Southeast Asia, East Asia, and Oceania	Oceania
**Cook Islands**	Southeast Asia, East Asia, and Oceania	Oceania
**Fiji**	Southeast Asia, East Asia, and Oceania	Oceania
**Guam**	Southeast Asia, East Asia, and Oceania	Oceania
**Kiribati**	Southeast Asia, East Asia, and Oceania	Oceania
**Marshall Islands**	Southeast Asia, East Asia, and Oceania	Oceania
**Micronesia (Federated States of)**	Southeast Asia, East Asia, and Oceania	Oceania
**Nauru**	Southeast Asia, East Asia, and Oceania	Oceania
**Niue**	Southeast Asia, East Asia, and Oceania	Oceania
**Northern Mariana Islands**	Southeast Asia, East Asia, and Oceania	Oceania
**Palau**	Southeast Asia, East Asia, and Oceania	Oceania
**Papua New Guinea**	Southeast Asia, East Asia, and Oceania	Oceania
**Samoa**	Southeast Asia, East Asia, and Oceania	Oceania
**Solomon Islands**	Southeast Asia, East Asia, and Oceania	Oceania
**Tokelau**	Southeast Asia, East Asia, and Oceania	Oceania

**Tonga**	Southeast Asia, East Asia, and Oceania	Oceania
**Tuvalu**	Southeast Asia, East Asia, and Oceania	Oceania
**Vanuatu**	Southeast Asia, East Asia, and Oceania	Oceania
**Cambodia**	Southeast Asia, East Asia, and Oceania	Southeast Asia
**Indonesia**	Southeast Asia, East Asia, and Oceania	Southeast Asia
**Lao People's Democratic Republic**	Southeast Asia, East Asia, and Oceania	Southeast Asia
**Malaysia**	Southeast Asia, East Asia, and Oceania	Southeast Asia
**Maldives**	Southeast Asia, East Asia, and Oceania	Southeast Asia
**Mauritius**	Southeast Asia, East Asia, and Oceania	Southeast Asia
**Myanmar**	Southeast Asia, East Asia, and Oceania	Southeast Asia
**Philippines**	Southeast Asia, East Asia, and Oceania	Southeast Asia
**Seychelles**	Southeast Asia, East Asia, and Oceania	Southeast Asia
**Sri Lanka**	Southeast Asia, East Asia, and Oceania	Southeast Asia
**Thailand**	Southeast Asia, East Asia, and Oceania	Southeast Asia
**Timor-Leste**	Southeast Asia, East Asia, and Oceania	Southeast Asia
**Viet Nam**	Southeast Asia, East Asia, and Oceania	Southeast Asia
**Angola**	Sub-Saharan Africa	Central Sub-Saharan Africa
**Central African Republic**	Sub-Saharan Africa	Central Sub-Saharan Africa
**Congo**	Sub-Saharan Africa	Central Sub-Saharan Africa
**Democratic Republic of the Congo**	Sub-Saharan Africa	Central Sub-Saharan Africa
**Equatorial Guinea**	Sub-Saharan Africa	Central Sub-Saharan Africa
**Gabon**	Sub-Saharan Africa	Central Sub-Saharan Africa
**Burundi**	Sub-Saharan Africa	Eastern Sub-Saharan Africa
**Comoros**	Sub-Saharan Africa	Eastern Sub-Saharan Africa
**Djibouti**	Sub-Saharan Africa	Eastern Sub-Saharan Africa
**Eritrea**	Sub-Saharan Africa	Eastern Sub-Saharan Africa
**Ethiopia**	Sub-Saharan Africa	Eastern Sub-Saharan Africa
**Kenya**	Sub-Saharan Africa	Eastern Sub-Saharan Africa
**Madagascar**	Sub-Saharan Africa	Eastern Sub-Saharan Africa
**Malawi**	Sub-Saharan Africa	Eastern Sub-Saharan Africa
**Mozambique**	Sub-Saharan Africa	Eastern Sub-Saharan Africa
**Rwanda**	Sub-Saharan Africa	Eastern Sub-Saharan Africa
**Somalia**	Sub-Saharan Africa	Eastern Sub-Saharan Africa
**South Sudan**	Sub-Saharan Africa	Eastern Sub-Saharan Africa
**Uganda**	Sub-Saharan Africa	Eastern Sub-Saharan Africa
**United Republic of Tanzania**	Sub-Saharan Africa	Eastern Sub-Saharan Africa
**Zambia**	Sub-Saharan Africa	Eastern Sub-Saharan Africa

**Botswana**	Sub-Saharan Africa	Southern Sub-Saharan Africa
**Eswatini**	Sub-Saharan Africa	Southern Sub-Saharan Africa
**Lesotho**	Sub-Saharan Africa	Southern Sub-Saharan Africa
**Namibia**	Sub-Saharan Africa	Southern Sub-Saharan Africa
**South Africa**	Sub-Saharan Africa	Southern Sub-Saharan Africa
**Zimbabwe**	Sub-Saharan Africa	Southern Sub-Saharan Africa
**Benin**	Sub-Saharan Africa	Western Sub-Saharan Africa
**Burkina Faso**	Sub-Saharan Africa	Western Sub-Saharan Africa
**Cabo Verde**	Sub-Saharan Africa	Western Sub-Saharan Africa
**Cameroon**	Sub-Saharan Africa	Western Sub-Saharan Africa
**Chad**	Sub-Saharan Africa	Western Sub-Saharan Africa
**Côte d'Ivoire**	Sub-Saharan Africa	Western Sub-Saharan Africa
**Gambia**	Sub-Saharan Africa	Western Sub-Saharan Africa
**Ghana**	Sub-Saharan Africa	Western Sub-Saharan Africa
**Guinea**	Sub-Saharan Africa	Western Sub-Saharan Africa
**Guinea-Bissau**	Sub-Saharan Africa	Western Sub-Saharan Africa
**Liberia**	Sub-Saharan Africa	Western Sub-Saharan Africa
**Mali**	Sub-Saharan Africa	Western Sub-Saharan Africa
**Mauritania**	Sub-Saharan Africa	Western Sub-Saharan Africa
**Niger**	Sub-Saharan Africa	Western Sub-Saharan Africa
**Nigeria**	Sub-Saharan Africa	Western Sub-Saharan Africa
**São Tomé and Príncipe**	Sub-Saharan Africa	Western Sub-Saharan Africa
**Senegal**	Sub-Saharan Africa	Western Sub-Saharan Africa
**Sierra Leone**	Sub-Saharan Africa	Western Sub-Saharan Africa
**Togo**	Sub-Saharan Africa	Western Sub-Saharan Africa

Case Definitions

Current smoking is defined as use of any type of smoked tobacco product on a daily or occasional basis. Smoked tobacco products include manufactured cigarettes, hand-rolled cigarettes, cigars, cigarillos, pipes, shisha, and regional products such as bidis and kreteks.

Age of initiation is defined as the age at which an individual began using smoked tobacco products. Our preferred definition was the age at which an individual began regularly using smoked tobacco products. In cases when this definition was unavailable, we used the age at which an individual first used smoked tobacco products. Due to recall bias and heaping, we did not detect a significant difference between these two definitions when analyzing matched data by location, age, sex, and time period.

Demographics

For the 204 countries and territories included in analysis, we analyzed estimates by sex and five-year age group from 1990 to 2019.

Age Group for Prevalence Estimates: We report estimates of smoking prevalence among young people between the ages of 15 and 24. To do so, we produce population-weighted aggregate estimates of smoking prevalence among ages 15-19 and ages 20-24.

Age Group for Initiation Age Estimates: We report estimates of initiation age among current smokers between the ages of 20 and 54. Estimates for each five-year age group are aggregated weighting by the population of current smokers in each age group. We chose this wider age range, compared to the age range of 15 to 24 used for prevalence estimates, to avoid introducing downward bias in estimates of initiation age, particularly in countries where there is a heavier tail on the distribution of initiation age. Although this wider age range includes events that happened over a four-decade time period, bias due to cohort effects is likely limited due to the consistency of the indicator across time, assessed among countries for which data spanning multiple time periods are available.

Inclusion Criteria

We systematically reviewed surveys with information on tobacco use available in the Global Health Data Exchange (GHDx, http://ghdx.healthdata.org/). Surveys were extracted if they met our inclusion criteria, which were the following:

- Included indicators of tobacco use that met our standard case definitions, or alternatively, indicators that could be reliably adjusted to meet our standard case definitions (see "adjusting for non-standard case-definitions" section)
- Representative of the general population of one of the 204 countries and territories included in our analysis
    - Excludes surveys conducted exclusively among sub-populations (including those with a specific disease, racial/ethnic minorities, pregnant women, etc.)
    - One exception is that school-based surveys were included for individuals in primary or secondary school. Most surveys covering tobacco use among youth rely on school-based data collection, resulting in a paucity of population-representative data. As a result, we chose to include school-based surveys in order to have broad data coverage for youth ages 10-17.
- Data collection occurred between January 1, 1980 and December 31, 2019. We include data collected between 1980-1989 to inform time trends, even though we do not report on this time period.
- Tobacco use was self-reported by the individual, not by a proxy respondent
- Respondents were ages 10 and above
- For initiation age estimates, we include surveys with individual level data available, allowing us to characterize the full distribution of initiation ages in the population

Data extraction

We extracted primary data from individual-level microdata and survey report tabulations (tabulated estimates include for the current smoking prevalence indicator). For microdata, we extracted relevant demographic information, including age, sex, location, and year, as well as survey metadata, including survey weights, primary sampling units, and strata. This information allowed us to tabulate individual-level data in the standard GBD five-year age-sex groups and produce accurate estimates of uncertainty. When survey design indicators are not reported in the microdata, we assume a conservative design effect of 2.25. For survey report tabulations, we extracted data at the most granular age-sex group provided. For the age of initiation indicator, we also computed the sample standard deviation, which is utilized in constructing the distribution of initiation age.

Adjustment for Non-Standard Case Definitions

Smoking Prevalence: Our GBD smoking case definition is current use of any smoked tobacco product. All other data points were adjusted to be consistent with these definitions. Some sources contained information on more than one case definition and these sources were used to develop the adjustment coefficients to transform alternative case definitions to the GBD standard case definitions. The adjustment coefficient was the beta value derived from the following linear regression model:

$$p_{gs,k} = \beta p_{alt,k} + \epsilon_k$$

where $p_{gs,k}$ is the prevalence based on the standard case definition in survey k and $p_{alt,k}$ is the prevalence based on an alternative case definition that is also reported in survey k.

Since smoking patterns vary by age, we fit separate models for adults over age 20, teens ages 15-19, and youth ages 10-14. Models for all combinations of case definitions were fit. Models with adjusted R-squared values > 0.8 were then ranked in order of their R-squared value. For surveys with multiple non-standard case definitions, the single coefficient from the most highly ranked model was used for adjustment. In order to capture differences in smoking pattern by geography, weights were assigned to data based on a hierarchy of geographic regions. When considering an adjustment for a country from region r, surveys used to fit the model from the same geographic region were given two times the weight of surveys outside that region, and a maximum of 200 of the geographically closest points were included in each model. We tested applying a similar weight based on time period, but found that it did not significantly change estimated coefficients. At minimum, 20 sources were used to fit crosswalk coefficients. Due to data limitations, we could only fit global models for youth adjustments.

We propagated uncertainty at the survey (k) level from the crosswalk using the following equation:

$$PE_k = \sigma_\epsilon^2 + X_k^2 var(\hat{\beta})$$

where PE_k is the crosswalk prediction error that is added to the sampling variance of the data point, σ_ϵ^2 is the variance of the error, X_k^2 is the squared value of the data being adjusted, and $var(\hat{\beta})$ is the variance of the adjustment coefficient.

Adjustment coefficients are reported in the supplemental appendix reporting smoking attributable burden estimates from GBD 2019.[1]

Age of Initiation: Although we included data from two case definitions (age of regular smoking initiation and age of first smoked tobacco use), due to recall bias and heaping among respondent of our target age group (current smokers ages 20 to 54) we did not detect significant differences between these two definitions. As a result, we do not adjust for bias in our alternative case definition (age of first smoked tobacco use). We note this as a potential limitation in our study.

Modeling Smoking Prevalence and Age of Initiation

Further details on modeling smoking prevalence are published in full elsewhere.[1] Briefly, we utilize spatiotemporal Gaussian process regression to model both current smoking prevalence and mean age of initiation. This modeling approach is widely used for time-varying risk factors in the Global Burden of Disease Study.[2] It synthesizes all available data sources, incorporates uncertainty of data into modeled estimates, and allows patterns observed in countries with ample data to inform estimates in similar countries that lack data. For mean age of initiation, we also characterize the full distribution of initiation age among current smokers. We did not want to impose an overly restrictive functional form on the distribution of initiation age. As a result, we employed an ensemble method to combine multiple probability density functions in order to better match empirical distributions observed in survey data. An example of an ensemble distribution is shown in Supplemental Figure SM2.

Figure SM1. Example Ensemble Distribution

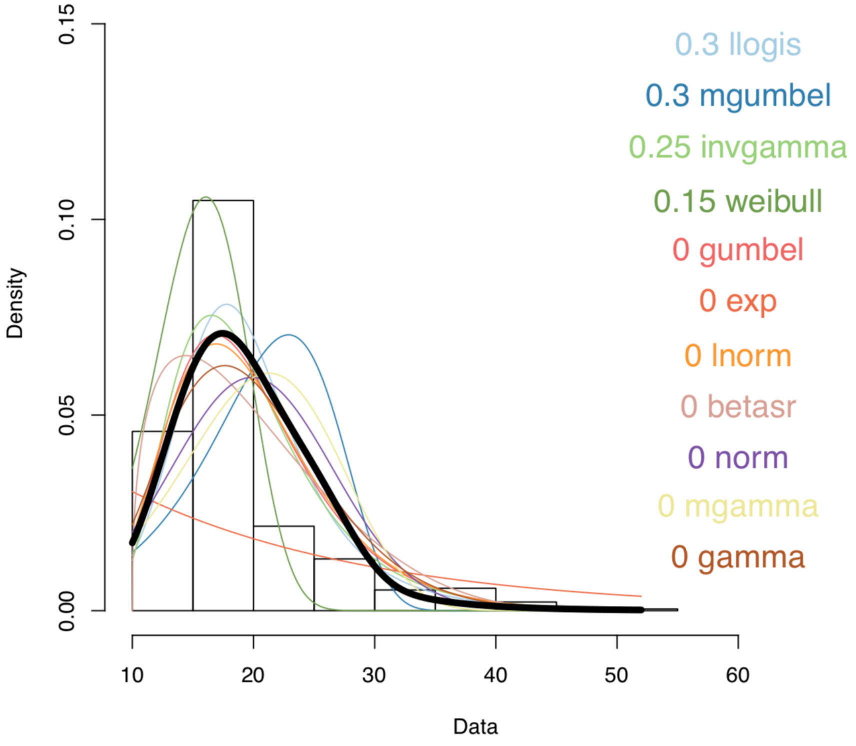

References

1 GBD 2019 Tobacco Collaborators. Spatial, temporal, and demographic patterns in tobacco smoking prevalence and attributable disease: a systematic analysis of 204 countries and territories from the Global Burden of Disease Study 2019. *Lancet* Accepted.

2 Murray CJL, Aravkin AY, Zheng P, *et al.* Global burden of 87 risk factors in 204 countries and territories, 1990–2019: a systematic analysis for the Global Burden of Disease Study 2019. *The Lancet* 2020; **396**: 1223–49.

GATHER Checklist

GATHER
Guidelines for Accurate and Transparent Health Estimates Reporting

Item #	Checklist item	Reported on page #
**Objectives and funding**		
**1**	Define the indicator(s), populations (including age, sex, and geographic entities), and time period(s) for which estimates were made.	Main Pg. 6, Appendix Pg. 3,9,10
**2**	List the funding sources for the work.	Main Pg. 2, 15
**Data Inputs**		
For all data inputs from multiple sources that are synthesized as part of the study:		
**3**	Describe how the data were identified and how the data were accessed.	Main Pg. 6
**4**	Specify the inclusion and exclusion criteria. Identify all ad-hoc exclusions.	Appendix pg. 11
**5**	Provide information on all included data sources and their main characteristics. For each data source used, report reference information or contact name/institution, population represented, data collection method, year(s) of data collection, sex and age range, diagnostic criteria or measurement method, and sample size, as relevant.	http://ghdx.healthdata.org/gbd-2019/data-input-sources
**6**	Identify and describe any categories of input data that have potentially important biases (e.g., based on characteristics listed in item 5).	http://ghdx.healthdata.org/gbd-2019/data-input-sources
For data inputs that contribute to the analysis but were not synthesized as part of the study:		
**7**	Describe and give sources for any other data inputs.	N/A
For all data inputs:		
**8**	Provide all data inputs in a file format from which data can be efficiently extracted (e.g., a spreadsheet rather than a PDF), including all relevant meta-data listed in item 5. For any data inputs that cannot be shared because of ethical or legal reasons, such as third-party ownership, provide a contact name or the name of the institution that retains the right to the data.	http://ghdx.healthdata.org/gbd-2019/data-input-sources
**Data analysis**		
**9**	Provide a conceptual overview of the data analysis method. A diagram may be helpful.	Main Pg. 6-7
**10**	Provide a detailed description of all steps of the analysis, including mathematical formulae. This description should cover, as relevant, data cleaning, data pre-processing, data adjustments and weighting of data sources, and mathematical or statistical model(s).	Main Pg. 6-7, Appendix Pg. 9-12
**11**	Describe how candidate models were evaluated and how the final model(s) were selected.	Main Pg. 7

**12**	Provide the results of an evaluation of model performance, if done, as well as the results of any relevant sensitivity analysis.	N/A
**13**	Describe methods for calculating uncertainty of the estimates. State which sources of uncertainty were, and were not, accounted for in the uncertainty analysis.	Main Pg. 7
**14**	State how analytic or statistical source code used to generate estimates can be accessed.	Appendix Pg. 17 (https://github.com/ihmeuw/ihme-modeling/tree/master/gbd_2019/risk_factors_code/smoking_direct)
**Results and Discussion**		
**15**	Provide published estimates in a file format from which data can be efficiently extracted.	http://ghdx.healthdata.org/gbd-results-tool
**16**	Report a quantitative measure of the uncertainty of the estimates (e.g. uncertainty intervals).	Main Pg. 8-14; Supplemental Results
**17**	Interpret results in light of existing evidence. If updating a previous set of estimates, describe the reasons for changes in estimates.	Main Pg. 3-5
**18**	Discuss limitations of the estimates. Include a discussion of any modelling assumptions or data limitations that affect interpretation of the estimates.	Main Pg. 13-14
