# Supplementary material for: Spatial, temporal, and demographic patterns in prevalence of smoking tobacco use and initiation among young people in 204 countries and territories, 1990–2019
Source: Lancet Public Health. 2021 May 28;6(7):e472–81. doi: 10.1016/S2468-2667(21)00102-X (PMC8251503; doi:10.1016/S2468-2667(21)00102-X)
Supplement: Supplementary appendix 2 [file mmc2.pdf]

THE LANCET
Public Health

Supplementary appendix 2

This appendix formed part of the original submission and has been peer reviewed. We post it as supplied by the authors.

Supplement to: Reitsma MB, Flor LS, Mullany EC, Gupta V, Hay SI, Gakidou E. Spatial, temporal, and demographic patterns in prevalence of smoking tobacco use and initiation among young people in 204 countries and territories, 1990–2019. *Lancet Public Health* 2021; published online May 27. http://dx.doi.org/10.1016/S2468-2667(21)00102-X.

Supplementary Results

Spatial, temporal, and demographic patterns in prevalence of smoking tobacco use and initiation among young people in 204 countries and territories, 1990-2019

Marissa B Reitsma, ScB[1], Luisa S Flor, PhD[1], Erin Mullany BS[1], Vin Gupta, MD, MSc[1], Simon I Hay DPhil, DSc[1], Emmanuela Gakidou, PhD[1*]

[1]Institute for Health Metrics and Evaluation, Department of Health Metrics Sciences, University of Washington, Seattle, WA, USA

Table of Contents

Supplemental Table S1. Number of Data Sources by Country, for Smoking Prevalence and Initiation Age Indicators. **3**

Supplemental Table S2. Smoking prevalence, by sex, for ages 15-24 in 2019. .... **9**

Supplemental Table S3. Number of Smokers, by sex, for ages 15-24 in 2019. .... **18**

Supplemental Table S4. Percent change in smoking prevalence, by sex, for ages 15-24, from 1990-2019. .... **27**

Supplemental Table S5. Percent change in number of smokers, by sex, for ages 15-24, from 1990-2019. .... **36**

Supplemental Table S6. Absolute change in number of smokers, by sex, for ages 15-24, from 1990-2019. .... **45**

Supplemental Table S7. Age window of initiation and mean age of initiation among current smokers ages 20-54, by sex. Age window of initiation reports the 10th and 90th percentiles of the distribution of initiation ages observed in the population. **54**

Supplemental Figure S1. Absolute difference in mean age of initiation between males and females vs. absolute difference in smoking prevalence between males and females, 2019, by super region. **63**

Supplemental Table S8. Smoking prevalence in 2019, by sex, for ages 15-19 and 20-24. .... **64**

Supplemental Table S1. Number of Data Sources by Country, for Smoking Prevalence and Initiation Age Indicators.

Location	Smoking Prevalence Data Sources	Initiation Age Data Sources
**Central Europe, Eastern Europe, and Central Asia**	**747**	**72**
Armenia	20	2
Azerbaijan	14	1
Georgia	20	4
Kazakhstan	17	1
Kyrgyzstan	21	3
Mongolia	13	2
Tajikistan	10	0
Turkmenistan	5	0
Uzbekistan	8	0
Albania	15	2
Bosnia and Herzegovina	14	0
Bulgaria	19	3
Croatia	26	2
Czechia	43	3
Hungary	34	2
Montenegro	10	0
North Macedonia	17	0
Poland	53	3
Romania	29	4
Serbia	15	0
Slovakia	29	3
Slovenia	28	3
Belarus	13	1
Estonia	67	2
Latvia	47	3
Lithuania	52	2
Republic of Moldova	18	3
Russian Federation	51	20
Ukraine	39	3
**High-income**	**1648**	**100**
Australia	60	0
New Zealand	60	0
Brunei Darussalam	3	0
Japan	55	0
Republic of Korea	17	0
Singapore	13	0
Canada	44	0

Greenland	12	0
United States of America	162	13
Argentina	12	3
Chile	8	0
Uruguay	15	1
Andorra	2	0
Austria	37	3
Belgium	42	3
Cyprus	15	3
Denmark	76	3
Finland	105	3
France	61	3
Germany	56	3
Greece	49	4
Iceland	51	0
Ireland	57	3
Israel	32	0
Italy	80	3
Luxembourg	27	2
Malta	26	3
Monaco	0	0
Netherlands	74	3
Norway	36	0
Portugal	47	3
San Marino	4	0
Spain	64	4
Sweden	77	3
Switzerland	40	0
United Kingdom	129	34
**Latin America and Caribbean**	**317**	**22**
Bolivia (Plurinational State of)	7	0
Ecuador	11	0
Peru	12	0
Antigua and Barbuda	6	0
Bahamas	7	0
Barbados	11	1
Belize	7	0
Bermuda	2	0
Cuba	5	0
Dominica	4	0
Dominican Republic	14	0
Grenada	5	0
Guyana	9	0

Haiti	7	0
Jamaica	13	0
Puerto Rico	20	0
Saint Kitts and Nevis	3	1
Saint Lucia	6	0
Saint Vincent and the Grenadines	6	0
Suriname	5	0
Trinidad and Tobago	12	0
United States Virgin Islands	14	2
Colombia	8	0
Costa Rica	16	1
El Salvador	8	0
Guatemala	10	0
Honduras	8	0
Mexico	23	14
Nicaragua	5	0
Panama	8	0
Venezuela (Bolivarian Republic of)	6	0
Brazil	31	3
Paraguay	8	0
**North Africa and Middle East**	**197**	**7**
Afghanistan	3	0
Algeria	5	0
Bahrain	8	0
Egypt	16	1
Iran (Islamic Republic of)	7	0
Iraq	6	0
Jordan	16	1
Kuwait	10	0
Lebanon	9	0
Libya	6	1
Morocco	11	0
Oman	11	0
Palestine	10	2
Qatar	9	0
Saudi Arabia	8	0
Sudan	6	0
Syrian Arab Republic	7	0
Tunisia	10	0
Turkey	23	2
United Arab Emirates	9	0
Yemen	7	0
**South Asia**	**78**	**4**

Bangladesh	16	1
Bhutan	9	0
India	28	3
Nepal	16	0
Pakistan	9	0
**Southeast Asia, East Asia, and Oceania**	**349**	**24**
China	38	8
Democratic People's Republic of Korea	5	0
Taiwan (Province of China)	13	0
American Samoa	2	1
Cook Islands	3	0
Fiji	8	1
Guam	16	0
Kiribati	6	1
Marshall Islands	3	0
Micronesia (Federated States of)	3	0
Nauru	2	1
Niue	3	0
Northern Mariana Islands	3	0
Palau	6	0
Papua New Guinea	5	0
Samoa	6	0
Solomon Islands	7	0
Tokelau	0	0
Tonga	7	1
Tuvalu	3	0
Vanuatu	8	1
Cambodia	19	1
Indonesia	36	3
Lao People's Democratic Republic	8	0
Malaysia	13	1
Maldives	9	0
Mauritius	11	0
Myanmar	12	0
Philippines	24	2
Seychelles	6	1
Sri Lanka	17	0
Thailand	25	1
Timor-Leste	8	0
Viet Nam	14	1
**Sub-Saharan Africa**	**289**	**25**
Angola	1	0
Central African Republic	0	0

Congo	4	0
Democratic Republic of the Congo	2	0
Equatorial Guinea	2	0
Gabon	2	0
Burundi	3	0
Comoros	5	1
Djibouti	6	0
Eritrea	4	2
Ethiopia	6	0
Kenya	13	2
Madagascar	6	0
Malawi	11	1
Mozambique	6	1
Rwanda	8	0
Somalia	2	0
South Sudan	1	0
Uganda	13	0
United Republic of Tanzania	9	0
Zambia	9	0
Botswana	7	1
Eswatini	9	1
Lesotho	6	0
Namibia	9	2
South Africa	19	2
Zimbabwe	11	0
Benin	9	1
Burkina Faso	7	0
Cabo Verde	4	1
Cameroon	6	1
Chad	5	0
Côte d'Ivoire	3	0
Gambia	3	1
Ghana	13	0
Guinea	2	0
Guinea-Bissau	0	0
Liberia	6	1
Mali	6	0
Mauritania	7	0
Niger	9	2
Nigeria	8	1
São Tomé and Príncipe	4	2
Senegal	11	0
Sierra Leone	6	1

Togo	6	1

Supplemental Table S2. Smoking prevalence, by sex, for ages 15-24 in 2019.

**Location**	**Smoking Prevalence Ages 15-24 2019 Male (%)**	**Smoking Prevalence Ages 15-24 2019 Female (%)**	**Smoking Prevalence Ages 15-24 2019 Both (%)**
**Global**	**20·1 (19·4–20·8)**	**4·95 (4·64–5·29)**	**12·7 (12·3–13·1)**
**Central Europe, Eastern Europe, and Central Asia**	**27·8 (26·7–29·0)**	**14·7 (13·5–16·1)**	**21·4 (20·6–22·3)**
Armenia	38·3 (34·9–42·1)	1·87 (0·993–3·21)	20·9 (19·0–22·9)
Azerbaijan	28·4 (24·9–31·9)	2·41 (1·27–4·03)	15·9 (14·0–18·0)
Georgia	40·1 (36·1–44·3)	7·14 (4·89–9·82)	24·6 (22·2–27·1)
Kazakhstan	24·3 (20·8–28·0)	7·20 (4·61–10·6)	15·8 (13·6–18·4)
Kyrgyzstan	23·4 (19·5–27·5)	3·98 (2·35–6·48)	13·8 (11·6–16·0)
Mongolia	35·4 (30·7–40·2)	5·21 (3·18–8·10)	20·6 (17·9–23·2)
Tajikistan	8·93 (7·06–11·0)	1·01 (0·500–1·88)	5·05 (4·05–6·14)
Turkmenistan	16·7 (13·1–20·6)	1·65 (0·883–2·88)	9·61 (7·69–11·6)
Uzbekistan	8·76 (6·73–11·2)	1·32 (0·653–2·45)	5·11 (4·01–6·38)
Albania	44·1 (40·5–47·9)	12·4 (7·95–18·3)	29·0 (26·0–32·1)
Bosnia and Herzegovina	31·3 (26·8–36·2)	24·4 (17·2–32·5)	27·9 (23·6–32·6)
Bulgaria	37·7 (33·0–42·2)	39·7 (32·5–47·1)	38·7 (34·3–42·9)
Croatia	37·1 (33·1–41·5)	34·9 (28·8–41·0)	36·0 (32·3–40·1)
Czechia	35·2 (31·5–38·8)	29·3 (24·6–34·1)	32·3 (29·3–35·4)
Hungary	32·8 (28·2–37·9)	29·7 (23·1–37·4)	31·3 (27·0–35·8)
Montenegro	20·2 (17·5–23·0)	16·4 (12·8–20·5)	18·4 (16·1–20·7)
North Macedonia	37·7 (33·3–42·3)	23·8 (18·1–30·0)	31·0 (27·5–35·0)
Poland	28·5 (25·6–31·8)	24·8 (20·7–29·8)	26·7 (24·0–29·6)
Romania	34·3 (30·7–37·9)	22·3 (18·2–27·3)	28·4 (25·6–31·3)
Serbia	27·5 (24·0–31·0)	33·5 (27·4–39·9)	30·4 (27·1–33·8)

Slovakia	32·4 (28·1–36·9)	28·3 (22·5–34·2)	30·4 (27·0–34·0)
Slovenia	32·7 (28·8–36·9)	28·4 (22·6–33·9)	30·6 (27·3–34·0)
Belarus	36·4 (31·4–41·2)	26·0 (19·5–33·3)	31·4 (27·0–35·9)
Estonia	28·6 (25·4–31·9)	20·7 (16·5–25·3)	24·7 (22·0–27·6)
Latvia	39·9 (36·0–43·6)	27·4 (22·1–33·0)	33·8 (30·4–37·0)
Lithuania	34·6 (30·7–38·5)	28·6 (24·1–34·5)	31·7 (28·6–35·1)
Republic of Moldova	34·4 (30·2–38·8)	8·56 (5·57–12·6)	21·8 (19·1–24·7)
Russian Federation	31·6 (28·7–34·4)	14·7 (11·5–18·2)	23·3 (21·1–25·8)
Ukraine	35·3 (31·4–39·8)	18·1 (13·8–23·7)	26·9 (23·8–30·1)
**High-income**	**24·2 (23·1–25·1)**	**17·2 (15·9–18·5)**	**20·8 (19·9–21·6)**
Australia	15·7 (12·6–19·2)	14·5 (9·84–20·7)	15·1 (12·3–18·8)
New Zealand	20·8 (18·5–23·3)	15·9 (13·0–19·3)	18·4 (16·6–20·5)
Brunei Darussalam	28·1 (22·4–34·2)	5·78 (3·28–9·30)	17·7 (14·4–21·1)
Japan	22·7 (18·6–27·1)	6·60 (3·95–10·3)	14·9 (12·2–17·8)
Republic of Korea	29·7 (24·6–34·9)	7·57 (4·48–12·1)	19·2 (16·1–22·4)
Singapore	17·3 (13·8–21·1)	7·47 (4·57–11·6)	12·4 (10·0–15·1)
Canada	17·1 (13·8–20·8)	15·0 (10·0–21·3)	16·1 (13·1–19·5)
Greenland	49·2 (41·7–57·2)	49·0 (37·1–62·1)	49·1 (41·9–56·3)
United States of America	18·4 (16·3–21·0)	13·3 (10·3–16·4)	15·9 (14·2–17·9)
Argentina	29·8 (25·4–33·9)	17·7 (12·5–23·9)	23·8 (20·5–27·3)
Chile	37·3 (32·1–42·1)	39·2 (29·8–49·2)	38·2 (32·9–43·6)
Uruguay	27·6 (22·8–32·4)	24·9 (17·3–33·9)	26·2 (21·6–31·4)
Andorra	29·6 (22·9–36·1)	27·6 (16·6–41·2)	28·6 (22·1–35·6)
Austria	37·2 (32·1–42·5)	26·3 (20·2–33·7)	32·0 (27·7–36·3)
Belgium	22·9 (19·9–26·1)	20·7 (16·4–25·6)	21·8 (19·2–24·6)
Cyprus	41·0 (35·2–46·6)	18·5 (12·5–26·0)	30·2 (26·0–34·7)
Denmark	21·1 (18·0–24·3)	21·7 (16·8–27·1)	21·4 (18·6–24·4)

Finland	23·5 (20·3–26·7)	23·2 (18·2–29·4)	23·4 (20·4–26·7)
France	38·0 (33·8–42·1)	34·8 (28·8–41·1)	36·4 (32·9–40·3)
Germany	29·7 (26·0–33·3)	23·5 (18·9–29·1)	26·8 (24·0–29·8)
Greece	36·4 (32·2–40·5)	24·2 (18·4–31·1)	30·4 (26·6–34·4)
Iceland	14·5 (11·9–17·3)	14·0 (9·87–19·6)	14·2 (11·9–17·2)
Ireland	20·6 (17·1–24·4)	28·0 (21·5–34·8)	24·2 (20·3–28·1)
Israel	24·3 (20·6–28·2)	12·7 (8·40–18·0)	18·6 (15·8–22·1)
Italy	29·2 (25·8–32·7)	18·7 (15·4–22·6)	24·2 (21·6–26·9)
Luxembourg	28·1 (23·9–32·3)	33·7 (27·5–39·8)	30·8 (27·3–34·4)
Malta	23·1 (18·9–27·7)	24·2 (17·9–31·6)	23·6 (19·7–27·8)
Monaco	27·3 (21·3–33·5)	25·2 (14·9–37·8)	26·3 (20·2–33·3)
Netherlands	24·8 (21·5–28·4)	22·6 (18·0–27·9)	23·7 (20·8–27·0)
Norway	16·5 (13·6–20·2)	14·0 (9·95–19·2)	15·3 (12·8–18·3)
Portugal	34·9 (30·7–39·1)	29·0 (23·3–34·9)	32·0 (28·5–35·6)
San Marino	21·2 (17·7–25·4)	18·9 (13·2–26·3)	20·0 (16·5–24·1)
Spain	28·1 (24·6–31·7)	26·1 (21·1–31·6)	27·1 (24·1–30·3)
Sweden	14·2 (11·3–17·3)	16·4 (11·1–22·5)	15·3 (12·5–18·5)
Switzerland	27·3 (23·1–31·7)	20·5 (14·9–27·3)	24·0 (20·4–27·9)
United Kingdom	22·3 (19·2–25·7)	19·5 (14·9–24·4)	20·9 (18·3–23·8)
**Latin America and Caribbean**	**16·1 (15·2–17·0)**	**7·15 (6·19–8·25)**	**11·7 (11·0–12·4)**
Bolivia (Plurinational State of)	17·8 (14·7–21·5)	8·99 (5·84–12·9)	13·5 (11·2–16·0)
Ecuador	21·3 (18·6–24·1)	5·12 (3·37–7·55)	13·3 (11·7–15·2)
Peru	12·4 (10·1–15·3)	5·87 (3·76–8·55)	9·27 (7·70–11·2)
Antigua and Barbuda	8·77 (6·70–11·3)	6·39 (4·13–9·37)	7·60 (6·00–9·49)
Bahamas	12·7 (10·4–15·4)	4·39 (2·57–6·84)	8·51 (7·07–10·3)
Barbados	13·1 (10·1–16·4)	8·17 (5·19–12·2)	10·7 (8·43–13·3)
Belize	20·0 (16·0–24·3)	5·83 (3·38–9·07)	12·8 (10·5–15·6)

Bermuda	15·0 (11·7–19·1)	6·84 (4·14–10·8)	10·9 (8·71–13·6)
Cuba	18·6 (15·1–22·6)	11·8 (8·15–16·4)	15·3 (12·6–18·3)
Dominica	12·9 (9·88–16·4)	8·63 (4·99–13·7)	10·9 (8·27–14·0)
Dominican Republic	5·99 (4·63–7·58)	3·89 (2·39–5·96)	4·95 (3·91–6·27)
Grenada	14·5 (11·4–18·1)	6·64 (4·06–10·1)	10·8 (8·64–13·1)
Guyana	21·6 (18·0–25·1)	4·87 (2·98–7·63)	13·3 (11·4–15·6)
Haiti	5·85 (4·48–7·55)	2·65 (1·47–4·46)	4·23 (3·26–5·31)
Jamaica	15·2 (12·6–17·9)	9·54 (6·91–12·9)	12·4 (10·6–14·5)
Puerto Rico	12·8 (10·2–15·5)	6·06 (3·88–8·83)	9·43 (7·69–11·3)
Saint Kitts and Nevis	7·64 (5·57–10·2)	2·66 (1·33–4·78)	5·13 (3·84–6·71)
Saint Lucia	15·3 (12·0–19·5)	7·83 (5·05–11·5)	11·6 (9·48–14·2)
Saint Vincent and the Grenadines	21·7 (17·9–26·0)	7·28 (4·41–11·3)	14·7 (12·2–17·6)
Suriname	28·8 (24·4–34·0)	9·00 (5·69–13·2)	19·1 (16·1–22·1)
Trinidad and Tobago	26·5 (22·3–31·3)	9·82 (6·61–14·3)	18·3 (15·6–21·3)
United States Virgin Islands	4·72 (3·45–6·31)	2·28 (1·07–4·16)	3·50 (2·59–4·63)
Colombia	16·6 (13·7–19·8)	9·08 (6·32–12·6)	12·9 (10·8–15·3)
Costa Rica	15·2 (12·4–18·5)	8·44 (5·57–12·4)	11·8 (9·84–14·4)
El Salvador	19·6 (16·1–23·3)	5·79 (3·85–8·18)	12·6 (10·7–14·8)
Guatemala	22·8 (19·5–26·2)	7·24 (4·72–10·5)	15·1 (12·9–17·7)
Honduras	23·8 (19·9–27·9)	6·53 (4·18–9·43)	15·0 (12·7–17·5)
Mexico	27·0 (24·0–29·9)	10·1 (7·25–13·4)	18·5 (16·5–20·8)
Nicaragua	22·3 (18·3–27·3)	8·19 (5·10–12·1)	15·3 (12·8–18·3)
Panama	10·7 (8·49–13·3)	6·39 (3·95–10·1)	8·59 (6·90–10·7)
Venezuela (Bolivarian Republic of)	18·2 (14·1–22·8)	7·33 (4·15–11·6)	12·7 (10·2–15·8)
Brazil	8·63 (7·21–10·3)	5·36 (3·54–7·46)	7·01 (5·85–8·31)
Paraguay	16·3 (12·8–20·1)	7·27 (4·32–11·5)	11·9 (9·53–14·7)
**North Africa and Middle East**	**24·9 (24·0–25·9)**	**5·60 (4·94–6·35)**	**15·6 (15·0–16·2)**

Afghanistan	15·1 (12·2–18·2)	2·51 (1·34–4·23)	8·97 (7·29–10·7)
Algeria	26·3 (22·3–30·5)	1·64 (0·879–2·78)	14·2 (12·1–16·4)
Bahrain	26·3 (22·1–31·3)	6·86 (4·58–9·96)	16·9 (14·3–20·1)
Egypt	27·3 (24·8–29·9)	1·68 (0·909–2·83)	14·8 (13·5–16·3)
Iran (Islamic Republic of)	14·2 (11·4–17·4)	3·73 (2·11–5·96)	9·09 (7·47–10·9)
Iraq	26·5 (22·4–30·6)	3·42 (1·99–5·52)	15·3 (13·0–17·5)
Jordan	44·4 (40·8–47·8)	15·0 (10·7–20·4)	30·7 (27·9–33·8)
Kuwait	38·4 (33·7–43·6)	7·76 (5·31–10·6)	23·6 (20·9–26·7)
Lebanon	35·2 (31·0–39·8)	13·3 (9·31–18·0)	24·9 (22·0–28·3)
Libya	23·6 (19·1–28·7)	2·14 (1·08–3·64)	13·1 (10·8–15·9)
Morocco	14·8 (12·1–17·7)	1·77 (0·992–2·93)	8·35 (6·87–9·88)
Oman	11·5 (8·77–14·6)	1·93 (1·03–3·25)	7·66 (5·94–9·57)
Palestine	28·9 (23·8–34·1)	3·22 (1·80–5·34)	16·4 (13·6–19·1)
Qatar	21·5 (17·4–26·3)	6·28 (3·73–9·80)	18·1 (14·9–21·8)
Saudi Arabia	20·9 (17·8–24·5)	2·54 (1·40–4·23)	12·8 (11·1–14·9)
Sudan	13·5 (11·0–16·5)	2·03 (1·06–3·57)	7·92 (6·55–9·65)
Syrian Arab Republic	29·5 (24·2–35·2)	6·72 (3·70–10·8)	17·6 (14·5–21·1)
Tunisia	37·2 (32·7–41·8)	2·71 (1·55–4·42)	20·1 (17·8–22·6)
Turkey	45·6 (42·2–48·9)	22·0 (18·2–26·4)	34·2 (31·6–36·8)
United Arab Emirates	17·1 (14·0–20·5)	4·08 (2·47–6·54)	10·9 (9·09–13·0)
Yemen	18·5 (15·2–22·3)	5·17 (3·05–8·32)	12·0 (9·92–14·3)
**South Asia**	**13·8 (12·1–15·6)**	**1·91 (1·24–2·77)**	**8·00 (7·05–9·06)**
Bangladesh	26·1 (22·7–30·0)	1·14 (0·599–1·92)	13·0 (11·3–15·0)
Bhutan	22·1 (18·3–26·1)	7·31 (4·61–10·7)	15·0 (12·5–17·7)
India	12·6 (10·5–14·9)	1·98 (1·13–3·13)	7·52 (6·32–8·80)
Nepal	20·0 (17·1–22·9)	3·01 (1·74–4·95)	11·1 (9·58–12·9)
Pakistan	11·9 (9·66–14·3)	1·83 (1·01–3·14)	7·01 (5·79–8·31)

**Southeast Asia, East Asia, and Oceania**	**32·0 (30·2–34·0)**	**2·69 (2·16–3·50)**	**17·9 (16·9–19·0)**
China	30·1 (27·0–33·2)	2·17 (1·34–3·55)	16·9 (15·2–18·7)
Democratic People's Republic of Korea	26·0 (21·4–30·9)	2·41 (1·20–4·21)	14·4 (12·0–17·0)
Taiwan (Province of China)	24·6 (20·3–28·8)	4·52 (2·32–7·93)	15·0 (12·5–17·7)
American Samoa	37·7 (31·0–44·6)	21·3 (12·4–32·7)	29·6 (24·1–35·9)
Cook Islands	35·9 (30·2–41·8)	34·4 (25·0–45·5)	35·1 (29·3–41·8)
Fiji	36·7 (30·8–42·8)	15·0 (9·62–22·4)	26·1 (21·9–31·1)
Guam	28·0 (24·6–31·7)	16·3 (12·3–20·9)	22·5 (19·8–25·5)
Kiribati	54·7 (49·7–59·5)	25·9 (19·4–33·3)	40·2 (36·1–44·8)
Marshall Islands	40·0 (34·4–45·5)	12·2 (8·00–17·4)	26·3 (22·9–30·2)
Micronesia (Federated States of)	63·6 (57·4–69·2)	38·4 (28·1–49·1)	51·3 (45·5–57·7)
Nauru	45·5 (39·6–51·2)	41·5 (31·5–52·7)	43·6 (37·9–50·3)
Niue	19·4 (15·8–23·7)	14·5 (9·27–21·7)	17·1 (13·8–20·9)
Northern Mariana Islands	37·4 (31·1–43·6)	20·6 (13·9–29·2)	29·3 (24·7–34·7)
Palau	39·3 (34·9–43·8)	23·2 (19·2–27·9)	32·1 (29·0–35·1)
Papua New Guinea	46·7 (41·3–52·0)	23·0 (17·1–30·0)	35·4 (31·2–39·8)
Samoa	26·3 (22·8–30·0)	9·89 (6·71–13·8)	18·4 (15·9–21·0)
Solomon Islands	46·5 (42·1–50·9)	18·9 (14·5–24·1)	33·0 (30·0–36·1)
Tokelau	36·2 (29·3–43·7)	16·8 (8·81–26·4)	26·8 (21·3–32·7)
Tonga	33·6 (29·2–38·4)	13·4 (9·12–18·6)	23·7 (20·5–27·1)
Tuvalu	40·2 (34·8–46·0)	18·3 (12·2–25·3)	29·9 (25·7–34·3)
Vanuatu	36·4 (31·6–41·3)	10·5 (6·71–14·8)	23·3 (20·2–26·5)
Cambodia	14·9 (12·3–17·9)	2·14 (1·20–3·58)	8·65 (7·20–10·3)
Indonesia	41·7 (38·6–45·1)	2·24 (1·32–3·54)	22·3 (20·6–24·0)
Lao People's Democratic Republic	28·7 (24·2–33·3)	3·46 (2·03–5·36)	16·2 (13·8–18·6)
Malaysia	33·6 (29·3–37·9)	3·02 (1·82–4·79)	18·8 (16·4–21·1)
Maldives	36·3 (30·7–41·8)	5·62 (3·39–8·52)	23·3 (19·7–26·6)

Mauritius	41·6 (36·8–46·3)	9·04 (6·23–12·7)	25·5 (22·7–28·3)
Myanmar	33·3 (29·5–37·2)	1·76 (0·935–2·91)	17·4 (15·5–19·4)
Philippines	33·1 (29·7–36·7)	6·70 (4·70–9·32)	20·2 (18·2–22·3)
Seychelles	37·8 (31·0–44·2)	9·77 (6·45–13·8)	24·5 (20·6–28·3)
Sri Lanka	16·2 (13·5–19·3)	0·911 (0·464–1·69)	8·54 (7·19–10·2)
Thailand	34·0 (30·5–37·4)	2·88 (1·76–4·59)	18·6 (16·7–20·5)
Timor-Leste	52·8 (48·8–57·0)	3·80 (2·31–5·97)	28·5 (26·3–31·0)
Viet Nam	26·9 (23·6–30·4)	1·11 (0·569–2·00)	14·3 (12·6–16·1)
**Sub-Saharan Africa**	**9·69 (9·26–10·1)**	**2·12 (1·85–2·42)**	**5·83 (5·57–6·09)**
Angola	7·78 (6·03–10·0)	1·18 (0·583–2·07)	4·38 (3·44–5·50)
Central African Republic	9·49 (7·04–12·3)	1·26 (0·622–2·42)	5·26 (4·02–6·72)
Congo	14·1 (10·9–17·8)	2·43 (1·20–4·34)	8·16 (6·41–10·2)
Democratic Republic of the Congo	10·3 (7·99–13·0)	0·551 (0·288–0·987)	5·45 (4·27–6·82)
Equatorial Guinea	23·0 (18·5–27·9)	4·95 (2·51–8·71)	15·5 (12·6–18·9)
Gabon	19·5 (16·1–23·3)	5·64 (3·34–9·01)	12·2 (10·2–14·7)
Burundi	5·99 (4·47–7·89)	1·12 (0·571–2·06)	3·44 (2·64–4·42)
Comoros	17·6 (14·3–21·2)	4·04 (2·37–6·45)	10·9 (8·89–13·1)
Djibouti	20·7 (16·7–24·8)	5·04 (2·94–8·32)	13·7 (11·3–16·4)
Eritrea	7·18 (5·27–9·68)	0·745 (0·373–1·40)	4·03 (3·03–5·31)
Ethiopia	4·18 (3·15–5·43)	0·722 (0·387–1·29)	2·47 (1·91–3·13)
Kenya	9·38 (7·43–11·5)	2·09 (1·17–3·36)	5·74 (4·57–6·95)
Madagascar	23·8 (20·0–28·0)	2·84 (1·56–4·75)	13·3 (11·2–15·7)
Malawi	8·84 (7·13–10·9)	1·89 (1·03–3·21)	5·26 (4·30–6·43)
Mozambique	10·2 (7·83–12·9)	3·77 (2·11–6·07)	6·78 (5·26–8·49)
Rwanda	6·56 (5·00–8·54)	0·775 (0·384–1·40)	3·63 (2·79–4·67)
Somalia	10·5 (7·79–14·0)	2·12 (1·09–3·84)	6·48 (4·88–8·42)
South Sudan	10·9 (8·04–14·1)	1·70 (0·848–3·00)	6·33 (4·82–8·07)

Uganda	7·80 (6·16–9·64)	3·36 (1·96–5·54)	5·55 (4·43–6·90)
United Republic of Tanzania	7·66 (6·03–9·66)	1·96 (1·08–3·33)	4·68 (3·74–5·84)
Zambia	16·3 (13·9–19·1)	7·08 (4·50–10·7)	11·5 (9·64–13·8)
Botswana	25·0 (21·2–29·1)	7·50 (4·96–10·8)	16·2 (13·8–18·8)
Eswatini	9·51 (7·43–12·0)	1·78 (0·908–3·06)	5·62 (4·49–6·99)
Lesotho	31·5 (27·2–35·6)	1·58 (0·775–2·93)	16·6 (14·5–18·8)
Namibia	17·1 (14·2–20·6)	8·32 (5·23–12·8)	12·6 (10·3–15·3)
South Africa	27·9 (24·7–31·5)	12·7 (8·32–17·7)	20·4 (17·7–23·3)
Zimbabwe	17·2 (14·4–20·6)	3·04 (1·64–5·07)	9·91 (8·35–11·8)
Benin	5·78 (4·39–7·45)	1·26 (0·692–2·14)	3·44 (2·61–4·29)
Burkina Faso	13·2 (10·4–16·3)	1·45 (0·749–2·60)	7·15 (5·73–8·74)
Cabo Verde	5·38 (3·88–7·07)	1·69 (0·879–2·91)	3·56 (2·69–4·58)
Cameroon	7·10 (5·48–9·04)	1·55 (0·844–2·52)	4·29 (3·39–5·33)
Chad	9·85 (7·64–12·5)	2·53 (1·31–4·53)	6·09 (4·73–7·69)
Côte d'Ivoire	19·3 (15·5–23·4)	3·58 (1·96–6·02)	11·6 (9·39–14·1)
Gambia	17·0 (14·1–20·3)	1·81 (0·973–3·07)	9·19 (7·73–10·8)
Ghana	4·11 (3·09–5·27)	1·23 (0·632–2·05)	2·64 (2·04–3·34)
Guinea	20·0 (15·7–24·8)	1·57 (0·762–2·85)	10·3 (8·19–12·6)
Guinea-Bissau	4·97 (3·58–6·71)	0·777 (0·392–1·42)	2·82 (2·09–3·74)
Liberia	4·89 (3·65–6·63)	3·07 (1·77–4·82)	3·97 (3·01–5·23)
Mali	16·3 (13·0–19·9)	1·05 (0·527–1·78)	8·55 (6·88–10·2)
Mauritania	26·5 (21·8–31·6)	9·68 (6·50–13·8)	17·8 (15·0–21·0)
Niger	14·0 (11·2–17·3)	2·18 (1·18–3·65)	7·92 (6·41–9·61)
Nigeria	3·72 (2·82–4·89)	0·489 (0·244–0·905)	2·01 (1·57–2·52)
São Tomé and Príncipe	2·61 (1·86–3·60)	0·558 (0·273–1·01)	1·58 (1·16–2·12)
Senegal	11·4 (9·35–13·8)	1·56 (0·844–2·72)	6·63 (5·47–7·94)
Sierra Leone	17·2 (14·0–20·8)	7·31 (4·41–11·6)	12·1 (9·96–14·8)

Togo	7·32<br>(5·67–9·28)	1·42<br>(0·744–2·37)	4·43<br>(3·49–5·53)

Supplemental Table S3. Number of Smokers, by sex, for ages 15-24 in 2019.

Location	Number of Smokers Ages 15-24 2019 Male (Thousands)	Number of Smokers Ages 15-24 2019 Female (Thousands)	Number of Smokers Ages 15-24 2019 Both (Thousands)
**Global**	**125000 (121000–130000)**	**29600 (27700–31600)**	**155000 (150000–160000)**
**Central Europe, Eastern Europe, and Central Asia**	**6720 (6440–7000)**	**3390 (3100–3700)**	**10100 (9700–10500)**
Armenia	71·1 (64·8–78·2)	3·17 (1·68–5·44)	74·3 (67·4–81·5)
Azerbaijan	216 (190–243)	16·9 (8·92–28·3)	233 (205–263)
Georgia	86·5 (77·9–95·7)	13·7 (9·40–18·9)	100 (90·7–111)
Kazakhstan	299 (257–345)	87·2 (55·8–128)	386 (331–448)
Kyrgyzstan	130 (108–153)	21·5 (12·7–35·1)	151 (127–176)
Mongolia	83·4 (72·2–94·8)	11·9 (7·23–18·4)	95·3 (82·9–108)
Tajikistan	79·7 (63·1–98·7)	8·60 (4·27–16·1)	88·4 (70·9–107)
Turkmenistan	71·5 (56·2–88·3)	6·31 (3·37–11·0)	77·8 (62·2–94·1)
Uzbekistan	260 (200–332)	37·7 (18·7–70·1)	297 (233–372)
Albania	94·4 (86·6–103)	24·4 (15·6–35·8)	119 (107–132)
Bosnia and Herzegovina	64·3 (55·1–74·3)	47·7 (33·7–63·6)	112 (94·7–130)
Bulgaria	121 (105–135)	119 (97·9–142)	240 (213–266)
Croatia	87·7 (78·1–98·0)	78·6 (64·9–92·4)	166 (149–185)
Czechia	171 (153–189)	135 (113–157)	306 (277–335)
Hungary	169 (145–195)	145 (112–182)	314 (271–359)
Montenegro	8·54 (7·42–9·73)	6·47 (5·02–8·06)	15·0 (13·1–16·9)
North Macedonia	52·0 (46·0–58·4)	30·4 (23·2–38·4)	82·4 (73·1–93·0)
Poland	568 (510–635)	473 (394–567)	1040 (935–1150)
Romania	354 (317–392)	218 (178–267)	572 (515–630)
Serbia	150 (131–170)	171 (140–204)	322 (287–358)

Slovakia	93·2 (80·9–106)	77·5 (61·7–93·7)	171 (151–191)
Slovenia	32·1 (28·3–36·3)	26·2 (20·9–31·3)	58·3 (52·0–64·9)
Belarus	170 (147–193)	114 (85·7–146)	284 (245–326)
Estonia	18·3 (16·3–20·5)	12·6 (10·1–15·5)	31·0 (27·6–34·5)
Latvia	35·8 (32·2–39·1)	23·2 (18·7–28·0)	59·0 (53·0–64·5)
Lithuania	53·6 (47·6–59·8)	41·9 (35·3–50·6)	95·5 (86·3–106)
Republic of Moldova	71·3 (62·6–80·5)	16·9 (11·0–24·9)	88·2 (77·5–99·9)
Russian Federation	2340 (2120–2550)	1040 (815–1290)	3380 (3060–3740)
Ukraine	773 (689–872)	377 (288–492)	1150 (1010–1280)
**High-income**	**15900 (15200–16500)**	**10700 (9930–11500)**	**26600 (25500–27700)**
Australia	241 (194–296)	215 (146–306)	456 (372–568)
New Zealand	61·6 (54·7–69·0)	45·7 (37·4–55·3)	107 (96·5–119)
Brunei Darussalam	11·3 (9·00–13·7)	2·03 (1·15–3·27)	13·3 (10·9–15·9)
Japan	1420 (1160–1690)	391 (234–608)	1810 (1480–2170)
Republic of Korea	945 (780–1110)	218 (129–349)	1160 (975–1360)
Singapore	46·6 (37·2–56·9)	19·7 (12·1–30·7)	66·3 (53·5–80·8)
Canada	370 (298–450)	310 (206–438)	679 (554–823)
Greenland	1·92 (1·63–2·23)	1·87 (1·42–2·37)	3·79 (3·24–4·34)
United States of America	4070 (3610–4630)	2800 (2180–3470)	6880 (6130–7750)
Argentina	1070 (915–1220)	630 (445–853)	1700 (1470–1960)
Chile	494 (426–558)	501 (381–628)	995 (857–1130)
Uruguay	70·6 (58·3–83·1)	62·3 (43·5–85·0)	133 (109–159)
Andorra	1·31 (1·01–1·60)	1·13 (0·678–1·68)	2·43 (1·88–3·03)
Austria	192 (166–219)	125 (96·1–160)	317 (274–360)
Belgium	150 (131–171)	131 (104–162)	281 (247–316)
Cyprus	31·5 (27·0–35·8)	13·3 (9·01–18·7)	44·9 (38·6–51·7)
Denmark	78·7 (67·2–90·8)	77·0 (59·8–96·1)	156 (135–178)

Finland	73·5<br>(63·5–83·5)	68·8<br>(53·9–87·2)	142<br>(124–163)
France	1540<br>(1370–1710)	1350<br>(1120–1600)	2900<br>(2620–3200)
Germany	1410<br>(1240–1580)	989<br>(795–1220)	2400<br>(2150–2670)
Greece	188<br>(167–210)	122<br>(92·3–156)	310<br>(271–351)
Iceland	3·35<br>(2·76–4·00)	3·12<br>(2·20–4·35)	6·47<br>(5·41–7·80)
Ireland	63·8<br>(53·0–75·5)	83·8<br>(64·3–104)	148<br>(124–171)
Israel	172<br>(146–200)	86·0<br>(57·1–122)	258<br>(219–306)
Italy	885<br>(784–991)	527<br>(434–636)	1410<br>(1260–1570)
Luxembourg	10·4<br>(8·79–11·9)	11·8<br>(9·65–14·0)	22·1<br>(19·6–24·7)
Malta	5·38<br>(4·41–6·45)	5·36<br>(3·96–7·00)	10·7<br>(8·96–12·6)
Monaco	0·502<br>(0·391–0·617)	0·439<br>(0·260–0·660)	0·942<br>(0·724–1·19)
Netherlands	264<br>(230–303)	231<br>(184–285)	495<br>(435–563)
Norway	56·5<br>(46·5–68·9)	44·9<br>(31·9–61·4)	101<br>(85·0–121)
Portugal	204<br>(179–228)	163<br>(131–196)	367<br>(326–408)
San Marino	0·436<br>(0·365–0·524)	0·395<br>(0·276–0·550)	0·831<br>(0·686–1·00)
Spain	642<br>(562–724)	568<br>(458–687)	1210<br>(1080–1350)
Sweden	83·3<br>(66·4–101)	88·8<br>(60·2–122)	172<br>(140–208)
Switzerland	131<br>(111–153)	91·8<br>(66·6–122)	223<br>(189–259)
United Kingdom	885<br>(765–1020)	747<br>(569–936)	1630<br>(1430–1860)
**Latin America and Caribbean**	**7910<br>(7450–8370)**	**3460<br>(2990–3990)**	**11400<br>(10700–12000)**
Bolivia (Plurinational State of)	192<br>(158–231)	95·0<br>(61·7–136)	287<br>(238–341)
Ecuador	352<br>(307–398)	82·4<br>(54·3–122)	434<br>(382–495)
Peru	371<br>(303–457)	161<br>(103–235)	532<br>(442–642)
Antigua and Barbuda	0·626<br>(0·478–0·810)	0·440<br>(0·285–0·646)	1·07<br>(0·842–1·33)
Bahamas	3·88<br>(3·20–4·72)	1·36<br>(0·795–2·12)	5·24<br>(4·36–6·37)
Barbados	2·58<br>(2·00–3·23)	1·57<br>(1·00–2·35)	4·15<br>(3·28–5·18)
Belize	8·36<br>(6·69–10·2)	2·49<br>(1·45–3·88)	10·9<br>(8·91–13·2)

Bermuda	0·456 (0·357–0·580)	0·212 (0·128–0·335)	0·668 (0·535–0·833)
Cuba	135 (109–163)	79·5 (54·9–111)	214 (176–255)
Dominica	0·739 (0·564–0·939)	0·460 (0·266–0·731)	1·20 (0·913–1·55)
Dominican Republic	58·7 (45·4–74·3)	37·8 (23·2–57·9)	96·5 (76·3–122)
Grenada	1·39 (1·09–1·73)	0·572 (0·350–0·868)	1·96 (1·57–2·38)
Guyana	16·8 (14·0–19·5)	3·71 (2·26–5·80)	20·5 (17·5–24·1)
Haiti	67·4 (51·6–87·0)	31·4 (17·4–53·0)	98·9 (76·2–124)
Jamaica	39·7 (33·0–46·9)	24·1 (17·4–32·5)	63·8 (54·5–74·7)
Puerto Rico	29·6 (23·7–35·9)	13·9 (8·88–20·2)	43·5 (35·5–52·2)
Saint Kitts and Nevis	0·336 (0·245–0·451)	0·119 (0·0595–0·213)	0·455 (0·341–0·595)
Saint Lucia	2·13 (1·67–2·72)	1·06 (0·685–1·56)	3·19 (2·61–3·91)
Saint Vincent and the Grenadines	2·03 (1·67–2·44)	0·639 (0·387–0·990)	2·67 (2·22–3·18)
Suriname	13·5 (11·4–15·9)	4·08 (2·58–5·98)	17·6 (14·8–20·3)
Trinidad and Tobago	23·9 (20·1–28·3)	8·58 (5·78–12·5)	32·5 (27·7–37·9)
United States Virgin Islands	0·290 (0·212–0·388)	0·139 (0·0656–0·254)	0·429 (0·317–0·568)
Colombia	682 (562–813)	363 (252–501)	1040 (876–1240)
Costa Rica	57·1 (46·8–69·7)	32·2 (21·3–47·3)	89·4 (74·6–109)
El Salvador	116 (95·6–138)	35·3 (23·5–49·8)	152 (129–178)
Guatemala	432 (369–496)	135 (87·9–196)	567 (484–663)
Honduras	241 (202–283)	68·4 (43·8–98·8)	310 (262–360)
Mexico	2910 (2590–3230)	1100 (785–1450)	4010 (3560–4500)
Nicaragua	141 (115–172)	50·1 (31·2–73·9)	191 (158–227)
Panama	38·4 (30·5–47·7)	22·0 (13·6–34·8)	60·4 (48·5–75·1)
Venezuela (Bolivarian Republic of)	387 (301–487)	159 (90·0–252)	546 (437–679)
Brazil	1480 (1230–1760)	900 (594–1250)	2380 (1980–2820)
Paraguay	107 (84·1–132)	45·9 (27·2–72·8)	153 (123–189)
**North Africa and Middle East**	**13400 (12900–14000)**	**2830 (2500–3210)**	**16300 (15600–16900)**

Afghanistan	628 (507–758)	99·2 (53·0–167)	727 (591–871)
Algeria	841 (711–975)	50·6 (27·1–85·9)	891 (757–1030)
Bahrain	22·5 (18·9–26·9)	5·46 (3·64–7·92)	28·0 (23·7–33·2)
Egypt	2520 (2290–2770)	147 (79·5–248)	2670 (2430–2940)
Iran (Islamic Republic of)	818 (657–1000)	206 (116–328)	1020 (841–1230)
Iraq	1190 (1010–1370)	146 (84·5–235)	1340 (1140–1530)
Jordan	542 (498–583)	159 (114–217)	701 (638–771)
Kuwait	106 (92·5–120)	20·1 (13·7–27·5)	126 (111–143)
Lebanon	130 (114–147)	43·5 (30·4–58·8)	173 (153–196)
Libya	142 (115–173)	12·2 (6·19–20·9)	154 (127–186)
Morocco	456 (373–546)	53·2 (29·8–88·2)	509 (418–602)
Oman	46·1 (35·3–58·8)	5·15 (2·75–8·70)	51·3 (39·8–64·1)
Palestine	151 (124–177)	16·0 (8·92–26·5)	167 (139–194)
Qatar	65·1 (52·6–79·5)	5·49 (3·26–8·56)	70·6 (58·0–85·2)
Saudi Arabia	691 (589–809)	65·6 (36·2–109)	757 (651–880)
Sudan	584 (475–716)	83·3 (43·3–146)	667 (552–813)
Syrian Arab Republic	452 (371–538)	112 (61·6–180)	564 (465–675)
Tunisia	303 (266–340)	21·6 (12·4–35·3)	324 (287–365)
Turkey	3120 (2890–3350)	1420 (1170–1700)	4540 (4190–4890)
United Arab Emirates	59·2 (48·5–71·0)	12·9 (7·79–20·7)	72·1 (60·2–86·4)
Yemen	573 (471–689)	155 (91·3–249)	727 (604–867)
**South Asia**	**24500 (21600–27700)**	**3210 (2090–4660)**	**27700 (24400–31400)**
Bangladesh	3720 (3230–4280)	178 (93·9–302)	3900 (3380–4480)
Bhutan	17·0 (14·0–20·1)	5·15 (3·25–7·57)	22·2 (18·5–26·0)
India	17300 (14400–20400)	2520 (1440–3970)	19800 (16700–23200)
Nepal	609 (523–700)	99·3 (57·3–164)	708 (609–818)
Pakistan	2800 (2270–3360)	408 (227–701)	3210 (2650–3810)

**Southeast Asia, East Asia, and Oceania**	**46300 (43500–49100)**	**3600 (2890–4680)**	**49800 (47000–52800)**
China	25000 (22300–27500)	1610 (994–2630)	26600 (23800–29400)
Democratic People's Republic of Korea	509 (419–605)	45·3 (22·5–79·3)	555 (459–653)
Taiwan (Province of China)	354 (293–415)	59·9 (30·7–105)	414 (345–490)
American Samoa	2·13 (1·75–2·52)	1·16 (0·677–1·79)	3·29 (2·68–3·98)
Cook Islands	0·454 (0·382–0·529)	0·483 (0·352–0·639)	0·937 (0·783–1·12)
Fiji	28·3 (23·8–33·0)	11·0 (7·09–16·5)	39·4 (33·0–46·9)
Guam	4·05 (3·55–4·58)	2·09 (1·57–2·68)	6·14 (5·40–6·95)
Kiribati	5·87 (5·33–6·39)	2·84 (2·13–3·64)	8·70 (7·82–9·70)
Marshall Islands	2·10 (1·81–2·38)	0·618 (0·406–0·883)	2·72 (2·36–3·11)
Micronesia (Federated States of)	6·78 (6·12–7·39)	3·86 (2·82–4·94)	10·6 (9·42–12·0)
Nauru	0·495 (0·430–0·556)	0·430 (0·326–0·546)	0·925 (0·805–1·07)
Niue	0·0256 (0·0208–0·0313)	0·0172 (0·0110–0·0257)	0·0428 (0·0345–0·0524)
Northern Mariana Islands	1·49 (1·24–1·74)	0·765 (0·516–1·09)	2·25 (1·90–2·67)
Palau	0·504 (0·448–0·562)	0·242 (0·199–0·291)	0·746 (0·675–0·817)
Papua New Guinea	460 (406–512)	206 (153–268)	665 (586–747)
Samoa	5·74 (4·97–6·56)	2·01 (1·36–2·81)	7·75 (6·71–8·86)
Solomon Islands	29·8 (27·0–32·7)	11·6 (8·91–14·8)	41·4 (37·6–45·4)
Tokelau	0·0424 (0·0343–0·0512)	0·0184 (0·00966–0·0290)	0·0608 (0·0482–0·0741)
Tonga	3·29 (2·85–3·76)	1·26 (0·863–1·76)	4·55 (3·95–5·21)
Tuvalu	0·474 (0·411–0·543)	0·191 (0·128–0·264)	0·666 (0·572–0·764)
Vanuatu	9·92 (8·60–11·3)	2·91 (1·86–4·12)	12·8 (11·1–14·6)
Cambodia	227 (187–273)	31·4 (17·6–52·6)	259 (216–308)
Indonesia	9420 (8720–10200)	491 (290–778)	9910 (9180–10700)
Lao People's Democratic Republic	201 (170–233)	24·0 (14·1–37·1)	225 (193–259)
Malaysia	969 (845–1090)	82·1 (49·5–130)	1050 (921–1180)
Maldives	14·6 (12·4–16·8)	1·67 (1·01–2·53)	16·3 (13·8–18·6)

Mauritius	39·3 (34·8–43·7)	8·41 (5·79–11·8)	47·7 (42·6–53·1)
Myanmar	1590 (1410–1770)	85·3 (45·4–141)	1670 (1490–1860)
Philippines	3540 (3170–3920)	687 (482–956)	4220 (3820–4680)
Seychelles	2·83 (2·32–3·31)	0·663 (0·438–0·935)	3·49 (2·94–4·03)
Sri Lanka	271 (225–322)	15·3 (7·79–28·3)	286 (240–340)
Thailand	1580 (1420–1740)	130 (79·9–208)	1710 (1530–1890)
Timor-Leste	77·8 (72·0–84·1)	5·50 (3·34–8·63)	83·3 (76·9–90·6)
Viet Nam	1930 (1690–2180)	75·9 (39·0–137)	2000 (1760–2260)
**Sub-Saharan Africa**	**10300 (9860–10800)**	**2350 (2040–2680)**	**12700 (12100–13200)**
Angola	212 (165–274)	34·1 (16·9–59·9)	247 (194–310)
Central African Republic	50·2 (37·3–65·4)	7·08 (3·49–13·6)	57·3 (43·9–73·3)
Congo	65·6 (50·7–82·9)	11·8 (5·79–21·0)	77·4 (60·8–96·8)
Democratic Republic of the Congo	915 (711–1160)	48·4 (25·3–86·6)	964 (755–1210)
Equatorial Guinea	45·6 (36·8–55·4)	6·97 (3·54–12·3)	52·6 (42·7–64·2)
Gabon	32·0 (26·3–38·2)	10·2 (6·06–16·3)	42·2 (35·0–50·8)
Burundi	65·6 (48·9–86·4)	13·5 (6·87–24·8)	79·1 (60·6–102)
Comoros	12·2 (9·90–14·7)	2·77 (1·63–4·42)	15·0 (12·3–18·0)
Djibouti	23·5 (19·1–28·3)	4·62 (2·70–7·63)	28·2 (23·2–33·7)
Eritrea	52·3 (38·4–70·6)	5·22 (2·62–9·83)	57·6 (43·3–75·9)
Ethiopia	488 (369–635)	82·8 (44·4–148)	571 (443–726)
Kenya	508 (402–624)	113 (63·1–182)	621 (494–752)
Madagascar	653 (548–769)	77·8 (42·7–130)	731 (616–859)
Malawi	173 (139–212)	39·2 (21·5–66·7)	212 (173–259)
Mozambique	283 (217–357)	119 (66·6–192)	402 (312–504)
Rwanda	84·9 (64·8–111)	10·3 (5·10–18·6)	95·2 (73·2–123)
Somalia	228 (170–306)	42·6 (21·9–77·1)	271 (204–352)
South Sudan	106 (77·9–136)	16·3 (8·10–28·7)	122 (92·7–155)

Uganda	335<br>(264–414)	149<br>(87·0–246)	484<br>(387–602)
United Republic of Tanzania	410<br>(323–517)	115<br>(63·4–196)	525<br>(420–656)
Zambia	302<br>(257–354)	140<br>(89·0–211)	442<br>(369–530)
Botswana	52·5<br>(44·5–61·1)	15·8<br>(10·5–22·7)	68·3<br>(58·2–79·3)
Eswatini	11·1<br>(8·68–14·0)	2·10<br>(1·07–3·61)	13·2<br>(10·6–16·4)
Lesotho	67·7<br>(58·6–76·7)	3·36<br>(1·65–6·25)	71·1<br>(62·2–80·5)
Namibia	39·6<br>(32·9–47·8)	19·8<br>(12·4–30·3)	59·4<br>(48·2–71·6)
South Africa	1300<br>(1160–1470)	576<br>(376–803)	1880<br>(1630–2150)
Zimbabwe	255<br>(213–304)	47·9<br>(25·8–79·8)	303<br>(255–360)
Benin	69·8<br>(53·1–90·0)	16·2<br>(8·93–27·6)	86·0<br>(65·3–107)
Burkina Faso	277<br>(217–342)	32·2<br>(16·7–57·8)	309<br>(247–378)
Cabo Verde	2·77<br>(2·00–3·64)	0·841<br>(0·438–1·45)	3·61<br>(2·72–4·64)
Cameroon	208<br>(161–265)	46·4<br>(25·3–75·7)	254<br>(201–316)
Chad	150<br>(117–190)	40·9<br>(21·1–73·2)	191<br>(149–242)
Côte d'Ivoire	492<br>(395–596)	87·4<br>(47·8–147)	580<br>(468–700)
Gambia	39·5<br>(32·6–47·0)	4·46<br>(2·40–7·57)	44·0<br>(37·0–51·8)
Ghana	129<br>(96·9–165)	39·7<br>(20·4–66·2)	168<br>(130–213)
Guinea	229<br>(179–284)	20·1<br>(9·76–36·5)	249<br>(199–305)
Guinea-Bissau	9·44<br>(6·80–12·8)	1·54<br>(0·780–2·82)	11·0<br>(8·11–14·6)
Liberia	23·6<br>(17·6–32·1)	15·2<br>(8·78–23·9)	38·9<br>(29·5–51·2)
Mali	354<br>(283–432)	23·5<br>(11·9–40·1)	378<br>(304–452)
Mauritania	105<br>(86·2–125)	41·1<br>(27·6–58·4)	146<br>(123–172)
Niger	307<br>(246–378)	50·7<br>(27·5–84·7)	357<br>(289–434)
Nigeria	767<br>(581–1010)	113<br>(56·2–209)	879<br>(685–1100)
São Tomé and Príncipe	0·544<br>(0·388–0·750)	0·117<br>(0·0572–0·211)	0·661<br>(0·485–0·884)
Senegal	182<br>(148–219)	23·6<br>(12·7–41·0)	205<br>(169–246)
Sierra Leone	145<br>(118–176)	65·7<br>(39·6–104)	211<br>(174–259)

Togo	56·4<br>(43·7–71·5)	10·5<br>(5·51–17·5)	66·9<br>(52·7–83·6)

Supplemental Table S4. Percent change in smoking prevalence, by sex, for ages 15-24, from 1990-2019.

Location	Percent Change Smoking Prevalence Ages 15-24 1990-2019 Male (%)	Percent Change Smoking Prevalence Ages 15-24 1990-2019 Female (%)	Percent Change Smoking Prevalence Ages 15-24 1990-2019 Both (%)
**Global**	**-32·9 (-35·9–-29·9)**	**-37·6 (-42·7–-32·2)**	**-33·6 (-36·2–-30·9)**
**Central Europe, Eastern Europe, and Central Asia**	**-35·3 (-38·6–-31·8)**	**-20·0 (-30·0–-8·05)**	**-30·7 (-34·2–-26·5)**
Armenia	-10·4 (-23·2–3·90)	20·2 (-55·8–152)	-7·01 (-20·6–8·15)
Azerbaijan	13·0 (-12·7–44·4)	47·5 (-40·3–208)	17·6 (-7·73–49·0)
Georgia	2·93 (-14·8–24·2)	29·7 (-37·2–133)	8·58 (-8·97–29·3)
Kazakhstan	-35·2 (-46·9–-22·3)	-11·3 (-56·0–51·2)	-32·1 (-44·2–-18·0)
Kyrgyzstan	-22·5 (-37·0–-5·59)	71·1 (-25·8–250)	-16·1 (-32·5–1·02)
Mongolia	10·1 (-11·2–35·9)	62·3 (-28·8–223)	14·7 (-6·65–41·2)
Tajikistan	-45·8 (-62·3–-25·2)	-30·7 (-73·5–49·9)	-43·7 (-59·5–-23·5)
Turkmenistan	-41·8 (-57·5–-23·1)	-32·8 (-74·8–51·9)	-39·5 (-55·1–-21·5)
Uzbekistan	45·7 (-4·10–118)	84·6 (-32·8–303)	50·0 (3·32–111)
Albania	65·0 (32·2–103)	104 (-3·18–280)	72·1 (38·6–113)
Bosnia and Herzegovina	34·2 (3·71–73·1)	72·0 (-1·09–173)	45·4 (11·1–86·9)
Bulgaria	-20·7 (-34·8–-4·84)	-8·37 (-37·1–29·4)	-15·6 (-31·7–2·61)
Croatia	-22·1 (-33·9–-9·09)	-14·0 (-35·3–14·5)	-18·8 (-30·6–-3·69)
Czechia	-18·3 (-31·4–-4·17)	13·2 (-13·1–43·6)	-7·02 (-18·8–8·45)
Hungary	-24·9 (-37·7–-9·40)	-7·50 (-36·0–28·4)	-18·1 (-31·6–-1·81)
Montenegro	-15·8 (-35·7–9·21)	-17·9 (-52·0–34·2)	-18·2 (-38·1–7·38)
North Macedonia	3·25 (-16·0–26·6)	-1·64 (-40·0–59·8)	0·238 (-19·8–26·6)
Poland	-40·4 (-47·5–-32·7)	-10·8 (-28·2–8·61)	-29·8 (-37·9–-21·4)
Romania	-27·3 (-37·5–-15·9)	23·6 (-8·74–64·7)	-13·5 (-24·2–-1·16)

Serbia	11·4 (-12·7–40·6)	73·9 (11·5–162)	36·3 (8·64–72·5)
Slovakia	-22·5 (-36·7–-6·60)	3·00 (-31·4–48·5)	-13·3 (-30·1–3·54)
Slovenia	-17·7 (-32·4–2·14)	2·57 (-29·5–49·0)	-10·1 (-26·4–8·88)
Belarus	-23·2 (-38·8–-5·46)	27·2 (-28·6–112)	-8·73 (-28·7–13·3)
Estonia	-31·4 (-41·7–-20·6)	-4·78 (-31·3–26·4)	-23·1 (-34·5–-11·5)
Latvia	-21·8 (-32·3–-11·2)	-4·77 (-33·3–33·6)	-16·3 (-28·2–-3·02)
Lithuania	-16·3 (-29·0–-1·04)	79·7 (27·0–149)	8·99 (-6·31–27·3)
Republic of Moldova	-1·50 (-20·7–21·7)	5·01 (-45·0–85·5)	0·0910 (-18·5–23·5)
Russian Federation	-37·3 (-44·6–-29·6)	-20·8 (-45·3–9·50)	-33·1 (-41·8–-23·6)
Ukraine	-36·1 (-44·6–-26·1)	-26·3 (-50·1–6·33)	-33·2 (-43·1–-21·7)
**High-income**	**-32·9 (-36·0–-29·8)**	**-36·0 (-41·3–-30·7)**	**-34·1 (-36·9–-31·1)**
Australia	-55·5 (-64·2–-45·3)	-60·0 (-72·7–-43·1)	-57·7 (-65·7–-47·4)
New Zealand	-27·2 (-36·2–-16·9)	-51·8 (-61·2–-40·8)	-40·2 (-46·8–-32·7)
Brunei Darussalam	-28·7 (-46·5–-8·80)	-2·33 (-57·4–97·2)	-24·9 (-42·5–-3·56)
Japan	-45·0 (-55·8–-33·1)	-30·9 (-61·4–14·8)	-42·5 (-53·7–-30·7)
Republic of Korea	-24·4 (-40·2–-6·46)	0·458 (-56·6–98·7)	-20·7 (-37·8–-1·59)
Singapore	-36·9 (-53·6–-13·4)	-38·7 (-70·3–11·3)	-38·8 (-55·6–-17·8)
Canada	-48·9 (-59·2–-37·8)	-54·1 (-69·8–-34·3)	-51·4 (-60·9–-40·5)
Greenland	-8·83 (-26·2–10·8)	-1·84 (-32·6–39·0)	-6·26 (-24·3–13·5)
United States of America	-32·1 (-40·3–-22·2)	-47·2 (-59·5–-34·0)	-39·2 (-46·0–-30·9)
Argentina	4·14 (-20·4–34·3)	-21·1 (-53·7–30·6)	-7·95 (-29·9–19·6)
Chile	-4·45 (-20·2–14·4)	-13·8 (-41·6–25·2)	-10·2 (-28·4–11·4)
Uruguay	-25·0 (-40·7–-5·92)	-35·9 (-59·0–-6·48)	-31·0 (-46·2–-13·4)
Andorra	-25·8 (-43·3–-4·57)	-25·7 (-60·0–26·9)	-26·5 (-44·6–-2·97)
Austria	-9·11 (-24·7–7·31)	-22·8 (-43·5–0·847)	-15·0 (-27·6–-0·927)
Belgium	-40·9 (-49·4–-31·4)	-32·9 (-48·1–-14·9)	-37·5 (-46·4–-29·1)
Cyprus	-1·15 (-20·2–21·2)	-15·5 (-56·2–47·2)	-7·29 (-27·7–15·7)

Denmark	-43·4 (-52·2–-34·1)	-48·5 (-60·3–-35·5)	-46·1 (-53·2–-38·0)
Finland	-29·1 (-40·0–-17·7)	-16·3 (-36·0–7·36)	-23·5 (-33·9–-11·7)
France	-26·9 (-35·2–-18·5)	-25·5 (-38·9–-11·5)	-26·2 (-33·8–-18·6)
Germany	-26·9 (-36·6–-17·0)	-29·2 (-44·7–-12·1)	-27·7 (-35·8–-18·6)
Greece	-15·7 (-26·3–-4·50)	-35·6 (-51·3–-16·6)	-24·8 (-34·6–-13·6)
Iceland	-55·3 (-64·3–-44·4)	-57·8 (-72·5–-36·2)	-56·8 (-65·4–-46·6)
Ireland	-39·4 (-50·1–-27·5)	-3·11 (-26·4–21·9)	-23·1 (-35·4–-10·1)
Israel	-32·8 (-44·6–-19·0)	-47·7 (-65·5–-23·8)	-38·8 (-49·7–-26·2)
Italy	-13·6 (-24·4–-2·29)	-18·4 (-35·5–-0·129)	-15·2 (-25·3–-4·58)
Luxembourg	-30·0 (-42·3–-14·9)	-8·86 (-28·7–12·1)	-20·3 (-31·0–-7·39)
Malta	-37·6 (-51·5–-20·9)	-19·3 (-48·1–18·1)	-30·1 (-43·9–-11·9)
Monaco	-27·3 (-46·5–-5·85)	-20·5 (-57·3–33·7)	-25·1 (-45·8–-1·14)
Netherlands	-36·1 (-45·6–-24·4)	-42·1 (-54·3–-27·8)	-39·1 (-46·7–-30·7)
Norway	-56·2 (-64·7–-46·0)	-64·7 (-75·6–-50·5)	-60·5 (-67·2–-52·1)
Portugal	-16·5 (-27·3–-4·88)	21·3 (-4·56–49·1)	-2·96 (-14·5–8·38)
San Marino	-39·7 (-53·2–-21·7)	-39·8 (-67·0–1·29)	-40·6 (-56·0–-21·4)
Spain	-38·3 (-46·7–-29·4)	-43·2 (-54·6–-30·5)	-40·7 (-47·5–-33·1)
Sweden	-36·9 (-51·3–-20·2)	-47·6 (-65·0–-26·7)	-43·2 (-54·9–-29·4)
Switzerland	-25·8 (-37·9–-12·8)	-35·4 (-53·4–-13·5)	-30·1 (-41·1–-18·1)
United Kingdom	-35·9 (-45·2–-25·5)	-43·8 (-57·0–-29·6)	-39·8 (-47·6–-31·7)
**Latin America and Caribbean**	**-50·2 (-54·3–-45·7)**	**-56·9 (-64·4–-48·2)**	**-52·1 (-56·1–-48·0)**
Bolivia (Plurinational State of)	1·68 (-26·2–38·5)	30·0 (-44·3–150)	7·99 (-22·0–45·5)
Ecuador	9·78 (-16·8–41·0)	29·2 (-38·6–143)	13·7 (-10·9–47·8)
Peru	-17·8 (-40·9–14·1)	-9·02 (-58·8–79·7)	-15·3 (-39·1–14·8)
Antigua and Barbuda	41·8 (-4·23–100)	81·3 (-20·7–248)	52·8 (4·76–118)
Bahamas	30·0 (-5·04–73·9)	18·6 (-45·2–134)	24·0 (-9·15–63·9)
Barbados	-4·60 (-32·6–34·9)	21·6 (-42·6–127)	2·00 (-27·9–41·5)

Belize	-0·644 (-29·0–34·5)	22·6 (-51·6–138)	1·51 (-24·9–33·4)
Bermuda	0·514 (-29·3–40·9)	2·57 (-56·1–107)	-2·10 (-32·0–35·6)
Cuba	-30·0 (-48·1–-6·14)	-31·2 (-63·4–18·8)	-31·5 (-49·8–-8·21)
Dominica	-0·515 (-32·2–39·2)	-10·1 (-59·8–71·3)	-6·38 (-35·1–35·9)
Dominican Republic	6·69 (-26·8–54·8)	17·0 (-47·1–127)	9·25 (-24·9–58·0)
Grenada	17·2 (-17·5–63·9)	13·8 (-48·9–119)	15·4 (-18·5–60·1)
Guyana	17·9 (-13·8–54·7)	4·09 (-55·8–105)	15·0 (-13·2–48·3)
Haiti	-41·6 (-61·0–-16·3)	-36·9 (-73·4–33·0)	-41·0 (-59·7–-18·5)
Jamaica	-11·5 (-35·7–18·3)	23·7 (-31·6–113)	-1·16 (-25·3–29·7)
Puerto Rico	-29·7 (-48·5–-8·07)	-22·7 (-59·4–35·3)	-28·4 (-46·9–-6·74)
Saint Kitts and Nevis	2·13 (-31·9–53·7)	9·47 (-54·1–134)	0·995 (-30·7–40·2)
Saint Lucia	-12·8 (-37·6–19·3)	13·9 (-46·3–107)	-6·17 (-31·6–26·4)
Saint Vincent and the Grenadines	9·38 (-18·8–40·9)	4·00 (-50·9–89·2)	7·39 (-19·1–36·8)
Suriname	-16·9 (-35·0–6·26)	-11·9 (-56·5–66·2)	-18·1 (-36·1–2·92)
Trinidad and Tobago	-9·48 (-31·3–16·7)	0·386 (-47·7–88·4)	-8·21 (-29·7–18·7)
United States Virgin Islands	-17·9 (-46·0–24·3)	2·17 (-64·6–142)	-14·1 (-44·7–27·2)
Colombia	-56·3 (-66·8–-43·9)	-54·2 (-74·7–-22·6)	-55·5 (-66·2–-42·2)
Costa Rica	-44·7 (-58·3–-27·2)	-52·4 (-73·9–-18·6)	-48·5 (-61·1–-31·7)
El Salvador	29·6 (-8·05–75·5)	117 (-1·73–332)	42·7 (4·87–91·4)
Guatemala	-11·7 (-32·9–15·5)	80·7 (-15·5–233)	2·39 (-22·1–32·2)
Honduras	-21·4 (-40·1–0·435)	43·4 (-27·0–143)	-12·7 (-31·6–10·1)
Mexico	-39·7 (-48·9–-29·6)	-39·1 (-61·3–-8·08)	-39·3 (-48·9–-28·5)
Nicaragua	-29·5 (-45·6–-9·05)	2·70 (-53·0–89·5)	-22·2 (-39·9–0·319)
Panama	-38·7 (-56·4–-17·0)	-41·0 (-71·8–12·5)	-40·4 (-57·6–-18·6)
Venezuela (Bolivarian Republic of)	-35·0 (-51·7–-13·1)	-34·8 (-68·3–19·9)	-36·1 (-52·6–-15·8)
Brazil	-73·6 (-78·7–-67·4)	-75·9 (-84·2–-65·5)	-74·5 (-78·9–-69·0)
Paraguay	-45·1 (-59·4–-26·5)	-33·4 (-67·9–27·9)	-42·2 (-57·8–-22·5)

**North Africa and Middle East**	**-4·07 (-11·2–3·57)**	**8·72 (-11·5–32·9)**	**-1·50 (-8·16–6·01)**
Afghanistan	247 (137–378)	199 (23·2–525)	241 (143–362)
Algeria	1·37 (-23·9–31·6)	-13·7 (-67·2–75·9)	0·415 (-23·2–28·9)
Bahrain	39·6 (8·01–76·3)	70·6 (-11·7–190)	42·6 (12·0–76·5)
Egypt	18·5 (-6·69–46·5)	27·4 (-53·8–190)	17·6 (-6·79–45·7)
Iran (Islamic Republic of)	19·0 (-15·4–65·8)	34·8 (-46·3–162)	19·8 (-12·3–60·5)
Iraq	-6·66 (-28·0–20·0)	19·9 (-55·2–163)	-4·98 (-26·0–22·7)
Jordan	15·5 (-3·32–38·7)	48·6 (-18·9–143)	20·0 (-0·131–44·1)
Kuwait	-4·09 (-21·6–19·2)	51·3 (-25·3–186)	0·817 (-17·2–24·3)
Lebanon	34·8 (6·70–70·4)	30·7 (-28·3–121)	32·8 (6·20–66·8)
Libya	1·99 (-24·3–38·7)	15·6 (-52·8–150)	0·787 (-23·8–34·6)
Morocco	-32·8 (-49·0–-10·6)	1·43 (-60·7–116)	-29·7 (-46·6–-8·90)
Oman	-6·49 (-33·8–28·2)	30·4 (-48·7–164)	-1·67 (-27·9–34·1)
Palestine	1·74 (-20·7–25·9)	85·5 (-22·4–264)	5·37 (-16·4–28·2)
Qatar	8·98 (-22·8–47·7)	19·9 (-53·3–144)	22·7 (-10·4–67·0)
Saudi Arabia	44·2 (13·8–80·9)	79·4 (-27·0–273)	47·5 (17·4–83·5)
Sudan	-12·7 (-37·4–20·5)	10·3 (-56·2–141)	-8·19 (-32·0–24·4)
Syrian Arab Republic	-17·4 (-36·0–5·14)	-16·2 (-64·8–79·2)	-21·4 (-39·7–-1·12)
Tunisia	-13·2 (-28·6–3·82)	-14·2 (-64·3–85·6)	-14·0 (-29·0–3·03)
Turkey	-7·88 (-21·3–6·92)	39·3 (1·40–93·7)	3·26 (-10·3–19·6)
United Arab Emirates	-4·05 (-32·3–32·0)	65·0 (-29·5–234)	-6·99 (-32·6–23·4)
Yemen	4·76 (-23·9–39·8)	26·6 (-48·0–156)	6·95 (-21·5–39·7)
**South Asia**	**-21·1 (-32·7–-7·96)**	**-23·9 (-58·6–35·7)**	**-22·1 (-33·7–-9·12)**
Bangladesh	-18·7 (-33·7–-0·956)	12·4 (-56·5–134)	-19·7 (-34·3–-2·46)
Bhutan	-2·43 (-28·0–30·0)	32·3 (-45·2–159)	-0·671 (-26·2–33·2)
India	-16·0 (-33·1–3·64)	-22·7 (-63·7–56·2)	-17·7 (-34·1–1·15)
Nepal	-14·4 (-34·1–7·75)	-55·2 (-80·5–-11·2)	-25·6 (-41·9–-7·04)

Pakistan	-40·0 (-57·3–-20·2)	-16·6 (-68·8–73·7)	-39·6 (-55·6–-21·1)
**Southeast Asia, East Asia, and Oceania**	**-13·5 (-20·0–-5·99)**	**49·1 (-2·87–119)**	**-8·94 (-15·4–-1·55)**
China	-14·8 (-25·2–-3·56)	74·0 (-22·4–252)	-9·85 (-21·3–1·66)
Democratic People's Republic of Korea	-4·73 (-27·8–24·0)	31·4 (-49·0–190)	8·02 (-20·6–39·9)
Taiwan (Province of China)	-16·3 (-36·8–9·17)	-7·83 (-70·5–115)	-15·9 (-36·7–9·17)
American Samoa	-4·59 (-25·6–20·2)	-1·57 (-52·1–75·9)	-4·71 (-28·6–23·9)
Cook Islands	-9·66 (-27·8–13·0)	-3·11 (-39·9–51·8)	-7·74 (-28·4–17·5)
Fiji	-7·96 (-27·1–13·1)	-10·3 (-53·9–58·5)	-9·42 (-28·4–12·1)
Guam	-19·9 (-37·4–2·25)	-30·4 (-58·0–12·3)	-25·6 (-40·8–-6·92)
Kiribati	4·38 (-11·1–22·0)	11·9 (-35·4–84·9)	5·19 (-14·2–26·5)
Marshall Islands	4·10 (-16·5–29·0)	70·9 (-21·4–209)	13·3 (-9·77–37·6)
Micronesia (Federated States of)	7·87 (-7·72–25·1)	21·4 (-26·2–91·0)	12·2 (-7·81–34·9)
Nauru	-4·51 (-22·8–17·2)	-8·01 (-41·3–35·4)	-6·82 (-26·8–16·1)
Niue	-10·8 (-34·7–19·5)	5·83 (-50·6–106)	-7·64 (-33·9–26·5)
Northern Mariana Islands	-20·5 (-37·3–-0·633)	-33·7 (-60·5–5·95)	-24·5 (-40·7–-3·55)
Palau	6·13 (-14·4–32·2)	32·6 (-21·6–121)	14·1 (-9·05–39·8)
Papua New Guinea	-15·3 (-30·1–1·03)	-9·92 (-47·0–47·3)	-14·2 (-30·1–5·30)
Samoa	-1·36 (-24·0–28·4)	-16·1 (-57·9–62·1)	-9·04 (-32·1–18·1)
Solomon Islands	0·990 (-16·3–21·6)	1·11 (-43·2–75·3)	-0·0427 (-18·8–22·7)
Tokelau	-3·09 (-26·8–24·9)	3·66 (-50·8–86·2)	-3·96 (-28·4–23·6)
Tonga	-15·7 (-30·4–2·07)	19·8 (-37·7–100)	-9·90 (-27·4–8·30)
Tuvalu	16·2 (-7·90–48·0)	41·2 (-31·4–187)	23·9 (-3·97–58·7)
Vanuatu	-23·2 (-37·6–-7·60)	-14·4 (-57·5–54·2)	-22·8 (-37·7–-5·65)
Cambodia	-2·29 (-26·9–29·9)	58·3 (-36·3–240)	6·09 (-20·8–39·1)
Indonesia	-6·81 (-17·2–4·46)	63·8 (-32·5–240)	-2·03 (-12·9–9·88)
Lao People's Democratic Republic	13·0 (-13·9–45·3)	53·4 (-37·8–210)	20·4 (-6·75–53·7)
Malaysia	-28·6 (-41·5–-14·1)	-11·3 (-61·4–77·1)	-26·2 (-39·3–-11·9)

Maldives	11·9<br>(-12·6–40·7)	15·8<br>(-52·7–129)	24·4<br>(-2·87–55·0)
Mauritius	-8·28<br>(-23·1–9·34)	23·2<br>(-40·5–124)	-5·55<br>(-20·9–13·4)
Myanmar	-26·5<br>(-39·7–-10·8)	-33·1<br>(-73·8–47·8)	-27·5<br>(-39·9–-13·1)
Philippines	-31·4<br>(-41·8–-18·9)	-8·75<br>(-52·5–62·4)	-27·9<br>(-38·7–-15·5)
Seychelles	-8·58<br>(-28·7–15·9)	-11·0<br>(-54·8–60·6)	-7·27<br>(-27·9–16·6)
Sri Lanka	-22·1<br>(-42·3–2·33)	-42·1<br>(-77·4–28·7)	-24·5<br>(-43·7–-1·00)
Thailand	-16·2<br>(-29·0–-2·21)	-4·60<br>(-55·0–87·0)	-15·7<br>(-28·5–-1·55)
Timor-Leste	-10·5<br>(-22·7–2·78)	75·7<br>(-25·5–271)	-10·5<br>(-22·5–3·66)
Viet Nam	-25·4<br>(-37·8–-10·7)	19·2<br>(-56·1–169)	-21·2<br>(-33·7–-6·48)
**Sub-Saharan Africa**	**-29·0**<br>**(-33·9–-24·2)**	**-19·8**<br>**(-34·0–-3·38)**	**-26·8**<br>**(-31·7–-21·8)**
Angola	-18·3<br>(-45·2–17·0)	-6·18<br>(-63·9–97·1)	-20·2<br>(-44·5–9·63)
Central African Republic	-30·3<br>(-52·0–0·360)	-15·1<br>(-67·4–79·5)	-29·6<br>(-50·6–-2·27)
Congo	20·6<br>(-16·9–68·2)	67·6<br>(-44·4–279)	23·5<br>(-13·1–67·5)
Democratic Republic of the Congo	-11·0<br>(-40·0–27·2)	10·1<br>(-58·1–145)	-10·3<br>(-38·5–26·4)
Equatorial Guinea	4·24<br>(-25·2–41·7)	16·9<br>(-56·8–165)	19·5<br>(-14·4–60·7)
Gabon	26·0<br>(-10·1–75·7)	54·5<br>(-35·8–224)	26·8<br>(-7·24–71·6)
Burundi	-52·5<br>(-67·9–-29·8)	-40·6<br>(-77·2–35·8)	-52·0<br>(-66·6–-29·0)
Comoros	-3·28<br>(-30·7–31·0)	12·6<br>(-56·7–148)	-1·72<br>(-27·7–31·1)
Djibouti	10·5<br>(-22·1–49·0)	34·4<br>(-42·6–159)	11·7<br>(-18·3–48·0)
Eritrea	-20·9<br>(-48·5–14·1)	-14·4<br>(-67·0–82·0)	-21·6<br>(-48·0–11·5)
Ethiopia	0·796<br>(-35·2–49·5)	6·59<br>(-59·5–136)	1·67<br>(-31·5–44·9)
Kenya	-43·4<br>(-60·0–-22·5)	-28·6<br>(-70·3–45·4)	-40·8<br>(-57·6–-20·7)
Madagascar	-36·0<br>(-50·1–-18·9)	-12·9<br>(-65·9–91·9)	-33·7<br>(-48·0–-16·0)
Malawi	5·84<br>(-26·5–47·8)	73·9<br>(-38·5–281)	13·0<br>(-18·8–54·6)
Mozambique	-8·78<br>(-37·5–28·1)	31·9<br>(-47·8–192)	-0·346<br>(-32·9–42·2)
Rwanda	0·00427<br>(-37·0–45·6)	4·83<br>(-61·2–122)	-0·0419<br>(-33·9–44·1)
Somalia	-11·5<br>(-40·6–26·0)	-16·1<br>(-67·1–79·6)	-14·5<br>(-40·6–20·0)

South Sudan	-20·5 (-46·3–12·5)	-1·63 (-60·6–123)	-22·3 (-47·1–8·76)
Uganda	-19·7 (-43·6–10·8)	65·3 (-30·7–238)	-4·62 (-31·3–30·5)
United Republic of Tanzania	-38·0 (-56·9–-11·6)	-6·67 (-61·2–86·5)	-33·5 (-51·6–-10·2)
Zambia	27·4 (-7·14–71·2)	129 (-0·161–356)	46·7 (8·03–98·5)
Botswana	0·259 (-24·4–30·7)	7·00 (-48·9–105)	3·04 (-22·9–35·4)
Eswatini	-33·7 (-52·1–-11·1)	-16·8 (-67·7–71·4)	-29·2 (-47·8–-7·19)
Lesotho	37·7 (5·01–79·7)	11·2 (-62·3–169)	38·7 (7·80–78·4)
Namibia	-14·4 (-37·0–15·2)	6·30 (-51·3–113)	-9·74 (-35·6–19·7)
South Africa	-10·5 (-25·2–6·59)	5·84 (-38·6–71·4)	-4·48 (-22·1–16·3)
Zimbabwe	6·71 (-21·3–43·6)	200 (13·8–541)	17·8 (-13·1–55·5)
Benin	-45·0 (-63·7–-21·9)	-26·3 (-71·7–50·1)	-41·4 (-59·8–-17·9)
Burkina Faso	-29·4 (-49·4–-2·65)	-20·4 (-69·0–73·4)	-27·7 (-47·2–-2·18)
Cabo Verde	-46·3 (-65·3–-22·4)	-28·0 (-71·8–56·2)	-43·1 (-60·3–-20·0)
Cameroon	-25·6 (-49·1–7·45)	20·4 (-50·7–140)	-18·8 (-42·9–13·1)
Chad	-29·7 (-50·1–2·56)	-23·5 (-71·8–67·4)	-29·2 (-49·4–-1·29)
Côte d'Ivoire	11·3 (-21·1–51·3)	25·9 (-49·0–164)	15·0 (-16·4–52·1)
Gambia	-27·8 (-47·1–-5·33)	7·96 (-58·3–144)	-25·6 (-44·4–-3·78)
Ghana	9·55 (-28·8–63·9)	20·8 (-56·6–159)	9·61 (-23·7–60·6)
Guinea	-10·7 (-35·8–19·5)	11·8 (-56·9–138)	-11·1 (-34·9–19·2)
Guinea-Bissau	17·7 (-23·6–75·7)	17·8 (-56·7–148)	17·5 (-20·6–73·5)
Liberia	-0·627 (-34·4–45·7)	82·7 (-29·4–265)	22·7 (-17·7–75·7)
Mali	27·9 (-8·05–76·6)	60·3 (-36·7–239)	31·8 (-4·03–79·4)
Mauritania	-33·7 (-48·1–-14·8)	-14·0 (-53·5–49·4)	-30·5 (-45·3–-12·7)
Niger	-3·93 (-33·6–34·5)	18·5 (-51·6–150)	-0·403 (-29·1–35·9)
Nigeria	-56·8 (-69·3–-39·9)	-39·9 (-77·5–21·3)	-55·5 (-68·2–-40·4)
São Tomé and Príncipe	31·2 (-18·1–96·3)	21·5 (-54·0–174)	26·2 (-16·4–79·8)
Senegal	-58·4 (-68·8–-44·6)	-33·5 (-73·1–44·7)	-54·2 (-65·2–-40·0)

Sierra Leone	-20·1<br>(-41·8–6·58)	-16·1<br>(-61·2–66·3)	-19·4<br>(-40·2–9·17)
Togo	-46·1<br>(-62·9–-24·3)	-31·7<br>(-74·2–44·1)	-43·4<br>(-60·5–-22·2)

Supplemental Table S5. Percent change in number of smokers, by sex, for ages 15-24, from 1990-2019.

Location	Percent Change Number of Smokers Ages 15-24 1990-2019 Male (%)	Percent Change Number of Smokers Ages 15-24 1990-2019 Female (%)	Percent Change Number of Smokers Ages 15-24 1990-2019 Both (%)
**Global**	**-18·6 (-22·1–-14·9)**	**-25·4 (-31·5–-18·9)**	**-20·0 (-23·2–-16·7)**
**Central Europe, Eastern Europe, and Central Asia**	**-50·1 (-52·7–-47·4)**	**-39·4 (-47·0–-30·4)**	**-47·0 (-49·7–-43·9)**
Armenia	-41·7 (-50·0–-32·5)	-26·9 (-73·1–53·4)	-41·5 (-50·0–-31·9)
Azerbaijan	21·6 (-6·00–55·5)	48·2 (-40·0–209)	22·4 (-3·96–55·1)
Georgia	-47·9 (-56·9–-37·1)	-40·1 (-71·0–7·40)	-47·4 (-55·9–-37·4)
Kazakhstan	-43·5 (-53·7–-32·3)	-20·9 (-60·8–34·8)	-40·1 (-50·9–-27·8)
Kyrgyzstan	5·45 (-14·3–28·4)	131 (0·0569–371)	13·6 (-8·55–36·8)
Mongolia	17·2 (-5·42–44·7)	69·4 (-25·7–237)	20·9 (-1·59–48·9)
Tajikistan	-5·76 (-34·4–30·1)	13·4 (-56·6–145)	-5·04 (-31·7–29·1)
Turkmenistan	-31·8 (-50·2–-9·84)	-28·2 (-73·1–62·3)	-32·2 (-49·6–-11·9)
Uzbekistan	116 (42·2–223)	163 (-4·36–474)	118 (50·1–207)
Albania	8·74 (-12·9–33·8)	27·8 (-39·3–138)	10·6 (-10·9–37·0)
Bosnia and Herzegovina	-33·4 (-48·5–-14·1)	-11·8 (-49·3–40·1)	-26·7 (-44·0–-5·78)
Bulgaria	-58·7 (-66·0–-50·4)	-53·3 (-67·9–-34·1)	-56·5 (-64·8–-47·1)
Croatia	-47·2 (-55·2–-38·4)	-42·3 (-56·6–-23·2)	-45·2 (-53·2–-35·1)
Czechia	-48·9 (-57·1–-40·0)	-29·9 (-46·1–-11·0)	-42·1 (-49·4–-32·5)
Hungary	-47·9 (-56·8–-37·2)	-36·4 (-56·0–-11·8)	-43·4 (-52·8–-32·2)
Montenegro	-34·0 (-49·6–-14·4)	-35·7 (-62·4–5·02)	-35·9 (-51·5–-15·9)
North Macedonia	-18·4 (-33·7–0·0448)	-24·1 (-53·7–23·3)	-21·7 (-37·3–-1·16)
Poland	-56·3 (-61·5–-50·7)	-35·0 (-47·7–-20·9)	-48·7 (-54·6–-42·6)
Romania	-61·5 (-66·9–-55·4)	-36·4 (-53·0–-15·2)	-54·8 (-60·4–-48·4)

Serbia	-10·9 (-30·1–12·6)	34·9 (-13·5–103)	7·41 (-14·4–36·0)
Slovakia	-45·2 (-55·2–-34·0)	-28·3 (-52·2–3·37)	-39·1 (-50·9–-27·3)
Slovenia	-45·3 (-55·1–-32·2)	-34·4 (-54·9–-4·75)	-41·4 (-52·0–-29·0)
Belarus	-50·6 (-60·6–-39·2)	-23·1 (-56·9–27·8)	-43·1 (-55·5–-29·3)
Estonia	-60·5 (-66·5–-54·3)	-44·0 (-59·6–-25·7)	-55·3 (-61·9–-48·5)
Latvia	-62·8 (-67·8–-57·8)	-54·8 (-68·3–-36·5)	-60·2 (-65·9–-53·9)
Lithuania	-54·2 (-61·2–-45·9)	-2·77 (-31·3–34·9)	-40·7 (-49·0–-30·7)
Republic of Moldova	-36·7 (-49·1–-21·8)	-35·5 (-66·2–14·0)	-37·1 (-48·7–-22·4)
Russian Federation	-55·4 (-60·6–-49·9)	-44·2 (-61·5–-22·8)	-52·6 (-58·8–-45·9)
Ukraine	-61·1 (-66·3–-55·1)	-56·3 (-70·4–-36·9)	-59·9 (-65·8–-53·0)
**High-income**	**-38·1 (-41·0–-35·2)**	**-41·9 (-46·7–-37·0)**	**-39·7 (-42·2–-36·9)**
Australia	-50·3 (-60·1–-39·0)	-55·8 (-69·8–-37·1)	-53·0 (-61·9–-41·5)
New Zealand	-26·3 (-35·4–-15·9)	-51·5 (-61·0–-40·5)	-39·7 (-46·4–-32·1)
Brunei Darussalam	10·3 (-17·1–41·2)	42·3 (-37·9–187)	13·0 (-13·4–45·0)
Japan	-65·2 (-72·0–-57·7)	-56·8 (-75·9–-28·2)	-63·8 (-70·9–-56·5)
Republic of Korea	-48·6 (-59·4–-36·4)	-34·0 (-71·5–30·5)	-47·0 (-58·4–-34·2)
Singapore	-44·5 (-59·3–-23·9)	-44·6 (-73·1–0·672)	-45·4 (-60·4–-26·7)
Canada	-44·7 (-55·8–-32·6)	-51·1 (-67·9–-30·1)	-47·9 (-58·0–-36·1)
Greenland	-32·4 (-45·2–-17·8)	-19·5 (-44·7–14·0)	-27·0 (-41·1–-11·6)
United States of America	-21·9 (-31·3–-10·5)	-39·2 (-53·4–-24·1)	-30·0 (-37·9–-20·6)
Argentina	39·4 (6·59–79·9)	3·11 (-39·5–70·8)	21·8 (-7·26–58·3)
Chile	-0·539 (-17·0–19·1)	-14·0 (-41·7–24·9)	-8·42 (-27·1–13·5)
Uruguay	-22·5 (-38·7–-2·70)	-34·2 (-57·8–-3·94)	-28·9 (-44·6–-10·7)
Andorra	-26·8 (-44·1–-5·87)	-24·6 (-59·4–28·7)	-26·5 (-44·6–-3·02)
Austria	-22·8 (-36·0–-8·83)	-36·9 (-53·9–-17·7)	-29·2 (-39·6–-17·4)
Belgium	-45·9 (-53·8–-37·3)	-38·3 (-52·2–-21·7)	-42·7 (-50·8–-35·0)
Cyprus	17·2 (-5·37–43·6)	1·15 (-47·5–76·3)	10·4 (-13·9–37·8)

Denmark	-46·6 (-54·8–-37·7)	-51·2 (-62·4–-38·9)	-49·0 (-55·7–-41·3)
Finland	-33·0 (-43·3–-22·2)	-21·8 (-40·2–0·355)	-28·1 (-37·8–-17·0)
France	-33·6 (-41·2–-26·0)	-33·0 (-45·1–-20·5)	-33·4 (-40·2–-26·5)
Germany	-36·9 (-45·3–-28·4)	-43·1 (-55·5–-29·3)	-39·7 (-46·4–-32·1)
Greece	-45·5 (-52·3–-38·3)	-58·3 (-68·4–-46·0)	-51·3 (-57·7–-44·1)
Iceland	-52·1 (-61·8–-40·4)	-54·9 (-70·6–-31·7)	-53·7 (-62·9–-42·8)
Ireland	-40·9 (-51·2–-29·2)	-4·23 (-27·3–20·5)	-24·5 (-36·6–-11·7)
Israel	8·00 (-10·9–30·3)	-15·7 (-44·5–22·7)	-1·51 (-19·1–18·7)
Italy	-42·6 (-49·8–-35·1)	-48·0 (-58·9–-36·3)	-44·8 (-51·4–-37·9)
Luxembourg	-0·756 (-18·2–20·6)	26·7 (-0·815–55·9)	11·9 (-3·12–30·0)
Malta	-45·5 (-57·6–-30·8)	-29·0 (-54·4–3·90)	-38·7 (-50·8–-22·7)
Monaco	-16·5 (-38·6–8·15)	-11·4 (-52·4–49·1)	-15·2 (-38·7–11·9)
Netherlands	-43·8 (-52·1–-33·5)	-49·1 (-59·8–-36·5)	-46·4 (-53·1–-39·1)
Norway	-55·1 (-63·9–-44·7)	-64·5 (-75·4–-50·2)	-59·9 (-66·7–-51·3)
Portugal	-41·8 (-49·3–-33·7)	-16·7 (-34·4–2·46)	-32·8 (-40·8–-25·0)
San Marino	-36·1 (-50·4–-16·9)	-36·9 (-65·4–6·21)	-37·3 (-53·6–-17·1)
Spain	-57·7 (-63·5–-51·6)	-61·4 (-69·1–-52·8)	-59·5 (-64·2–-54·3)
Sweden	-37·9 (-52·0–-21·4)	-50·0 (-66·6–-30·1)	-44·9 (-56·3–-31·5)
Switzerland	-27·5 (-39·3–-14·8)	-37·5 (-54·9–-16·3)	-32·0 (-42·7–-20·4)
United Kingdom	-39·4 (-48·1–-29·6)	-48·3 (-60·4–-35·2)	-43·8 (-51·1–-36·2)
**Latin America and Caribbean**	**-36·8 (-42·0–-31·0)**	**-47·8 (-56·9–-37·3)**	**-40·6 (-45·5–-35·5)**
Bolivia (Plurinational State of)	83·5 (33·1–150)	123 (-4·32–330)	90·1 (37·4–156)
Ecuador	81·0 (37·2–133)	101 (-4·56–279)	82·1 (42·7–137)
Peru	12·5 (-19·1–56·2)	11·4 (-49·6–120)	9·72 (-21·1–48·8)
Antigua and Barbuda	78·4 (20·5–152)	117 (-5·29–315)	87·3 (28·4–167)
Bahamas	49·0 (8·77–99·2)	36·4 (-36·9–169)	42·4 (4·29–88·2)
Barbados	-18·2 (-42·2–15·6)	3·47 (-51·1–93·2)	-12·9 (-38·4–20·9)

Belize	118 (56·0–196)	175 (8·39–433)	125 (66·6–196)
Bermuda	-30·8 (-51·3–-3·00)	-26·6 (-68·6–47·9)	-31·3 (-52·3–-4·84)
Cuba	-56·9 (-68·0–-42·2)	-58·7 (-78·0–-28·6)	-58·3 (-69·5–-44·1)
Dominica	-24·9 (-48·8–5·08)	-32·6 (-69·8–28·6)	-29·5 (-51·1–2·32)
Dominican Republic	40·6 (-3·50–104)	41·3 (-36·1–174)	37·7 (-5·33–99·1)
Grenada	36·9 (-3·67–91·4)	22·9 (-44·8–136)	29·8 (-8·31–80·0)
Guyana	11·6 (-18·3–46·4)	-6·85 (-60·4–83·7)	5·81 (-20·1–36·5)
Haiti	19·8 (-20·0–71·8)	22·8 (-48·2–159)	17·8 (-19·5–62·6)
Jamaica	-4·09 (-30·3–28·3)	25·3 (-30·7–116)	3·56 (-21·7–35·9)
Puerto Rico	-48·4 (-62·2–-32·5)	-44·0 (-70·6–-1·99)	-47·7 (-61·2–-32·0)
Saint Kitts and Nevis	10·9 (-26·0–66·9)	22·0 (-48·9–161)	11·1 (-23·8–54·3)
Saint Lucia	-14·4 (-38·8–17·1)	6·17 (-50·0–93·4)	-10·3 (-34·5–20·9)
Saint Vincent and the Grenadines	-13·6 (-35·9–11·3)	-20·5 (-62·5–44·6)	-16·5 (-37·2–6·33)
Suriname	-6·78 (-27·1–19·2)	4·62 (-48·3–97·3)	-5·62 (-26·3–18·7)
Trinidad and Tobago	-25·2 (-43·3–-3·58)	-18·3 (-57·5–53·4)	-24·7 (-42·3–-2·70)
United States Virgin Islands	-41·5 (-61·5–-11·4)	-29·4 (-75·5–67·4)	-39·7 (-61·2–-10·7)
Colombia	-44·0 (-57·5–-28·2)	-46·3 (-70·4–-9·21)	-45·5 (-58·6–-29·3)
Costa Rica	-28·4 (-46·0–-5·69)	-37·6 (-65·8–6·68)	-32·8 (-49·3–-11·1)
El Salvador	46·2 (3·74–98·0)	134 (6·18–367)	57·5 (15·7–111)
Guatemala	135 (78·3–207)	343 (107–717)	161 (98·9–237)
Honduras	84·0 (40·2–135)	229 (67·4–458)	102 (58·4–155)
Mexico	-27·2 (-38·3–-14·9)	-29·7 (-55·4–5·99)	-28·3 (-39·7–-15·6)
Nicaragua	18·1 (-8·85–52·5)	57·3 (-28·0–190)	24·6 (-3·79–60·7)
Panama	-10·3 (-36·2–21·4)	-16·1 (-59·9–60·0)	-14·0 (-38·8–17·5)
Venezuela (Bolivarian Republic of)	-26·6 (-45·4–-1·72)	-25·1 (-63·6–37·8)	-27·1 (-46·0–-4·04)
Brazil	-68·8 (-74·9–-61·5)	-72·6 (-82·0–-60·7)	-70·4 (-75·5–-64·1)
Paraguay	-3·47 (-28·6–29·1)	11·8 (-46·1–115)	-0·755 (-27·5–33·2)

**North Africa and Middle East**	**49·0 (37·9–60·9)**	**65·3 (34·6–102)**	**51·4 (41·2–63·0)**
Afghanistan	1110 (724–1560)	876 (302–1940)	1050 (720–1460)
Algeria	21·7 (-8·64–58·0)	1·88 (-61·3–108)	19·6 (-8·55–53·5)
Bahrain	172 (110–243)	239 (75·7–476)	181 (120–247)
Egypt	98·8 (56·5–146)	116 (-21·6–392)	98·3 (57·2–146)
Iran (Islamic Republic of)	15·4 (-17·9–60·9)	29·8 (-48·3–153)	15·8 (-15·2–55·2)
Iraq	140 (85·0–208)	204 (13·5–567)	143 (89·0–213)
Jordan	200 (151–260)	292 (114–540)	214 (161–277)
Kuwait	65·0 (34·8–105)	161 (29·1–393)	73·8 (42·7–114)
Lebanon	60·6 (27·1–103)	50·7 (-17·4–154)	55·8 (24·6–95·6)
Libya	36·4 (1·28–85·5)	59·7 (-34·9–245)	36·9 (3·45–82·8)
Morocco	-18·1 (-37·8–8·90)	18·6 (-54·0–152)	-16·1 (-36·2–8·74)
Oman	106 (45·9–182)	163 (3·41–432)	109 (53·2–185)
Palestine	149 (93·9–208)	358 (91·7–799)	159 (105–215)
Qatar	695 (463–978)	349 (74·8–813)	638 (439–905)
Saudi Arabia	171 (114–240)	225 (32·2–576)	173 (117–239)
Sudan	95·0 (39·7–169)	125 (-10·6–392)	96·1 (45·3–166)
Syrian Arab Republic	-4·59 (-26·1–21·4)	9·33 (-54·0–134)	-3·43 (-26·0–21·5)
Tunisia	-17·5 (-32·1–-1·25)	-18·2 (-66·0–76·9)	-18·1 (-32·4–-1·90)
Turkey	3·70 (-11·4–20·4)	52·4 (11·0–112)	14·6 (-0·448–32·8)
United Arab Emirates	102 (42·7–178)	366 (99·1–844)	123 (61·7–196)
Yemen	176 (101–268)	235 (37·6–578)	182 (107–269)
**South Asia**	**30·7 (11·6–52·5)**	**27·4 (-30·6–127)**	**29·7 (10·4–51·3)**
Bangladesh	9·67 (-10·6–33·6)	58·4 (-38·6–230)	10·9 (-9·26–34·6)
Bhutan	-0·499 (-26·6–32·5)	48·6 (-38·4–191)	5·97 (-21·3–42·1)
India	37·5 (9·61–69·7)	26·8 (-40·4–156)	34·9 (8·03–65·8)
Nepal	50·5 (15·9–89·4)	-19·9 (-65·2–58·6)	31·9 (2·96–64·8)

Pakistan	25·0 (-10·9–66·2)	82·9 (-31·7–281)	28·9 (-5·09–68·6)
**Southeast Asia, East Asia, and Oceania**	**-31·8 (-36·9–-25·9)**	**11·9 (-27·1–64·3)**	**-29·9 (-34·9–-24·2)**
China	-46·8 (-53·3–-39·8)	2·05 (-54·5–106)	-45·4 (-52·4–-38·4)
Democratic People's Republic of Korea	10·8 (-16·0–44·2)	19·4 (-53·6–164)	10·5 (-18·7–43·1)
Taiwan (Province of China)	-36·5 (-52·0–-17·1)	-32·3 (-78·3–57·8)	-37·1 (-52·7–-18·4)
American Samoa	10·1 (-14·1–38·7)	11·3 (-45·8–99·0)	8·87 (-18·5–41·5)
Cook Islands	-40·4 (-52·4–-25·5)	-25·0 (-53·5–17·5)	-34·0 (-48·8–-16·0)
Fiji	-5·61 (-25·3–16·0)	-9·62 (-53·5–59·7)	-7·91 (-27·2–14·0)
Guam	-23·7 (-40·4–-2·63)	-25·4 (-54·9–20·4)	-25·2 (-40·5–-6·46)
Kiribati	55·2 (32·1–81·4)	69·0 (-2·44–179)	57·6 (28·6–89·5)
Marshall Islands	24·7 (-0·0543–54·5)	103 (-6·88–266)	34·9 (7·48–64·0)
Micronesia (Federated States of)	15·4 (-1·27–33·9)	22·8 (-25·3–93·3)	16·8 (-4·03–40·4)
Nauru	10·1 (-11·0–35·1)	2·34 (-34·6–50·7)	5·53 (-17·1–31·5)
Niue	-44·5 (-59·3–-25·6)	-29·4 (-67·0–37·7)	-40·6 (-57·5–-18·7)
Northern Mariana Islands	-23·5 (-39·6–-4·35)	-49·3 (-69·8–-19·0)	-35·4 (-49·2–-17·5)
Palau	-16·9 (-33·0–3·50)	-7·35 (-45·2–54·7)	-15·2 (-32·4–3·87)
Papua New Guinea	98·0 (63·4–136)	104 (20·1–234)	97·5 (60·9–142)
Samoa	9·80 (-15·4–42·9)	2·91 (-48·3–99·0)	6·00 (-20·8–37·6)
Solomon Islands	82·8 (51·4–120)	79·8 (0·939–212)	79·4 (45·7–120)
Tokelau	-23·3 (-42·1–-1·10)	-11·6 (-58·0–58·8)	-21·2 (-41·3–1·32)
Tonga	-21·9 (-35·5–-5·43)	14·8 (-40·3–91·8)	-15·1 (-31·6–2·01)
Tuvalu	76·0 (39·5–124)	84·0 (-10·7–274)	74·4 (35·1–123)
Vanuatu	46·5 (19·0–76·1)	66·4 (-17·4–200)	48·7 (19·9–81·7)
Cambodia	60·2 (19·8–113)	134 (-5·97–402)	65·0 (23·2–116)
Indonesia	15·1 (2·36–29·1)	89·3 (-22·0–293)	17·1 (4·09–31·3)
Lao People's Democratic Republic	116 (64·6–178)	164 (6·93–433)	118 (68·8–178)
Malaysia	21·6 (-0·340–46·5)	42·7 (-37·9–185)	22·3 (0·548–45·9)

Maldives	110 (63·9–164)	56·0 (-36·2–208)	99·9 (56·1–149)
Mauritius	-18·1 (-31·3–-2·31)	11·9 (-45·9–104)	-14·9 (-28·7–2·12)
Myanmar	-13·3 (-28·8–5·35)	-20·5 (-68·9–75·6)	-14·2 (-28·8–2·95)
Philippines	14·3 (-3·05–35·1)	45·5 (-24·2–159)	17·5 (-0·0488–37·7)
Seychelles	-5·37 (-26·2–19·9)	-17·5 (-58·1–48·9)	-9·05 (-29·3–14·3)
Sri Lanka	-21·9 (-42·1–2·60)	-41·2 (-77·1–30·7)	-23·8 (-43·2–-0·108)
Thailand	-35·0 (-45·0–-24·2)	-26·2 (-65·2–44·7)	-34·7 (-44·6–-23·7)
Timor-Leste	71·2 (47·9–96·6)	258 (51·8–655)	76·7 (52·9–105)
Viet Nam	-19·2 (-32·6–-3·18)	17·8 (-56·7–166)	-18·4 (-31·4–-3·23)
**Sub-Saharan Africa**	**66·3 (54·8–77·4)**	**82·5 (50·2–120)**	**68·9 (57·6–80·4)**
Angola	128 (52·8–226)	182 (8·59–493)	131 (60·8–218)
Central African Republic	43·9 (-1·04–107)	76·0 (-32·5–272)	45·5 (2·25–102)
Congo	127 (56·6–217)	221 (6·33–625)	135 (65·1–218)
Democratic Republic of the Congo	116 (45·5–209)	166 (1·21–492)	117 (48·7–206)
Equatorial Guinea	463 (305–666)	308 (50·6–823)	426 (277–607)
Gabon	124 (60·1–213)	190 (20·7–510)	132 (69·9–214)
Burundi	7·67 (-27·2–59·1)	38·5 (-46·7–217)	10·3 (-23·1–63·2)
Comoros	45·6 (4·39–97·2)	65·0 (-36·5–263)	46·0 (7·42–94·7)
Djibouti	111 (48·9–185)	162 (11·9–406)	115 (57·5–185)
Eritrea	87·8 (22·2–171)	107 (-20·4–339)	87·7 (24·5–167)
Ethiopia	150 (60·3–270)	151 (-4·67–456)	145 (65·4–250)
Kenya	33·5 (-5·82–82·6)	58·6 (-33·9–223)	35·5 (-2·97–81·5)
Madagascar	55·0 (20·8–96·3)	103 (-20·4–348)	57·5 (23·5–99·6)
Malawi	131 (60·3–222)	274 (32·3–720)	145 (75·9–235)
Mozambique	139 (63·9–236)	230 (30·4–629)	155 (71·4–263)
Rwanda	95·4 (23·0–184)	101 (-25·6–326)	93·5 (27·9–179)
Somalia	190 (94·4–312)	183 (10·9–506)	184 (97·4–299)

South Sudan	17·4 (-20·7–66·2)	60·5 (-35·7–265)	20·4 (-18·0–68·5)
Uganda	109 (47·0–189)	307 (70·6–733)	142 (73·9–231)
United Republic of Tanzania	37·8 (-4·32–96·4)	102 (-16·0–303)	45·7 (5·92–96·6)
Zambia	191 (112–291)	410 (123–918)	231 (144–348)
Botswana	64·7 (24·1–115)	58·2 (-24·5–203)	60·3 (20·0–111)
Eswatini	1·37 (-26·7–36·0)	12·6 (-56·3–132)	1·67 (-25·0–33·3)
Lesotho	70·0 (29·6–122)	29·1 (-56·2–212)	66·0 (29·0–113)
Namibia	37·4 (1·03–84·7)	67·3 (-23·3–236)	43·4 (2·32–90·2)
South Africa	13·2 (-5·31–34·9)	20·1 (-30·3–94·6)	14·4 (-6·66–39·3)
Zimbabwe	51·2 (11·5–104)	326 (61·6–811)	67·1 (23·2–121)
Benin	71·3 (13·3–143)	112 (-18·6–331)	74·9 (20·2–145)
Burkina Faso	86·9 (34·1–158)	101 (-21·9–338)	86·7 (36·3–153)
Cabo Verde	-19·9 (-48·3–15·8)	1·63 (-60·2–121)	-17·4 (-42·4–16·1)
Cameroon	137 (62·2–243)	256 (45·5–608)	149 (75·0–246)
Chad	107 (46·9–202)	120 (-19·0–381)	106 (47·1–187)
Côte d'Ivoire	147 (75·0–235)	158 (4·38–440)	145 (78·1–224)
Gambia	83·0 (34·2–140)	170 (4·55–512)	87·4 (40·0–142)
Ghana	145 (58·9–266)	168 (-3·88–473)	144 (69·8–257)
Guinea	102 (45·5–171)	161 (0·671–456)	105 (49·8–174)
Guinea-Bissau	141 (56·5–260)	133 (-14·3–391)	137 (59·9–249)
Liberia	232 (119–387)	423 (102–945)	278 (154–441)
Mali	289 (180–438)	363 (83·0–880)	291 (184–432)
Mauritania	38·8 (8·60–78·2)	84·4 (-0·242–221)	47·3 (16·0–84·9)
Niger	213 (116–339)	266 (49·4–673)	216 (125–331)
Nigeria	9·15 (-22·5–51·7)	51·4 (-43·3–206)	12·2 (-19·9–50·2)
São Tomé and Príncipe	131 (44·3–246)	117 (-17·8–389)	124 (48·3–219)
Senegal	-3·90 (-27·9–28·1)	32·6 (-46·4–188)	-1·68 (-25·3–28·7)

Sierra Leone	119<br>(59·6–192)	117<br>(0·285–330)	114<br>(58·9–190)
Togo	19·4<br>(-17·7–67·8)	39·2<br>(-47·5–194)	20·3<br>(-16·0–65·4)

Supplemental Table S6. Absolute change in number of smokers, by sex, for ages 15-24, from 1990-2019.

Location	Absolute Change Number of Smokers Ages 15-24 1990-2019 Male (Thousands)	Absolute Change Number of Smokers Ages 15-24 1990-2019 Female (Thousands)	Absolute Change Number of Smokers Ages 15-24 1990-2019 Both (Thousands)
**Global**	**-28500 (-34400–-22400)**	**-10100 (-12900–-7320)**	**-38700 (-45200–-31800)**
**Central Europe, Eastern Europe, and Central Asia**	**-6760 (-7320–-6200)**	**-2220 (-2880–-1590)**	**-8980 (-9770–-8140)**
Armenia	-51·5 (-67·8–-35·7)	-1·66 (-6·25–1·51)	-53·1 (-69·9–-36·8)
Azerbaijan	36·3 (-13·0–82·9)	4·30 (-8·84–17·9)	40·6 (-8·79–90·8)
Georgia	-80·6 (-108–-53·1)	-11·0 (-27·5–0·939)	-91·6 (-122–-62·6)
Kazakhstan	-233 (-310–-158)	-29·4 (-105–28·8)	-262 (-366–-164)
Kyrgyzstan	6·36 (-18·3–32·8)	11·3 (0·00936–25·5)	17·7 (-12·5–45·8)
Mongolia	11·8 (-4·23–28·4)	4·22 (-2·82–12·1)	16·0 (-1·35–34·2)
Tajikistan	-6·34 (-36·3–21·6)	0·211 (-7·35–8·32)	-6·13 (-36·5–22·9)
Turkmenistan	-34·4 (-61·4–-8·74)	-3·57 (-12·8–3·58)	-38·0 (-66·6–-12·0)
Uzbekistan	136 (63·7–223)	21·6 (-0·949–54·3)	158 (81·7–247)
Albania	6·67 (-13·8–24·5)	3·72 (-12·2–18·9)	10·4 (-14·0–33·7)
Bosnia and Herzegovina	-33·3 (-54·8–-11·1)	-9·04 (-39·2–16·5)	-42·4 (-80·5–-7·16)
Bulgaria	-173 (-213–-128)	-142 (-224–-66·9)	-315 (-407–-224)
Croatia	-79·0 (-99·5–-58·6)	-59·6 (-94·7–-26·1)	-139 (-179–-97·6)
Czechia	-165 (-211–-120)	-59·3 (-105–-17·9)	-224 (-282–-157)
Hungary	-157 (-200–-113)	-86·2 (-155–-23·4)	-243 (-320–-166)
Montenegro	-4·58 (-7·73–-1·51)	-4·21 (-9·74–0·336)	-8·79 (-15·2–-3·03)
North Macedonia	-12·2 (-24·7–0·0245)	-11·6 (-31·9–6·25)	-23·9 (-46·8–-1·05)
Poland	-734 (-834–-637)	-257 (-370–-146)	-991 (-1140–-838)
Romania	-569 (-675–-471)	-128 (-216–-45·0)	-697 (-822–-569)

Serbia	-20·0 (-58·0–17·9)	39·0 (-25·5–98·9)	19·0 (-51·0–92·7)
Slovakia	-77·8 (-104–-52·8)	-33·7 (-74·8–2·66)	-112 (-162–-68·2)
Slovenia	-27·0 (-36·7–-16·2)	-14·8 (-29·1–-1·44)	-41·9 (-60·1–-25·4)
Belarus	-176 (-236–-119)	-43·0 (-129–28·2)	-219 (-323–-126)
Estonia	-28·3 (-33·4–-23·2)	-10·2 (-16·4–-4·83)	-38·4 (-46·5–-31·1)
Latvia	-60·8 (-71·6–-50·9)	-29·1 (-43·9–-14·6)	-89·9 (-109–-73·2)
Lithuania	-64·0 (-78·6–-49·1)	-2·20 (-18·0–11·8)	-66·2 (-88·3–-45·0)
Republic of Moldova	-42·3 (-64·9–-21·5)	-10·8 (-25·5–2·40)	-53·1 (-78·3–-27·3)
Russian Federation	-2910 (-3370–-2460)	-856 (-1390–-345)	-3770 (-4480–-3050)
Ukraine	-1220 (-1410–-1020)	-503 (-786–-260)	-1720 (-2060–-1420)
**High-income**	**-9780 (-10600–-8930)**	**-7730 (-8740–-6680)**	**-17500 (-18800–-16200)**
Australia	-244 (-298–-189)	-271 (-345–-180)	-516 (-607–-402)
New Zealand	-22·1 (-30·2–-12·7)	-48·7 (-59·8–-36·7)	-70·8 (-84·9–-56·2)
Brunei Darussalam	0·984 (-1·91–3·89)	0·465 (-0·917–1·91)	1·45 (-1·77–4·84)
Japan	-2670 (-3110–-2220)	-533 (-912–-224)	-3200 (-3760–-2700)
Republic of Korea	-903 (-1190–-612)	-141 (-399–74·7)	-1040 (-1440–-669)
Singapore	-38·4 (-59·3–-16·6)	-18·2 (-40·0–0·142)	-56·7 (-87·1–-27·2)
Canada	-299 (-382–-214)	-325 (-446–-189)	-625 (-770–-456)
Greenland	-0·933 (-1·42–-0·455)	-0·493 (-1·30–0·269)	-1·43 (-2·35–-0·521)
United States of America	-1140 (-1650–-545)	-1810 (-2450–-1090)	-2960 (-3740–-2000)
Argentina	295 (58·7–517)	-8·22 (-332–325)	287 (-126–699)
Chile	-4·70 (-89·7–87·2)	-94·8 (-307–121)	-99·5 (-338–129)
Uruguay	-21·0 (-39·5–-2·25)	-33·9 (-64·3–-3·11)	-54·9 (-92·3–-18·8)
Andorra	-0·491 (-0·864–-0·0929)	-0·409 (-1·09–0·322)	-0·900 (-1·63–-0·0959)
Austria	-57·1 (-93·8–-21·2)	-74·0 (-115–-30·9)	-131 (-185–-75·6)
Belgium	-128 (-155–-99·5)	-81·7 (-116–-44·2)	-210 (-256–-168)
Cyprus	4·45 (-1·59–10·6)	-0·581 (-8·95–6·99)	3·87 (-6·64–13·5)

Denmark	-68·6<br>(-82·0–-54·7)	-80·8<br>(-99·9–-60·6)	-149<br>(-173–-124)
Finland	-36·3<br>(-49·4–-23·6)	-19·4<br>(-37·1–0·288)	-55·7<br>(-77·3–-33·1)
France	-782<br>(-968–-592)	-670<br>(-928–-402)	-1450<br>(-1780–-1140)
Germany	-827<br>(-1050–-604)	-753<br>(-1010–-481)	-1580<br>(-1900–-1230)
Greece	-157<br>(-186–-128)	-170<br>(-206–-130)	-327<br>(-375–-276)
Iceland	-3·67<br>(-4·66–-2·61)	-3·90<br>(-5·95–-1·87)	-7·57<br>(-9·76–-5·31)
Ireland	-44·2<br>(-56·1–-31·1)	-3·82<br>(-24·6–17·7)	-48·0<br>(-72·6–-22·8)
Israel	12·3<br>(-17·9–45·1)	-16·9<br>(-48·8–22·9)	-4·62<br>(-52·8–45·9)
Italy	-659<br>(-793–-525)	-488<br>(-621–-344)	-1150<br>(-1340–-943)
Luxembourg	-0·118<br>(-1·99–2·03)	2·44<br>(-0·0879–4·85)	2·32<br>(-0·661–5·60)
Malta	-4·54<br>(-6·36–-2·65)	-2·36<br>(-5·24–0·223)	-6·90<br>(-10·0–-3·60)
Monaco	-0·104<br>(-0·256–0·0433)	-0·0757<br>(-0·339–0·188)	-0·180<br>(-0·485–0·116)
Netherlands	-207<br>(-256–-153)	-223<br>(-279–-160)	-430<br>(-504–-350)
Norway	-69·8<br>(-85·3–-53·3)	-81·9<br>(-103–-59·6)	-152<br>(-176–-125)
Portugal	-147<br>(-177–-115)	-33·2<br>(-69·8–4·42)	-180<br>(-228–-133)
San Marino	-0·253<br>(-0·400–-0·102)	-0·260<br>(-0·572–0·0280)	-0·512<br>(-0·872–-0·190)
Spain	-877<br>(-986–-759)	-904<br>(-1040–-762)	-1780<br>(-1940–-1600)
Sweden	-51·2<br>(-74·9–-27·5)	-89·8<br>(-128–-49·9)	-141<br>(-186–-94·1)
Switzerland	-49·9<br>(-72·0–-26·3)	-55·2<br>(-82·8–-24·0)	-105<br>(-142–-65·8)
United Kingdom	-576<br>(-708–-425)	-697<br>(-876–-502)	-1270<br>(-1490–-1050)
**Latin America and Caribbean**	**-4610<br>(-5510–-3690)**	**-3190<br>(-4160–-2260)**	**-7800<br>(-9140–-6510)**
Bolivia (Plurinational State of)	85·6<br>(41·8–132)	47·9<br>(-3·21–95·5)	133<br>(69·4–198)
Ecuador	155<br>(88·9–219)	38·1<br>(-2·97–81·6)	193<br>(120–269)
Peru	35·3<br>(-75·0–152)	3·34<br>(-120–112)	38·7<br>(-129–200)
Antigua and Barbuda	0·268<br>(0·0897–0·457)	0·216<br>(-0·0241–0·467)	0·484<br>(0·204–0·795)
Bahamas	1·23<br>(0·280–2·21)	0·273<br>(-0·594–1·17)	1·50<br>(0·188–2·83)
Barbados	-0·632<br>(-1·62–0·393)	-0·0601<br>(-1·24–1·04)	-0·692<br>(-2·29–0·805)

Belize	4·48<br>(2·43–6·50)	1·50<br>(0·119–2·94)	5·97<br>(3·67–8·44)
Bermuda	-0·215<br>(-0·422–-0·0175)	-0·108<br>(-0·359–0·0953)	-0·323<br>(-0·636–-0·0398)
Cuba	-182<br>(-260–-107)	-125<br>(-239–-38·3)	-307<br>(-443–-185)
Dominica	-0·262<br>(-0·598–0·0427)	-0·270<br>(-0·715–0·137)	-0·533<br>(-1·05–0·0311)
Dominican Republic	16·1<br>(-1·76–35·9)	8·89<br>(-14·9–33·9)	25·0<br>(-4·81–57·7)
Grenada	0·355<br>(-0·0477–0·774)	0·0665<br>(-0·329–0·439)	0·421<br>(-0·158–0·982)
Guyana	1·51<br>(-3·27–5·78)	-0·632<br>(-3·99–2·23)	0·879<br>(-4·55–6·00)
Haiti	10·1<br>(-13·9–34·1)	3·40<br>(-20·9–28·2)	13·5<br>(-20·5–44·7)
Jamaica	-2·29<br>(-15·1–9·85)	3·67<br>(-8·89–15·2)	1·38<br>(-16·0–18·4)
Puerto Rico	-28·3<br>(-41·9–-15·9)	-12·2<br>(-26·7–-0·399)	-40·5<br>(-59·6–-23·3)
Saint Kitts and Nevis	0·0267<br>(-0·0905–0·161)	0·0119<br>(-0·0771–0·119)	0·0386<br>(-0·120–0·198)
Saint Lucia	-0·393<br>(-1·14–0·365)	-0·0202<br>(-0·864–0·668)	-0·413<br>(-1·51–0·647)
Saint Vincent and the Grenadines	-0·346<br>(-0·998–0·247)	-0·216<br>(-0·793–0·263)	-0·562<br>(-1·40–0·179)
Suriname	-1·11<br>(-4·54–2·45)	-0·108<br>(-3·13–2·59)	-1·22<br>(-5·89–2·91)
Trinidad and Tobago	-8·43<br>(-16·5–-1·06)	-2·74<br>(-10·0–3·55)	-11·2<br>(-22·0–-1·10)
United States Virgin Islands	-0·216<br>(-0·396–-0·0442)	-0·0814<br>(-0·259–0·0855)	-0·298<br>(-0·551–-0·0616)
Colombia	-546<br>(-815–-296)	-347<br>(-688–-38·2)	-893<br>(-1320–-487)
Costa Rica	-23·5<br>(-44·2–-3·94)	-21·6<br>(-49·9–2·38)	-45·1<br>(-76·4–-12·7)
El Salvador	35·2<br>(3·74–65·0)	18·6<br>(1·93–36·5)	53·8<br>(18·2–89·0)
Guatemala	246<br>(166–327)	102<br>(49·3–164)	348<br>(245–450)
Honduras	109<br>(60·2–156)	46·4<br>(19·6–78·1)	155<br>(99·3–214)
Mexico	-1100<br>(-1670–-533)	-508<br>(-1130–81·9)	-1610<br>(-2450–-805)
Nicaragua	20·5<br>(-12·1–55·5)	15·8<br>(-15·0–44·8)	36·3<br>(-6·27–81·6)
Panama	-4·97<br>(-18·4–7·99)	-5·93<br>(-23·3–11·5)	-10·9<br>(-33·6–10·5)
Venezuela (Bolivarian Republic of)	-146<br>(-283–-7·43)	-65·8<br>(-200–60·9)	-212<br>(-412–-24·7)
Brazil	-3280<br>(-3890–-2620)	-2400<br>(-2920–-1870)	-5680<br>(-6460–-4870)
Paraguay	-4·91<br>(-36·4–28·9)	2·17<br>(-28·8–33·8)	-2·74<br>(-48·1–45·7)

**North Africa and Middle East**	**4410 (3630–5200)**	**1110 (676–1570)**	**5520 (4630–6430)**
Afghanistan	575 (453–702)	87·9 (42·5–154)	663 (529–808)
Algeria	141 (-71·2–338)	-4·36 (-53·4–39·3)	136 (-74·4–340)
Bahrain	14·2 (10·0–18·9)	3·76 (1·73–6·20)	18·0 (13·2–23·1)
Egypt	1240 (864–1590)	69·8 (-25·9–182)	1310 (922–1680)
Iran (Islamic Republic of)	95·8 (-156–357)	30·0 (-128–174)	126 (-165–413)
Iraq	688 (475–906)	90·8 (13·1–194)	779 (549–1010)
Jordan	360 (307–410)	116 (66·2–174)	476 (403–556)
Kuwait	41·1 (24·6–59·8)	11·7 (3·58–20·5)	52·9 (33·5–72·4)
Lebanon	48·1 (25·9–71·1)	12·9 (-7·62–32·9)	61·0 (31·9–89·2)
Libya	36·5 (1·58–75·2)	3·76 (-4·63–13·8)	40·2 (4·27–79·9)
Morocco	-107 (-247–44·0)	2·91 (-45·8–49·3)	-104 (-253–47·1)
Oman	23·5 (11·8–37·5)	3·00 (0·124–6·92)	26·5 (14·4–40·3)
Palestine	89·9 (60·8–118)	12·2 (4·33–22·9)	102 (73·1–131)
Qatar	56·8 (44·3–71·4)	4·12 (1·62–7·24)	60·9 (47·8–75·7)
Saudi Arabia	433 (321–548)	43·3 (10·0–88·0)	477 (362–605)
Sudan	279 (140–427)	42·6 (-6·46–112)	322 (174–490)
Syrian Arab Republic	-25·4 (-139–92·1)	0·802 (-91·1–92·7)	-24·5 (-171–118)
Tunisia	-66·0 (-129–-3·91)	-7·60 (-31·6–13·5)	-73·6 (-141–-7·13)
Turkey	97·6 (-387–542)	468 (127–840)	565 (-19·1–1150)
United Arab Emirates	29·5 (15·0–43·0)	9·85 (4·44–17·7)	39·3 (24·1–55·0)
Yemen	362 (242–490)	104 (29·0–199)	466 (323–614)
**South Asia**	**5710 (2240–9360)**	**575 (-1100–2380)**	**6280 (2380–10400)**
Bangladesh	311 (-398–1050)	54·0 (-69·0–195)	365 (-365–1110)
Bhutan	-0·332 (-5·58–4·56)	1·29 (-2·37–4·40)	0·962 (-5·27–7·03)
India	4670 (1310–8120)	393 (-1230–2120)	5060 (1300–8910)
Nepal	200 (77·3–316)	-33·9 (-130–51·2)	166 (18·3–304)

Pakistan	530 (-269–1290)	160 (-117–459)	690 (-148–1510)
**Southeast Asia, East Asia, and Oceania**	**-21600 (-26000–-16900)**	**298 (-1170–1800)**	**-21300 (-25800–-16400)**
China	-22100 (-26400–-17800)	-106 (-1510–1220)	-22200 (-26700–-17700)
Democratic People's Republic of Korea	44·4 (-89·5–176)	3·05 (-36·1–42·2)	47·4 (-110–185)
Taiwan (Province of China)	-209 (-340–-81·5)	-42·6 (-145–33·6)	-252 (-407–-107)
American Samoa	0·180 (-0·313–0·662)	0·0576 (-0·679–0·794)	0·238 (-0·632–1·10)
Cook Islands	-0·314 (-0·461–-0·172)	-0·181 (-0·450–0·0856)	-0·495 (-0·806–-0·197)
Fiji	-1·85 (-8·58–4·52)	-1·82 (-9·26–5·54)	-3·67 (-13·2–5·59)
Guam	-1·31 (-2·52–-0·111)	-0·836 (-2·27–0·387)	-2·15 (-3·99–-0·452)
Kiribati	2·07 (1·34–2·78)	1·07 (-0·0547–2·23)	3·14 (1·82–4·47)
Marshall Islands	0·403 (-0·00108–0·822)	0·286 (-0·0325–0·584)	0·688 (0·168–1·17)
Micronesia (Federated States of)	0·882 (-0·0796–1·80)	0·594 (-1·10–2·13)	1·48 (-0·414–3·24)
Nauru	0·0423 (-0·0552–0·138)	-0·00104 (-0·185–0·168)	0·0413 (-0·171–0·241)
Niue	-0·0212 (-0·0339–-0·00990)	-0·00933 (-0·0278–0·00601)	-0·0305 (-0·0522–-0·0113)
Northern Mariana Islands	-0·471 (-0·859–-0·0741)	-0·793 (-1·43–-0·220)	-1·26 (-2·01–-0·518)
Palau	-0·108 (-0·236–0·0193)	-0·0342 (-0·194–0·0948)	-0·142 (-0·345–0·0295)
Papua New Guinea	226 (162–290)	100 (27·9–173)	326 (226–426)
Samoa	0·449 (-0·924–1·82)	-0·103 (-1·48–1·26)	0·347 (-1·85–2·30)
Solomon Islands	13·4 (9·53–17·3)	4·74 (0·101–8·98)	18·1 (12·5–23·7)
Tokelau	-0·0133 (-0·0270–-0·000504)	-0·00378 (-0·0183–0·00988)	-0·0171 (-0·0363–0·000956)
Tonga	-0·942 (-1·67–-0·205)	0·101 (-0·710–0·766)	-0·841 (-1·92–0·0980)
Tuvalu	0·202 (0·121–0·286)	0·0772 (-0·0183–0·171)	0·279 (0·153–0·402)
Vanuatu	3·11 (1·41–4·65)	1·04 (-0·444–2·50)	4·14 (2·00–6·41)
Cambodia	83·6 (33·8–139)	16·5 (-1·09–39·4)	100 (42·9–161)
Indonesia	1220 (206–2220)	205 (-92·9–601)	1430 (369–2510)
Lao People's Democratic Republic	107 (68·8–143)	13·9 (1·01–28·1)	121 (82·1–159)
Malaysia	168 (-2·87–335)	19·3 (-35·8–73·7)	187 (5·23–357)

Maldives	7·59 (5·08–10·2)	0·475 (-0·696–1·56)	8·06 (5·23–11·0)
Mauritius	-8·93 (-17·0–-0·968)	0·237 (-5·76–5·40)	-8·69 (-18·6–1·00)
Myanmar	-253 (-578–83·1)	-33·7 (-133–50·0)	-287 (-625–49·8)
Philippines	429 (-105–972)	183 (-180–552)	612 (-1·78–1250)
Seychelles	-0·183 (-0·900–0·510)	-0·197 (-0·786–0·272)	-0·380 (-1·27–0·488)
Sri Lanka	-80·4 (-175–7·28)	-13·3 (-36·6–5·40)	-93·7 (-196–-0·379)
Thailand	-861 (-1220–-518)	-59·1 (-177–58·9)	-920 (-1290–-559)
Timor-Leste	32·2 (23·7–40·0)	3·79 (1·23–6·97)	36·0 (27·2–45·3)
Viet Nam	-467 (-843–-71·8)	4·27 (-67·9–73·8)	-462 (-844–-75·3)
**Sub-Saharan Africa**	**4110 (3540–4670)**	**1050 (702–1410)**	**5160 (4480–5840)**
Angola	117 (59·8–177)	20·9 (1·66–46·1)	138 (78·1–200)
Central African Republic	14·7 (-0·456–31·8)	2·63 (-2·56–9·51)	17·3 (1·13–36·2)
Congo	36·2 (19·6–54·3)	7·61 (0·335–17·4)	43·8 (25·1–64·6)
Democratic Republic of the Congo	483 (231–759)	28·3 (0·359–66·6)	511 (259–784)
Equatorial Guinea	37·4 (28·4–47·1)	5·03 (1·37–10·5)	42·4 (32·4–53·8)
Gabon	17·4 (10·1–24·6)	6·28 (1·20–13·0)	23·7 (14·9–32·8)
Burundi	3·44 (-20·5–30·8)	2·74 (-7·75–15·6)	6·18 (-19·8–37·3)
Comoros	3·68 (0·449–7·00)	0·878 (-1·14–2·90)	4·56 (0·808–8·40)
Djibouti	12·2 (6·39–18·0)	2·70 (0·302–5·65)	14·9 (8·73–21·2)
Eritrea	23·9 (8·03–41·9)	2·45 (-0·765–6·78)	26·4 (9·42–44·9)
Ethiopia	287 (147–434)	46·0 (-2·46–114)	333 (178–490)
Kenya	122 (-27·7–274)	34·9 (-43·1–114)	157 (-16·0–314)
Madagascar	228 (98·7–364)	35·1 (-14·9–92·2)	263 (121–416)
Malawi	96·6 (54·8–140)	27·4 (5·66–55·6)	124 (76·5–172)
Mozambique	163 (88·1–242)	78·9 (17·1–161)	241 (133–348)
Rwanda	40·3 (12·9–67·2)	4·61 (-2·07–13·5)	44·9 (15·8–73·9)
Somalia	148 (83·5–227)	25·9 (2·31–62·0)	174 (104–256)

South Sudan	14·0 (-24·3–51·3)	5·02 (-6·10–19·2)	19·0 (-21·2–58·6)
Uganda	172 (86·8–259)	109 (40·0–210)	281 (165–407)
United Republic of Tanzania	107 (-15·8–238)	52·6 (-16·2–137)	159 (24·0–309)
Zambia	196 (140–257)	110 (51·1–183)	306 (223–395)
Botswana	20·2 (9·07–31·2)	4·91 (-4·15–13·7)	25·1 (9·96–40·5)
Eswatini	0·0451 (-3·37–3·52)	0·0584 (-1·71–1·92)	0·103 (-3·71–3·99)
Lesotho	27·4 (14·3–41·2)	0·391 (-2·73–4·03)	27·8 (14·7–41·8)
Namibia	10·4 (0·345–20·8)	7·02 (-4·30–19·2)	17·4 (1·28–32·9)
South Africa	148 (-66·9–369)	80·7 (-189–357)	228 (-123–582)
Zimbabwe	83·6 (22·5–145)	35·4 (11·1–68·8)	119 (51·3–185)
Benin	28·2 (6·51–50·8)	7·77 (-2·69–19·4)	36·0 (12·0–59·9)
Burkina Faso	127 (57·7–200)	14·8 (-4·75–42·0)	141 (67·7–221)
Cabo Verde	-0·759 (-2·06–0·459)	-0·0752 (-0·846–0·665)	-0·834 (-2·34–0·568)
Cameroon	119 (64·7–179)	31·9 (9·19–60·5)	151 (90·1–217)
Chad	76·5 (39·6–121)	20·5 (-5·10–55·4)	97·0 (49·5–153)
Côte d'Ivoire	289 (179–402)	50·0 (2·27–115)	339 (216–470)
Gambia	17·6 (8·69–25·9)	2·60 (0·109–5·87)	20·2 (11·3–29·3)
Ghana	74·8 (37·3–118)	23·1 (-0·918–51·8)	98·0 (55·5–146)
Guinea	114 (60·0–169)	11·6 (0·0736–27·6)	126 (69·3–186)
Guinea-Bissau	5·43 (2·70–8·86)	0·817 (-0·143–2·09)	6·25 (3·29–9·93)
Liberia	16·4 (10·0–24·7)	12·0 (4·67–20·7)	28·4 (18·7–40·9)
Mali	262 (186–344)	17·9 (6·23–34·6)	280 (205–362)
Mauritania	28·7 (7·44–53·8)	17·4 (-0·0842–37·5)	46·1 (17·4–76·1)
Niger	207 (136–285)	35·5 (11·1–72·3)	243 (164–326)
Nigeria	56·3 (-182–326)	31·5 (-52·3–129)	87·9 (-181–358)
São Tomé and Príncipe	0·303 (0·133–0·518)	0·0572 (-0·0180–0·151)	0·360 (0·171–0·579)
Senegal	-9·54 (-62·2–46·4)	3·84 (-14·2–23·5)	-5·70 (-62·1–51·6)

Sierra Leone	78·1<br>(46·9–112)	33·1<br>(0·123–72·8)	111<br>(67·6–164)
Togo	8·41<br>(-10·2–27·3)	2·10<br>(-7·06–10·3)	10·5<br>(-10·1–30·8)

Supplemental Table S7. Age window of initiation and mean age of initiation among current smokers ages 20-54, by sex. Age window of initiation reports the 10th and 90th percentiles of the distribution of initiation ages observed in the population.

Location	Age Window of Initiation Male 2019 (10th-90th Percentile)	Age Window of Initiation Female 2019 (10th-90th Percentile)	Mean Age of Initiation Male 2019	Mean Age of Initiation Female 2019	Mean Age of Initiation Both 2019
**Global**	**13 - 25**	**14 - 29**	**19·2 (19·0–19·3)**	**19·6 (19·4–19·8)**	**19·2 (19·1–19·4)**
**Central Europe, Eastern Europe, and Central Asia**	**13 - 22**	**14 - 27**	**17·1 (17·0–17·3)**	**19·0 (18·8–19·2)**	**17·7 (17·6–17·8)**
Armenia	14 - 21	18 - 35	17·3 (17·0–17·7)	25·0 (24·1–26·0)	17·8 (17·5–18·1)
Azerbaijan	13 - 23	14 - 31	17·6 (17·2–18·0)	21·1 (20·2–22·0)	17·8 (17·5–18·2)
Georgia	14 - 23	16 - 31	18·1 (17·8–18·5)	22·1 (21·3–22·9)	18·6 (18·3–18·9)
Kazakhstan	13 - 22	14 - 27	17·0 (16·6–17·4)	19·6 (18·9–20·3)	17·4 (17·1–17·7)
Kyrgyzstan	14 - 23	15 - 25	18·1 (17·8–18·5)	19·3 (18·8–19·9)	18·3 (17·9–18·6)
Mongolia	14 - 25	16 - 38	19·0 (18·6–19·4)	24·6 (23·6–25·6)	19·8 (19·3–20·2)
Tajikistan	14 - 23	15 - 31	17·9 (17·5–18·4)	21·3 (20·3–22·3)	18·2 (17·8–18·6)
Turkmenistan	14 - 23	15 - 31	17·9 (17·5–18·3)	21·7 (20·8–22·7)	18·4 (17·9–18·8)
Uzbekistan	14 - 23	15 - 31	18·0 (17·6–18·5)	21·4 (20·4–22·4)	18·3 (17·9–18·7)
Albania	14 - 25	16 - 32	19·2 (18·8–19·5)	22·5 (21·6–23·6)	19·8 (19·4–20·2)
Bosnia and Herzegovina	14 - 23	14 - 24	17·9 (17·5–18·3)	18·9 (18·4–19·6)	18·3 (18·0–18·7)
Bulgaria	14 - 22	15 - 24	17·5 (17·2–17·8)	18·9 (18·5–19·3)	18·0 (17·8–18·3)
Croatia	14 - 22	14 - 23	17·9 (17·5–18·2)	18·3 (17·9–18·8)	18·1 (17·8–18·4)
Czechia	14 - 22	14 - 23	17·6 (17·3–17·9)	18·2 (17·8–18·5)	17·8 (17·6–18·0)
Hungary	13 - 21	14 - 23	17·0 (16·7–17·3)	17·8 (17·3–18·3)	17·3 (17·1–17·6)
Montenegro	14 - 23	14 - 25	17·9 (17·5–18·3)	19·1 (18·5–19·7)	18·5 (18·1–18·8)
North Macedonia	14 - 23	14 - 24	17·8 (17·4–18·3)	18·8 (18·3–19·4)	18·2 (17·9–18·6)
Poland	15 - 22	15 - 24	18·2 (17·9–18·5)	19·2 (18·8–19·6)	18·6 (18·4–18·9)
Romania	14 - 23	15 - 26	18·0 (17·7–18·4)	19·7 (19·3–20·1)	18·7 (18·4–18·9)

Serbia	14 - 22	14 - 24	17·9 (17·5–18·3)	18·9 (18·3–19·6)	18·4 (18·0–18·8)
Slovakia	14 - 22	14 - 23	17·6 (17·3–17·9)	18·2 (17·7–18·6)	17·8 (17·5–18·1)
Slovenia	14 - 23	14 - 24	18·0 (17·6–18·4)	18·7 (18·3–19·2)	18·3 (18·0–18·6)
Belarus	12 - 20	13 - 25	15·6 (15·3–16·0)	18·4 (17·8–19·0)	16·6 (16·3–16·9)
Estonia	13 - 21	14 - 26	17·0 (16·7–17·4)	19·1 (18·6–19·6)	17·7 (17·5–18·0)
Latvia	13 - 22	14 - 29	17·1 (16·8–17·5)	20·2 (19·7–20·7)	18·0 (17·8–18·3)
Lithuania	13 - 23	15 - 29	17·8 (17·4–18·2)	20·4 (19·9–21·0)	18·7 (18·4–19·1)
Republic of Moldova	12 - 21	14 - 28	16·6 (16·2–16·9)	19·9 (19·4–20·6)	17·1 (16·8–17·4)
Russian Federation	12 - 21	13 - 25	16·6 (16·3–16·9)	18·6 (18·2–19·0)	17·1 (16·9–17·4)
Ukraine	12 - 21	14 - 26	16·6 (16·2–16·9)	19·4 (18·9–20·0)	17·3 (17·0–17·6)
**High-income**	**13 - 21**	**13 - 22**	**17·0 (16·9–17·1)**	**17·2 (17·1–17·4)**	**17·1 (17·0–17·2)**
Australia	13 - 21	13 - 22	16·9 (16·5–17·3)	17·1 (16·5–17·6)	17·0 (16·6–17·3)
New Zealand	13 - 21	13 - 22	16·8 (16·5–17·2)	17·1 (16·6–17·7)	17·0 (16·7–17·3)
Brunei Darussalam	13 - 21	13 - 22	16·8 (16·4–17·3)	17·1 (16·5–17·8)	16·9 (16·5–17·3)
Japan	13 - 22	13 - 22	17·0 (16·6–17·4)	17·2 (16·7–17·8)	17·0 (16·7–17·4)
Republic of Korea	13 - 22	13 - 22	16·9 (16·6–17·3)	17·0 (16·4–17·6)	16·9 (16·6–17·3)
Singapore	13 - 22	13 - 22	17·0 (16·6–17·4)	17·1 (16·5–17·7)	17·0 (16·7–17·3)
Canada	13 - 23	13 - 24	17·3 (16·9–17·7)	17·5 (17·0–18·1)	17·4 (17·1–17·8)
Greenland	12 - 23	13 - 24	17·3 (16·9–17·7)	17·5 (16·9–18·2)	17·4 (17·1–17·7)
United States of America	13 - 22	13 - 23	17·3 (17·0–17·6)	17·5 (17·1–17·9)	17·4 (17·1–17·6)
Argentina	13 - 21	13 - 22	16·6 (16·3–16·9)	17·3 (16·9–17·8)	16·9 (16·6–17·1)
Chile	13 - 21	13 - 23	16·6 (16·3–17·0)	17·4 (16·9–18·0)	17·0 (16·7–17·3)
Uruguay	13 - 21	13 - 24	16·8 (16·5–17·2)	17·6 (17·1–18·1)	17·2 (16·9–17·5)
Andorra	13 - 21	13 - 22	16·9 (16·5–17·3)	17·1 (16·5–17·6)	17·0 (16·6–17·3)
Austria	14 - 20	14 - 21	16·7 (16·4–17·0)	17·1 (16·8–17·5)	16·9 (16·7–17·1)
Belgium	13 - 21	13 - 21	16·6 (16·3–16·9)	16·7 (16·3–17·2)	16·7 (16·4–16·9)
Cyprus	14 - 22	14 - 22	17·6 (17·2–17·9)	17·8 (17·2–18·4)	17·6 (17·3–17·9)

Denmark	13 - 21	13 - 21	16·5 (16·2–16·9)	16·4 (15·9–16·8)	16·4 (16·2–16·7)
Finland	13 - 20	13 - 21	16·6 (16·2–16·9)	16·6 (16·2–17·1)	16·6 (16·4–16·9)
France	13 - 21	13 - 22	16·9 (16·6–17·3)	17·3 (16·9–17·8)	17·1 (16·8–17·4)
Germany	13 - 21	13 - 22	16·8 (16·5–17·1)	17·1 (16·7–17·5)	16·9 (16·7–17·1)
Greece	14 - 22	15 - 25	17·7 (17·4–18·0)	19·4 (19·0–19·8)	18·4 (18·2–18·6)
Iceland	13 - 21	13 - 21	16·8 (16·4–17·2)	16·9 (16·4–17·5)	16·9 (16·5–17·2)
Ireland	13 - 21	13 - 21	16·8 (16·5–17·2)	16·6 (16·2–16·9)	16·7 (16·5–17·0)
Israel	13 - 21	13 - 21	16·8 (16·4–17·2)	16·9 (16·4–17·4)	16·8 (16·5–17·1)
Italy	14 - 20	14 - 21	16·7 (16·5–17·0)	17·3 (16·8–17·7)	17·0 (16·7–17·2)
Luxembourg	13 - 22	13 - 21	17·3 (17·0–17·7)	17·0 (16·5–17·6)	17·2 (16·9–17·5)
Malta	13 - 21	13 - 22	16·7 (16·3–17·1)	16·8 (16·3–17·3)	16·8 (16·4–17·1)
Monaco	13 - 21	13 - 22	16·8 (16·4–17·3)	17·1 (16·5–17·7)	17·0 (16·6–17·3)
Netherlands	13 - 21	13 - 20	17·1 (16·8–17·4)	16·2 (15·8–16·6)	16·7 (16·4–17·0)
Norway	13 - 21	13 - 21	16·8 (16·4–17·2)	17·0 (16·4–17·6)	16·9 (16·6–17·2)
Portugal	13 - 21	14 - 22	16·8 (16·5–17·1)	17·3 (16·8–17·8)	17·0 (16·8–17·3)
San Marino	13 - 21	13 - 21	16·8 (16·4–17·2)	17·0 (16·5–17·6)	16·9 (16·6–17·2)
Spain	13 - 21	13 - 21	16·9 (16·6–17·2)	17·0 (16·6–17·3)	16·9 (16·7–17·2)
Sweden	14 - 21	13 - 20	17·2 (16·8–17·5)	16·3 (15·8–16·7)	16·7 (16·4–17·0)
Switzerland	13 - 21	13 - 22	16·8 (16·4–17·2)	17·0 (16·5–17·5)	16·9 (16·6–17·2)
United Kingdom	13 - 21	13 - 21	16·7 (16·5–17·0)	16·7 (16·3–17·0)	16·7 (16·5–16·9)
**Latin America and Caribbean**	**13 - 23**	**14 - 25**	**17·2 (17·1–17·4)**	**18·6 (18·4–18·8)**	**17·6 (17·5–17·8)**
Bolivia (Plurinational State of)	13 - 22	14 - 26	17·3 (17·0–17·7)	19·1 (18·4–19·9)	17·9 (17·5–18·2)
Ecuador	13 - 22	14 - 25	17·2 (16·8–17·6)	18·7 (18·1–19·4)	17·4 (17·1–17·8)
Peru	13 - 22	14 - 25	17·2 (16·8–17·6)	18·6 (17·9–19·2)	17·7 (17·3–18·1)
Antigua and Barbuda	14 - 24	14 - 25	18·4 (18·0–18·9)	19·4 (18·8–20·1)	18·7 (18·3–19·1)
Bahamas	14 - 24	14 - 25	18·4 (17·9–18·8)	19·4 (18·7–20·0)	18·6 (18·2–19·0)
Barbados	13 - 23	14 - 25	17·9 (17·4–18·3)	19·4 (18·7–20·1)	18·2 (17·9–18·6)

Belize	14 - 24	14 - 25	18·3 (17·9–18·7)	19·3 (18·7–20·0)	18·4 (18·1–18·8)
Bermuda	14 - 24	14 - 25	18·5 (18·0–18·9)	19·4 (18·8–20·1)	18·8 (18·4–19·1)
Cuba	14 - 24	14 - 26	18·5 (18·0–18·9)	19·5 (18·8–20·1)	18·8 (18·4–19·2)
Dominica	14 - 24	14 - 25	18·4 (17·9–18·8)	19·3 (18·6–20·0)	18·6 (18·2–19·0)
Dominican Republic	14 - 24	14 - 25	18·4 (17·9–18·8)	19·4 (18·8–20·0)	18·8 (18·4–19·1)
Grenada	14 - 24	14 - 25	18·4 (17·9–18·8)	19·4 (18·7–20·0)	18·6 (18·2–19·0)
Guyana	14 - 24	14 - 25	18·3 (17·9–18·7)	19·3 (18·7–20·0)	18·4 (18·0–18·8)
Haiti	14 - 24	14 - 25	18·4 (18·0–18·8)	19·4 (18·7–20·0)	18·6 (18·2–19·0)
Jamaica	14 - 24	14 - 25	18·4 (17·9–18·8)	19·3 (18·8–20·0)	18·6 (18·2–18·9)
Puerto Rico	14 - 24	14 - 25	18·3 (17·9–18·8)	19·4 (18·8–20·0)	18·7 (18·4–19·1)
Saint Kitts and Nevis	12 - 20	14 - 25	16·2 (15·8–16·6)	19·4 (18·7–20·1)	16·8 (16·4–17·2)
Saint Lucia	14 - 24	14 - 25	18·4 (17·9–18·8)	19·3 (18·7–19·9)	18·5 (18·2–18·9)
Saint Vincent and the Grenadines	14 - 24	14 - 25	18·3 (17·9–18·7)	19·3 (18·7–20·0)	18·5 (18·1–18·8)
Suriname	14 - 24	14 - 25	18·3 (17·9–18·8)	19·4 (18·7–20·1)	18·5 (18·2–18·9)
Trinidad and Tobago	14 - 24	14 - 25	18·4 (17·9–18·8)	19·4 (18·7–20·0)	18·6 (18·2–18·9)
United States Virgin Islands	14 - 24	15 - 25	19·0 (18·5–19·4)	19·5 (18·8–20·1)	19·2 (18·8–19·5)
Colombia	12 - 22	13 - 27	17·0 (16·6–17·4)	19·6 (18·9–20·3)	18·0 (17·7–18·5)
Costa Rica	12 - 21	13 - 26	16·5 (16·1–16·9)	18·9 (18·2–19·7)	17·3 (16·9–17·6)
El Salvador	12 - 22	13 - 28	16·9 (16·5–17·4)	19·5 (18·6–20·3)	17·3 (16·9–17·8)
Guatemala	13 - 21	14 - 26	16·8 (16·4–17·2)	19·2 (18·3–20·0)	17·1 (16·7–17·5)
Honduras	12 - 21	14 - 27	16·9 (16·5–17·3)	19·3 (18·5–20·1)	17·2 (16·8–17·6)
Mexico	12 - 22	13 - 26	16·9 (16·6–17·3)	18·9 (18·4–19·4)	17·5 (17·2–17·7)
Nicaragua	12 - 22	14 - 27	17·0 (16·6–17·4)	19·4 (18·6–20·2)	17·5 (17·1–17·9)
Panama	12 - 22	13 - 26	17·1 (16·7–17·5)	19·0 (18·1–19·8)	17·6 (17·2–17·9)
Venezuela (Bolivarian Republic of)	12 - 22	14 - 26	17·1 (16·7–17·5)	19·5 (18·7–20·2)	17·9 (17·5–18·3)
Brazil	12 - 23	13 - 24	17·4 (17·1–17·8)	17·6 (17·2–18·0)	17·5 (17·2–17·7)
Paraguay	13 - 23	13 - 23	17·3 (16·9–17·8)	17·4 (16·8–18·1)	17·4 (17·0–17·7)

**North Africa and Middle East**	**13 - 24**	**15 - 33**	**18·1 (18·0–18·3)**	**21·4 (21·1–21·8)**	**18·5 (18·4–18·7)**
Afghanistan	13 - 24	15 - 33	18·2 (17·7–18·7)	22·1 (21·0–23·1)	18·7 (18·2–19·1)
Algeria	13 - 24	15 - 33	18·4 (17·9–18·8)	22·6 (21·6–23·7)	18·6 (18·1–19·0)
Bahrain	13 - 25	15 - 32	18·6 (18·1–19·1)	23·2 (22·2–24·2)	19·0 (18·5–19·5)
Egypt	13 - 24	15 - 33	17·9 (17·6–18·3)	22·4 (21·5–23·5)	18·0 (17·7–18·3)
Iran (Islamic Republic of)	13 - 24	15 - 33	18·5 (18·1–19·0)	23·0 (22·1–23·9)	19·2 (18·8–19·6)
Iraq	13 - 24	15 - 33	18·3 (17·9–18·8)	23·2 (22·3–24·2)	18·6 (18·2–19·1)
Jordan	13 - 24	16 - 34	18·4 (18·0–18·8)	24·1 (23·1–25·0)	19·2 (18·9–19·6)
Kuwait	13 - 24	15 - 31	18·4 (18·0–18·9)	22·5 (21·6–23·4)	18·7 (18·3–19·2)
Lebanon	13 - 24	15 - 32	18·4 (18·0–18·9)	23·2 (22·4–24·1)	20·2 (19·8–20·7)
Libya	14 - 25	15 - 33	19·2 (18·8–19·6)	22·3 (21·2–23·4)	19·3 (18·9–19·7)
Morocco	13 - 24	15 - 33	18·4 (18·0–18·9)	22·6 (21·6–23·7)	18·6 (18·2–19·0)
Oman	13 - 24	15 - 31	18·4 (17·9–18·8)	22·3 (21·3–23·4)	18·5 (18·0–18·9)
Palestine	14 - 25	16 - 35	18·9 (18·6–19·3)	24·4 (23·4–25·4)	19·3 (19·0–19·7)
Qatar	13 - 24	15 - 31	18·3 (17·8–18·7)	22·7 (21·8–23·7)	18·4 (18·0–18·8)
Saudi Arabia	13 - 24	15 - 33	18·4 (17·9–18·8)	22·5 (21·5–23·6)	18·5 (18·1–19·0)
Sudan	13 - 24	15 - 31	18·2 (17·8–18·7)	22·2 (21·2–23·3)	18·5 (18·1–19·0)
Syrian Arab Republic	13 - 25	15 - 32	18·5 (18·0–18·9)	22·9 (21·8–23·9)	19·1 (18·6–19·5)
Tunisia	13 - 24	15 - 32	18·4 (18·0–18·9)	23·1 (22·0–24·1)	18·6 (18·2–19·1)
Turkey	13 - 22	14 - 27	17·3 (17·0–17·7)	20·0 (19·4–20·6)	18·1 (17·8–18·4)
United Arab Emirates	13 - 24	15 - 31	18·6 (18·1–19·2)	23·0 (22·0–24·0)	18·9 (18·4–19·4)
Yemen	13 - 24	15 - 31	18·3 (17·9–18·8)	22·5 (21·7–23·4)	19·2 (18·8–19·7)
**South Asia**	**14 - 27**	**14 - 36**	**20·2 (19·8–20·5)**	**22·4 (21·5–23·4)**	**20·4 (20·1–20·7)**
Bangladesh	13 - 25	14 - 40	18·3 (17·9–18·7)	26·3 (24·8–28·0)	18·6 (18·2–18·9)
Bhutan	14 - 27	14 - 35	19·9 (19·3–20·4)	22·2 (21·0–23·5)	20·4 (19·8–20·9)
India	14 - 29	14 - 35	20·6 (20·1–21·1)	22·1 (20·9–23·4)	20·7 (20·3–21·2)
Nepal	14 - 29	14 - 36	19·9 (19·4–20·4)	23·5 (22·5–24·7)	20·9 (20·5–21·5)

Pakistan	14 - 29	14 - 35	20·0 (19·5–20·5)	22·1 (20·8–23·3)	20·3 (19·8–20·8)
**Southeast Asia, East Asia, and Oceania**	**14 - 25**	**14 - 30**	**19·9 (19·6–20·2)**	**23·7 (23·1–24·4)**	**20·1 (19·9–20·4)**
China	15 - 27	16 - 35	20·5 (20·1–20·9)	24·9 (23·8–26·0)	20·7 (20·4–21·2)
Democratic People's Republic of Korea	15 - 28	16 - 35	20·6 (20·1–21·0)	24·7 (23·6–25·9)	20·9 (20·4–21·4)
Taiwan (Province of China)	15 - 28	16 - 32	20·6 (20·1–21·1)	23·8 (22·9–24·7)	21·0 (20·5–21·5)
American Samoa	14 - 27	14 - 31	19·5 (19·0–20·0)	21·1 (20·2–22·1)	20·1 (19·6–20·5)
Cook Islands	13 - 26	14 - 26	18·9 (18·5–19·4)	19·4 (18·7–20·1)	19·1 (18·8–19·5)
Fiji	14 - 26	15 - 27	19·5 (19·1–20·0)	20·3 (19·6–21·0)	19·7 (19·3–20·1)
Guam	13 - 25	14 - 26	18·8 (18·4–19·3)	19·5 (18·8–20·2)	19·1 (18·7–19·5)
Kiribati	13 - 24	14 - 27	17·7 (17·4–18·1)	19·9 (19·3–20·6)	18·5 (18·2–18·9)
Marshall Islands	13 - 25	14 - 26	18·6 (18·2–19·1)	19·2 (18·5–20·0)	18·8 (18·4–19·1)
Micronesia (Federated States of)	13 - 25	14 - 26	18·7 (18·2–19·1)	19·1 (18·4–19·8)	18·8 (18·5–19·2)
Nauru	12 - 22	12 - 21	16·8 (16·5–17·2)	16·2 (15·7–16·7)	16·5 (16·2–16·8)
Niue	13 - 25	14 - 26	18·9 (18·4–19·3)	19·3 (18·6–20·0)	19·0 (18·6–19·4)
Northern Mariana Islands	13 - 26	14 - 28	19·0 (18·5–19·5)	19·7 (18·9–20·7)	19·2 (18·8–19·7)
Palau	13 - 26	14 - 27	19·0 (18·6–19·5)	19·7 (19·0–20·5)	19·2 (18·8–19·6)
Papua New Guinea	13 - 25	14 - 26	18·6 (18·1–19·0)	19·0 (18·3–19·7)	18·7 (18·3–19·1)
Samoa	13 - 25	14 - 26	18·8 (18·4–19·3)	19·2 (18·6–19·9)	18·9 (18·6–19·3)
Solomon Islands	13 - 25	14 - 26	18·6 (18·2–19·1)	18·9 (18·2–19·6)	18·7 (18·4–19·1)
Tokelau	13 - 25	14 - 26	18·8 (18·4–19·2)	19·3 (18·5–20·0)	18·9 (18·5–19·3)
Tonga	13 - 25	15 - 29	18·2 (17·8–18·6)	21·0 (20·2–21·7)	18·9 (18·6–19·3)
Tuvalu	13 - 25	14 - 26	18·7 (18·2–19·1)	19·1 (18·5–19·8)	18·8 (18·4–19·2)
Vanuatu	14 - 27	14 - 25	20·0 (19·5–20·5)	18·3 (17·6–19·1)	19·8 (19·4–20·2)
Cambodia	14 - 26	14 - 34	19·4 (19·1–19·9)	22·2 (21·1–23·3)	19·7 (19·4–20·1)
Indonesia	13 - 24	14 - 30	18·3 (17·9–18·7)	20·8 (19·9–21·6)	18·5 (18·1–18·8)
Lao People's Democratic Republic	14 - 24	15 - 31	18·6 (18·1–19·0)	22·0 (21·1–23·0)	18·9 (18·5–19·4)
Malaysia	14 - 24	15 - 31	18·4 (18·0–18·7)	21·1 (20·0–22·1)	18·5 (18·1–18·8)

Maldives	14 - 24	15 - 33	18·5 (18·1–19·0)	22·3 (21·3–23·2)	18·8 (18·3–19·2)
Mauritius	14 - 25	14 - 32	18·6 (18·2–19·0)	22·2 (21·1–23·2)	18·9 (18·5–19·3)
Myanmar	14 - 25	14 - 34	18·5 (18·1–19·0)	23·4 (22·4–24·4)	19·2 (18·7–19·6)
Philippines	13 - 24	15 - 35	17·9 (17·6–18·3)	22·9 (21·9–23·8)	18·5 (18·2–18·9)
Seychelles	14 - 25	15 - 33	19·2 (18·8–19·7)	21·9 (21·0–23·0)	19·5 (19·1–19·9)
Sri Lanka	14 - 25	15 - 31	18·7 (18·2–19·1)	21·7 (20·7–22·7)	18·8 (18·4–19·2)
Thailand	13 - 24	14 - 31	18·2 (17·9–18·5)	21·3 (20·5–22·2)	18·4 (18·1–18·7)
Timor-Leste	14 - 24	14 - 33	18·4 (17·9–18·8)	22·1 (21·2–23·0)	18·6 (18·1–19·0)
Viet Nam	14 - 26	15 - 34	19·5 (19·2–19·9)	23·1 (22·1–24·0)	19·7 (19·4–20·1)
**Sub-Saharan Africa**	**14 - 27**	**15 - 31**	**20·0 (19·9–20·1)**	**21·2 (20·9–21·4)**	**20·2 (20·1–20·3)**
Angola	14 - 27	15 - 30	20·1 (19·6–20·5)	21·4 (20·5–22·3)	20·3 (19·8–20·7)
Central African Republic	14 - 28	15 - 30	20·0 (19·5–20·5)	21·1 (20·2–22·1)	20·1 (19·6–20·5)
Congo	14 - 27	15 - 30	20·1 (19·6–20·6)	21·4 (20·6–22·4)	20·2 (19·7–20·7)
Democratic Republic of the Congo	14 - 28	15 - 30	19·9 (19·5–20·4)	21·3 (20·4–22·2)	20·0 (19·6–20·5)
Equatorial Guinea	14 - 26	15 - 29	19·6 (19·1–20·1)	20·9 (20·0–21·9)	19·6 (19·2–20·2)
Gabon	14 - 28	15 - 29	19·8 (19·3–20·3)	20·6 (19·8–21·6)	19·9 (19·5–20·4)
Burundi	14 - 29	15 - 30	20·5 (20·1–21·0)	22·1 (21·3–23·0)	20·9 (20·4–21·3)
Comoros	13 - 25	13 - 29	18·5 (18·1–18·9)	20·2 (19·5–21·0)	18·7 (18·3–19·1)
Djibouti	14 - 28	15 - 30	20·5 (20·0–21·0)	21·8 (20·9–22·8)	20·6 (20·2–21·1)
Eritrea	14 - 28	15 - 30	19·9 (19·4–20·5)	21·1 (20·1–22·1)	20·0 (19·5–20·5)
Ethiopia	14 - 27	15 - 31	20·4 (19·9–20·9)	21·6 (20·7–22·6)	20·5 (20·1–21·0)
Kenya	15 - 28	15 - 31	20·7 (20·2–21·1)	21·5 (20·5–22·5)	20·7 (20·3–21·1)
Madagascar	14 - 27	15 - 31	20·0 (19·5–20·5)	21·9 (20·9–22·9)	20·2 (19·7–20·7)
Malawi	14 - 30	15 - 34	21·4 (20·9–21·9)	24·3 (23·3–25·2)	21·6 (21·2–22·1)
Mozambique	14 - 30	15 - 30	21·5 (21·0–22·0)	21·7 (20·9–22·5)	21·5 (21·1–22·0)
Rwanda	14 - 28	14 - 32	20·5 (20·0–20·9)	22·8 (21·9–23·8)	20·9 (20·5–21·4)
Somalia	14 - 27	15 - 31	20·2 (19·7–20·8)	21·9 (21·0–22·9)	20·4 (19·9–20·9)

South Sudan	14 - 29	14 - 31	20·5 (20·0–21·0)	22·2 (21·2–23·3)	20·7 (20·2–21·1)
Uganda	14 - 28	15 - 31	20·4 (20·0–20·9)	22·0 (21·2–23·0)	20·7 (20·3–21·1)
United Republic of Tanzania	14 - 27	15 - 31	20·4 (19·9–20·9)	21·9 (20·9–22·9)	20·6 (20·2–21·1)
Zambia	14 - 27	15 - 31	20·3 (19·9–20·8)	21·3 (20·3–22·3)	20·5 (20·0–20·9)
Botswana	16 - 29	17 - 40	21·3 (20·8–21·8)	26·8 (25·6–28·0)	22·2 (21·7–22·6)
Eswatini	14 - 26	15 - 30	19·6 (19·1–20·1)	21·5 (20·5–22·6)	19·8 (19·3–20·2)
Lesotho	14 - 26	15 - 31	19·5 (19·0–19·9)	21·7 (20·5–23·0)	19·5 (19·0–20·0)
Namibia	14 - 27	15 - 33	19·8 (19·3–20·2)	22·5 (21·5–23·4)	20·4 (20·0–20·9)
South Africa	14 - 25	13 - 25	18·8 (18·4–19·1)	18·5 (18·0–19·0)	18·7 (18·4–19·0)
Zimbabwe	14 - 26	15 - 31	19·7 (19·2–20·1)	21·8 (20·7–22·9)	19·8 (19·4–20·2)
Benin	15 - 30	15 - 34	21·4 (20·9–22·0)	23·5 (22·2–24·7)	21·7 (21·2–22·2)
Burkina Faso	15 - 27	15 - 30	20·1 (19·6–20·7)	21·5 (20·5–22·5)	20·3 (19·8–20·8)
Cabo Verde	14 - 25	15 - 31	19·1 (18·6–19·5)	22·7 (21·7–23·7)	19·8 (19·3–20·3)
Cameroon	14 - 27	15 - 30	20·2 (19·7–20·7)	21·7 (20·8–22·8)	20·3 (19·9–20·7)
Chad	15 - 27	15 - 31	20·2 (19·8–20·7)	21·6 (20·7–22·6)	20·4 (20·0–20·9)
Côte d'Ivoire	15 - 27	15 - 30	20·2 (19·7–20·8)	21·9 (20·9–22·8)	20·4 (19·9–20·9)
Gambia	15 - 26	16 - 31	19·6 (19·1–20·0)	22·2 (21·2–23·3)	19·7 (19·2–20·1)
Ghana	15 - 29	15 - 30	20·9 (20·3–21·4)	22·2 (21·2–23·2)	21·1 (20·6–21·6)
Guinea	15 - 27	15 - 31	20·3 (19·8–20·8)	21·9 (20·9–22·9)	20·4 (19·9–20·9)
Guinea-Bissau	15 - 27	15 - 31	20·3 (19·8–20·8)	22·0 (21·0–23·1)	20·5 (20·0–21·0)
Liberia	15 - 29	15 - 31	20·9 (20·4–21·4)	22·1 (21·2–23·1)	21·0 (20·6–21·5)
Mali	15 - 27	15 - 33	20·2 (19·7–20·7)	23·2 (22·1–24·5)	20·5 (20·0–20·9)
Mauritania	15 - 27	15 - 30	20·1 (19·6–20·6)	21·7 (20·9–22·6)	20·5 (20·0–20·9)
Niger	14 - 28	15 - 31	19·8 (19·4–20·3)	21·6 (20·6–22·7)	20·0 (19·6–20·4)
Nigeria	14 - 26	15 - 33	19·5 (19·0–20·0)	22·8 (21·7–24·0)	19·9 (19·5–20·4)
São Tomé and Príncipe	15 - 30	14 - 29	21·8 (21·3–22·3)	20·9 (20·2–21·7)	21·7 (21·3–22·1)
Senegal	15 - 27	15 - 31	20·3 (19·8–20·8)	22·0 (21·0–23·1)	20·4 (19·9–20·9)

Sierra Leone	14 - 28	15 - 33	20·1 (19·7–20·5)	22·3 (21·5–23·2)	20·6 (20·2–21·0)
Togo	15 - 31	15 - 32	22·5 (22·0–23·1)	22·8 (21·6–24·0)	22·5 (22·0–23·1)

Supplemental Figure S1. Absolute difference in mean age of initiation between males and females vs. absolute difference in smoking prevalence between males and females, 2019, by super region.

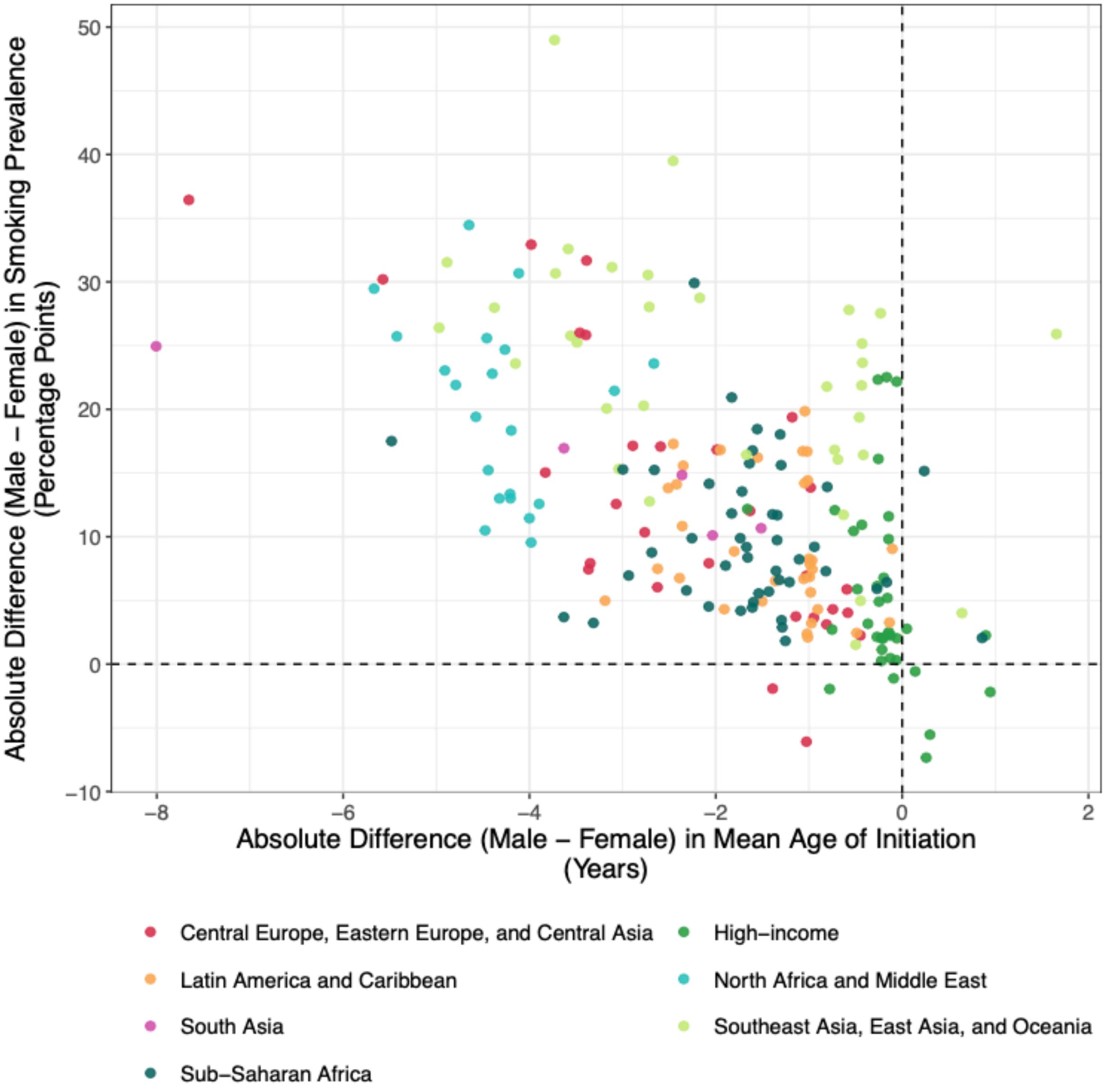

Supplemental Table S8. Smoking prevalence in 2019, by sex, for ages 15-19 and 20-24.

Location	Male Smoking Prevalence Ages 15-19	Female Smoking Prevalence Ages 15-19	Male Smoking Prevalence Ages 20-24	Female Smoking Prevalence Ages 20-24
**Global**	**12·8 (12·1–13·6)**	**4·70 (4·29–5·17)**	**27·8 (26·6–29·0)**	**5·20 (4·76–5·73)**
**Central Europe, Eastern Europe, and Central Asia**	**18·5 (17·2–19·8)**	**13·5 (11·8–15·4)**	**36·9 (35·0–38·8)**	**15·9 (14·2–17·8)**
Armenia	16·6 (12·5–21·1)	1·30 (0·549–2·58)	58·1 (52·0–64·1)	2·35 (1·02–4·57)
Azerbaijan	16·9 (13·5–20·6)	1·52 (0·654–3·21)	38·6 (33·0–44·3)	3·14 (1·36–6·07)
Georgia	24·2 (20·2–28·6)	6·98 (4·38–10·6)	55·1 (48·3–62·5)	7·28 (4·04–12·1)
Kazakhstan	11·1 (8·44–14·5)	5·51 (3·04–9·08)	37·0 (30·8–43·6)	8·80 (4·74–14·5)
Kyrgyzstan	12·9 (9·82–17·0)	2·99 (1·38–5·57)	33·0 (26·3–40·2)	4·89 (2·36–9·16)
Mongolia	19·1 (15·0–23·9)	4·52 (2·38–8·06)	50·1 (41·6–58·3)	5·83 (2·77–10·6)
Tajikistan	4·89 (3·33–6·90)	0·785 (0·303–1·63)	12·9 (9·48–16·6)	1·22 (0·487–2·63)
Turkmenistan	10·2 (6·89–14·2)	0·778 (0·297–1·65)	22·9 (16·7–29·8)	2·52 (1·05–4·83)
Uzbekistan	1·65 (1·03–2·49)	0·504 (0·192–1·09)	15·6 (11·8–20·3)	2·09 (0·846–4·23)
Albania	23·7 (19·0–28·9)	9·09 (5·32–14·1)	62·3 (57·0–67·5)	15·4 (8·05–26·3)
Bosnia and Herzegovina	23·4 (18·2–29·0)	19·1 (11·8–28·4)	38·3 (31·1–45·8)	29·0 (18·1–42·9)
Bulgaria	26·2 (21·2–31·9)	36·3 (27·5–45·7)	49·7 (42·1–56·9)	43·2 (32·0–55·2)
Croatia	32·2 (27·5–36·9)	30·5 (22·7–38·9)	41·3 (35·0–49·0)	38·6 (29·1–48·2)
Czechia	27·4 (23·6–31·3)	26·6 (20·6–32·8)	43·0 (36·9–49·5)	32·0 (24·8–39·9)
Hungary	25·0 (19·4–31·1)	27·8 (18·5–38·8)	40·1 (32·7–48·1)	31·5 (22·0–42·9)
Montenegro	14·0 (11·3–17·4)	9·14 (5·91–13·2)	26·3 (21·6–30·9)	23·7 (17·4–30·8)
North Macedonia	21·2 (17·4–24·9)	13·5 (9·63–18·3)	51·6 (44·1–59·3)	32·7 (23·6–43·5)
Poland	27·6 (23·7–31·8)	25·1 (20·1–31·1)	29·3 (24·9–34·2)	24·6 (18·2–32·2)
Romania	24·9 (21·1–29·4)	20·4 (15·2–26·4)	44·0 (38·4–49·8)	24·2 (17·4–31·0)
Serbia	25·7 (22·3–29·7)	31·9 (26·2–37·5)	29·2 (23·6–35·6)	35·2 (24·8–46·3)
Slovakia	31·7 (26·3–36·8)	32·9 (25·7–40·6)	33·0 (26·2–39·6)	24·3 (16·3–33·3)

Slovenia	26·2 (22·1–30·8)	23·8 (18·4–29·7)	38·7 (32·3–45·6)	32·7 (23·9–42·5)
Belarus	22·9 (18·0–28·2)	17·8 (11·9–25·4)	49·3 (40·6–57·5)	33·9 (23·1–46·9)
Estonia	21·8 (18·1–26·0)	18·7 (13·2–25·7)	35·1 (30·2–40·4)	22·6 (16·8–29·5)
Latvia	34·0 (29·4–38·9)	29·9 (22·4–38·7)	46·0 (40·3–52·1)	24·8 (17·9–32·8)
Lithuania	30·8 (27·4–34·7)	27·8 (23·4–32·3)	37·7 (31·1–44·5)	29·2 (21·5–38·4)
Republic of Moldova	21·2 (16·8–26·0)	6·50 (3·37–11·1)	46·8 (40·2–54·0)	10·5 (5·81–16·7)
Russian Federation	19·3 (15·9–22·7)	14·2 (9·65–19·9)	44·4 (39·1–49·7)	15·2 (10·5–20·4)
Ukraine	26·6 (21·6–31·9)	15·1 (9·92–21·6)	43·4 (37·3–49·6)	21·0 (13·9–29·2)
**High-income**	**17·3 (16·1–18·5)**	**13·7 (12·3–15·3)**	**30·7 (29·1–32·5)**	**20·6 (18·5–22·7)**
Australia	8·67 (6·27–11·4)	8·12 (4·64–12·9)	22·0 (16·6–28·4)	20·3 (12·1–30·5)
New Zealand	17·0 (14·0–20·3)	12·8 (9·10–17·7)	24·7 (21·1–28·5)	19·0 (14·7–24·1)
Brunei Darussalam	21·9 (16·4–28·0)	7·47 (3·82–12·8)	33·9 (24·9–44·5)	4·20 (1·56–8·86)
Japan	12·6 (8·24–18·2)	3·56 (1·38–7·35)	32·6 (25·7–39·8)	9·53 (5·28–15·5)
Republic of Korea	10·9 (8·05–14·6)	4·41 (2·12–8·17)	44·5 (35·7–53·7)	10·1 (4·98–17·6)
Singapore	10·7 (7·59–14·3)	7·08 (3·44–13·2)	24·2 (18·0–31·5)	7·78 (3·81–13·9)
Canada	10·1 (7·54–13·3)	9·53 (5·48–14·9)	23·5 (17·4–29·7)	20·1 (11·6–31·7)
Greenland	47·5 (38·4–56·2)	57·1 (41·2–71·9)	50·7 (39·1–61·5)	41·7 (22·8–63·4)
United States of America	14·0 (11·3–16·8)	10·4 (7·14–14·2)	22·9 (19·5–26·8)	16·1 (11·5–21·7)
Argentina	27·9 (22·9–33·6)	12·4 (6·89–20·1)	31·7 (25·3–38·2)	22·9 (14·6–33·2)
Chile	6·70 (4·63–9·42)	31·7 (19·4–45·5)	65·1 (55·4–74·2)	46·0 (31·6–60·9)
Uruguay	19·0 (14·9–24·0)	20·4 (13·2–29·0)	36·1 (27·3–45·0)	29·1 (16·0–44·9)
Andorra	23·7 (16·3–32·1)	23·2 (10·3–41·6)	35·2 (25·3–45·4)	32·0 (15·9–53·5)
Austria	27·8 (21·3–35·2)	22·5 (14·4–32·4)	45·2 (37·3–53·1)	29·4 (20·8–39·8)
Belgium	19·8 (16·2–24·2)	15·9 (11·2–21·7)	25·9 (21·4–30·6)	25·2 (18·3–33·1)
Cyprus	30·8 (24·7–37·5)	11·4 (6·38–19·5)	49·1 (40·0–57·6)	24·1 (14·4–36·9)
Denmark	16·8 (13·3–20·5)	22·0 (15·8–30·0)	24·9 (20·1–30·3)	21·4 (14·4–29·3)
Finland	21·6 (17·3–26·8)	22·0 (14·5–31·9)	25·3 (21·0–30·0)	24·3 (18·3–31·4)

France	31·9 (27·2–37·3)	30·9 (23·7–39·2)	44·6 (37·8–51·0)	39·0 (29·4–48·8)
Germany	22·3 (18·2–26·6)	18·5 (12·4–25·3)	35·8 (30·6–41·1)	27·9 (21·1–35·4)
Greece	22·9 (18·5–28·0)	18·1 (11·4–27·0)	50·0 (43·2–57·0)	30·2 (21·0–41·0)
Iceland	12·6 (9·67–16·0)	10·8 (6·88–15·8)	16·1 (12·2–20·7)	17·0 (9·82–26·5)
Ireland	15·2 (11·4–19·8)	17·1 (10·6–24·6)	26·7 (20·8–33·2)	39·8 (28·8–51·6)
Israel	20·1 (15·7–25·0)	9·55 (5·00–16·0)	28·8 (22·9–35·2)	16·0 (9·22–24·9)
Italy	22·0 (18·7–25·6)	17·0 (13·6–21·5)	36·1 (30·5–42·0)	20·4 (14·8–27·5)
Luxembourg	20·8 (16·7–25·9)	21·0 (15·1–27·7)	34·5 (27·5–41·8)	44·8 (35·3–54·5)
Malta	15·2 (11·7–19·0)	17·8 (12·6–24·0)	29·5 (22·7–37·0)	29·7 (19·0–43·0)
Monaco	22·2 (14·9–31·0)	21·2 (9·52–38·8)	32·6 (23·2–42·4)	29·2 (13·8–50·3)
Netherlands	20·0 (16·0–24·5)	14·5 (9·88–20·0)	29·5 (24·5–35·4)	30·4 (22·6–39·3)
Norway	13·2 (10·3–16·9)	10·2 (6·52–15·1)	19·7 (14·8–25·1)	17·6 (10·6–27·2)
Portugal	27·5 (22·8–32·2)	29·5 (22·2–38·1)	42·6 (35·6–49·1)	28·6 (20·6–38·3)
San Marino	17·7 (13·5–22·2)	13·8 (9·29–19·9)	24·7 (18·7–31·9)	23·8 (13·6–37·6)
Spain	19·5 (15·6–23·7)	21·7 (16·1–29·1)	37·0 (31·2–43·0)	30·6 (22·9–39·8)
Sweden	13·6 (9·72–17·9)	18·0 (10·6–27·0)	14·9 (11·0–19·6)	14·8 (8·23–23·4)
Switzerland	17·4 (14·0–21·2)	12·0 (8·55–16·4)	35·8 (28·6–43·6)	27·8 (18·1–40·2)
United Kingdom	18·9 (15·3–23·1)	18·3 (12·7–25·1)	25·3 (21·1–30·0)	20·6 (14·3–27·9)
**Latin America and Caribbean**	**12·7 (11·7–13·9)**	**7·19 (5·88–8·79)**	**19·5 (18·1–21·1)**	**7·12 (5·74–8·77)**
Bolivia (Plurinational State of)	22·5 (17·8–28·6)	13·0 (7·78–19·9)	13·2 (9·67–17·6)	5·02 (2·24–9·67)
Ecuador	9·52 (6·93–12·9)	4·69 (2·58–7·74)	33·6 (29·0–38·3)	5·55 (2·94–9·06)
Peru	15·2 (11·7–19·1)	5·46 (2·97–9·00)	9·50 (6·60–13·2)	6·26 (3·25–10·9)
Antigua and Barbuda	8·43 (5·99–11·6)	9·38 (5·82–14·4)	9·09 (5·88–13·1)	3·55 (1·37–7·31)
Bahamas	15·9 (12·4–20·0)	6·63 (3·63–10·8)	9·50 (6·70–13·5)	2·18 (0·837–4·56)
Barbados	12·8 (9·06–17·1)	11·7 (6·76–19·1)	13·5 (8·91–19·3)	4·58 (2·05–9·05)
Belize	19·6 (14·8–24·7)	8·92 (4·82–14·7)	20·5 (14·3–27·9)	2·39 (0·923–5·05)
Bermuda	12·6 (8·70–17·3)	6·02 (2·59–11·1)	17·5 (12·3–24·0)	7·64 (3·69–13·9)

Cuba	12·2<br>(9·32–15·7)	10·5<br>(6·61–15·0)	24·8<br>(18·5–31·5)	13·0<br>(7·08–21·4)
Dominica	14·7<br>(10·2–20·3)	9·34<br>(4·44–17·1)	11·0<br>(7·28–15·8)	7·85<br>(3·31–15·2)
Dominican Republic	4·92<br>(3·51–6·66)	4·73<br>(2·63–7·93)	7·05<br>(4·91–9·84)	3·06<br>(1·32–5·98)
Grenada	15·8<br>(12·1–20·1)	9·54<br>(5·39–14·9)	13·4<br>(8·93–19·1)	4·03<br>(1·55–8·31)
Guyana	14·5<br>(11·1–18·9)	7·46<br>(4·05–12·7)	28·0<br>(22·3–34·2)	2·56<br>(1·03–5·08)
Haiti	5·67<br>(3·81–8·17)	3·74<br>(1·70–6·98)	6·05<br>(4·21–8·54)	1·47<br>(0·618–3·10)
Jamaica	17·9<br>(14·8–21·8)	14·4<br>(10·4–19·1)	12·7<br>(8·96–17·1)	5·13<br>(2·29–9·39)
Puerto Rico	8·98<br>(6·28–12·2)	4·01<br>(1·83–7·72)	16·6<br>(12·6–21·0)	8·09<br>(4·82–12·9)
Saint Kitts and Nevis	5·81<br>(3·73–8·63)	2·58<br>(0·972–5·68)	9·48<br>(6·17–13·7)	2·73<br>(0·989–5·97)
Saint Lucia	13·6<br>(10·3–17·4)	11·3<br>(6·75–17·4)	16·9<br>(10·9–23·9)	4·50<br>(1·69–9·23)
Saint Vincent and the Grenadines	25·4<br>(20·1–31·3)	11·8<br>(6·47–19·4)	17·9<br>(12·7–23·8)	2·67<br>(1·08–5·83)
Suriname	25·1<br>(20·1–30·1)	10·9<br>(6·39–16·8)	32·8<br>(24·7–41·7)	7·07<br>(2·98–13·9)
Trinidad and Tobago	24·3<br>(20·1–29·3)	12·6<br>(8·08–18·0)	28·7<br>(21·1–36·8)	6·96<br>(3·03–13·7)
United States Virgin Islands	2·74<br>(1·75–3·97)	0·574<br>(0·209–1·28)	7·01<br>(4·63–10·1)	4·11<br>(1·69–8·02)
Colombia	14·6<br>(11·1–18·8)	8·15<br>(4·75–13·0)	18·5<br>(14·1–23·3)	9·98<br>(5·98–15·4)
Costa Rica	12·5<br>(9·35–16·1)	9·80<br>(5·61–15·7)	17·7<br>(13·4–22·8)	7·21<br>(3·75–12·9)
El Salvador	24·0<br>(19·4–29·0)	9·48<br>(5·63–14·4)	15·3<br>(10·9–20·9)	2·43<br>(0·908–5·04)
Guatemala	20·2<br>(16·4–25·0)	11·6<br>(7·10–17·7)	25·5<br>(20·0–31·5)	2·83<br>(1·10–5·85)
Honduras	18·5<br>(14·7–22·9)	9·51<br>(5·80–14·4)	29·6<br>(22·5–36·8)	3·45<br>(1·40–7·02)
Mexico	18·7<br>(15·1–22·5)	9·56<br>(5·70–14·9)	35·5<br>(31·0–40·2)	10·7<br>(6·90–15·8)
Nicaragua	26·2<br>(20·7–32·0)	12·2<br>(7·05–18·8)	18·3<br>(12·5–25·5)	4·17<br>(1·46–9·14)
Panama	11·4<br>(8·33–14·8)	6·62<br>(3·63–11·1)	10·0<br>(6·70–14·4)	6·16<br>(2·60–12·2)
Venezuela (Bolivarian Republic of)	12·4<br>(8·81–16·9)	8·92<br>(4·14–16·7)	24·7<br>(17·6–33·3)	5·63<br>(2·22–11·4)
Brazil	6·22<br>(4·73–7·85)	4·47<br>(2·54–7·26)	10·9<br>(8·56–13·6)	6·19<br>(3·61–9·58)
Paraguay	13·9<br>(10·2–18·4)	6·88<br>(3·78–11·9)	18·8<br>(13·5–24·8)	7·66<br>(3·46–14·2)
**North Africa and Middle East**	**19·5<br>(18·3–20·7)**	**7·15<br>(6·15–8·21)**	**30·5<br>(28·9–32·1)**	**4·01<br>(3·25–4·99)**
Afghanistan	11·8<br>(8·49–16·0)	1·99<br>(0·805–4·23)	18·9<br>(14·5–24·0)	3·11<br>(1·29–5·85)

Algeria	23·1 (18·6–27·9)	2·07 (0·841–3·99)	29·4 (23·0–36·3)	1·24 (0·459–2·77)
Bahrain	32·2 (26·5–38·3)	10·6 (6·69–15·8)	19·9 (13·7–28·4)	2·69 (0·996–5·91)
Egypt	16·2 (13·2–19·7)	2·77 (1·33–4·97)	39·2 (35·1–43·6)	0·545 (0·206–1·20)
Iran (Islamic Republic of)	11·9 (8·57–16·1)	4·90 (2·34–9·14)	16·4 (12·1–21·0)	2·64 (1·06–5·77)
Iraq	25·5 (20·8–30·7)	5·78 (3·18–10·1)	27·5 (21·5–34·0)	1·02 (0·367–2·24)
Jordan	32·9 (28·5–37·8)	14·9 (9·63–21·9)	56·5 (50·4–61·9)	15·0 (8·54–23·4)
Kuwait	35·8 (30·7–41·4)	13·9 (8·94–19·7)	40·5 (32·6–49·4)	2·95 (1·13–6·00)
Lebanon	35·2 (29·7–41·1)	13·3 (8·62–19·2)	35·3 (28·9–42·1)	13·4 (7·43–21·5)
Libya	16·1 (11·5–21·6)	2·80 (1·15–5·55)	31·1 (23·5–39·8)	1·47 (0·530–3·19)
Morocco	10·7 (7·97–13·8)	2·60 (1·28–4·64)	19·1 (14·6–24·1)	0·907 (0·339–1·99)
Oman	9·48 (6·96–12·6)	2·44 (1·03–4·77)	12·4 (8·62–17·3)	1·47 (0·585–3·19)
Palestine	20·0 (15·1–26·1)	4·45 (2·15–8·11)	38·3 (29·8–46·4)	1·94 (0·774–4·04)
Qatar	26·9 (20·9–33·8)	11·6 (6·29–18·4)	20·1 (15·3–26·0)	1·20 (0·425–2·67)
Saudi Arabia	11·9 (8·69–15·8)	3·70 (1·70–6·73)	28·0 (23·0–33·4)	1·60 (0·603–3·60)
Sudan	9·34 (6·54–12·9)	2·80 (1·25–5·38)	18·2 (13·7–23·7)	1·19 (0·434–2·78)
Syrian Arab Republic	22·0 (16·4–29·0)	8·65 (4·11–15·3)	40·8 (30·8–51·6)	4·37 (1·76–9·33)
Tunisia	25·4 (21·2–30·0)	3·71 (1·87–6·55)	49·3 (41·0–57·5)	1·74 (0·648–3·64)
Turkey	41·5 (37·3–46·6)	28·3 (22·8–34·4)	49·5 (44·5–54·5)	15·8 (10·6–22·3)
United Arab Emirates	15·7 (12·0–19·8)	5·32 (2·82–9·35)	18·6 (13·8–24·1)	2·72 (1·10–5·54)
Yemen	15·6 (11·8–19·9)	4·96 (2·61–8·52)	22·2 (16·5–29·2)	5·44 (2·34–10·4)
**South Asia**	**8·00 (6·43–10·1)**	**1·92 (1·04–3·33)**	**19·9 (17·0–23·1)**	**1·89 (1·07–3·06)**
Bangladesh	14·2 (10·9–18·3)	1·06 (0·425–2·05)	38·9 (32·7–45·5)	1·22 (0·444–2·55)
Bhutan	36·6 (29·4–44·3)	13·2 (7·91–19·8)	9·59 (6·56–13·8)	1·61 (0·662–3·47)
India	7·44 (5·44–9·95)	2·16 (0·986–4·01)	18·1 (14·4–22·1)	1·80 (0·788–3·35)
Nepal	12·6 (9·83–16·1)	2·31 (1·02–4·38)	28·3 (23·7–33·8)	3·72 (1·81–6·88)
Pakistan	6·82 (4·90–9·21)	1·16 (0·487–2·29)	18·0 (13·6–22·9)	2·60 (1·12–5·13)
**Southeast Asia, East Asia, and Oceania**	**18·3 (16·3–20·7)**	**2·85 (2·13–3·81)**	**45·3 (42·2–48·4)**	**2·54 (1·79–3·77)**

China	15·0 (11·8–18·9)	2·19 (1·14–3·95)	44·3 (39·2–49·4)	2·15 (0·959–4·11)
Democratic People's Republic of Korea	14·1 (10·4–18·8)	1·67 (0·625–3·38)	36·5 (28·5–44·6)	3·07 (1·19–6·28)
Taiwan (Province of China)	12·6 (9·15–16·9)	0·462 (0·172–1·06)	34·4 (27·6–41·3)	7·81 (3·87–14·0)
American Samoa	30·0 (21·6–39·4)	18·7 (9·28–32·4)	47·2 (36·0–57·8)	24·3 (11·2–43·5)
Cook Islands	33·0 (26·0–40·6)	45·6 (29·9–61·3)	39·1 (30·6–48·4)	23·0 (12·1–37·8)
Fiji	24·5 (20·0–29·6)	11·5 (7·40–17·2)	49·3 (38·2–59·9)	18·5 (8·40–32·7)
Guam	25·8 (21·9–30·0)	14·7 (10·3–19·7)	30·0 (24·2–36·0)	17·9 (11·6–25·5)
Kiribati	42·8 (36·1–49·9)	26·3 (17·7–36·5)	67·1 (60·4–73·5)	25·6 (15·8–37·7)
Marshall Islands	36·8 (31·1–42·8)	14·9 (9·67–21·2)	43·7 (33·9–53·4)	9·15 (3·69–17·6)
Micronesia (Federated States of)	59·0 (51·4–65·9)	37·6 (26·8–49·6)	68·9 (59·3–77·4)	39·3 (22·5–57·8)
Nauru	44·6 (36·3–52·7)	35·6 (23·0–50·1)	46·6 (37·9–55·0)	48·2 (33·1–63·5)
Niue	13·6 (9·52–18·5)	9·72 (5·06–17·3)	26·9 (20·7–34·1)	19·7 (11·0–32·0)
Northern Mariana Islands	31·5 (24·2–39·4)	21·9 (14·8–31·1)	44·8 (34·3–55·7)	19·0 (8·10–34·8)
Palau	46·7 (41·8–51·3)	34·0 (28·3–40·4)	32·4 (25·0–40·1)	10·9 (5·44–18·0)
Papua New Guinea	44·1 (37·4–50·9)	26·5 (19·0–34·7)	49·4 (41·2–58·3)	19·5 (10·5–30·9)
Samoa	21·2 (17·4–25·5)	7·73 (4·51–12·1)	33·0 (26·5–40·0)	12·7 (7·56–20·0)
Solomon Islands	35·8 (30·6–41·7)	15·1 (10·3–21·3)	59·2 (52·2–65·5)	23·2 (16·0–31·7)
Tokelau	30·3 (21·8–40·3)	15·7 (6·24–29·8)	43·5 (32·8–54·7)	17·9 (7·86–33·0)
Tonga	24·6 (19·5–30·5)	11·5 (6·50–18·2)	44·9 (37·0–52·9)	15·6 (9·32–23·5)
Tuvalu	31·7 (26·3–37·6)	14·0 (9·13–20·7)	49·4 (40·1–58·1)	23·0 (12·3–36·2)
Vanuatu	24·0 (19·3–29·1)	11·7 (7·07–17·4)	50·2 (41·4–58·6)	9·24 (4·28–16·1)
Cambodia	6·13 (4·25–8·43)	1·29 (0·532–2·58)	23·6 (19·2–28·9)	2·97 (1·32–5·80)
Indonesia	26·9 (22·8–31·4)	2·40 (1·18–4·30)	57·7 (52·9–62·2)	2·07 (0·931–4·12)
Lao People's Democratic Republic	21·9 (18·1–26·3)	4·54 (2·38–7·68)	35·7 (28·3–43·9)	2·36 (0·921–4·70)
Malaysia	20·6 (16·5–24·6)	3·73 (2·01–6·43)	45·8 (38·6–53·4)	2·35 (0·965–4·62)
Maldives	20·6 (16·2–25·6)	8·35 (4·61–13·9)	46·8 (38·3–55·4)	3·20 (1·30–6·34)
Mauritius	36·6 (31·1–42·0)	15·7 (10·2–22·7)	46·6 (39·3–54·1)	2·46 (0·902–5·14)

Myanmar	26·6<br>(22·1–31·4)	2·38<br>(1·11–4·39)	40·6<br>(34·5–47·2)	1·11<br>(0·398–2·37)
Philippines	22·8<br>(19·0–27·0)	7·71<br>(4·84–11·7)	44·0<br>(37·8–49·7)	5·65<br>(3·30–8·75)
Seychelles	29·6<br>(23·8–36·2)	15·0<br>(9·55–22·1)	45·3<br>(33·9–56·3)	4·57<br>(1·80–9·69)
Sri Lanka	6·15<br>(4·46–8·15)	0·818<br>(0·320–1·72)	27·3<br>(21·9–33·2)	1·01<br>(0·385–2·33)
Thailand	24·5<br>(20·6–28·8)	3·20<br>(1·63–5·74)	42·5<br>(36·7–47·8)	2·60<br>(1·15–5·05)
Timor-Leste	38·2<br>(32·4–44·6)	5·31<br>(2·86–8·79)	71·1<br>(64·9–76·9)	2·03<br>(0·823–4·28)
Viet Nam	12·2<br>(9·14–15·7)	0·970<br>(0·376–2·13)	40·7<br>(34·7–46·6)	1·24<br>(0·470–2·50)
**Sub-Saharan Africa**	**6·65<br>(6·25–7·11)**	**2·40<br>(2·02–2·83)**	**13·4<br>(12·5–14·2)**	**1·79<br>(1·46–2·18)**
Angola	3·54<br>(2·27–5·22)	1·07<br>(0·397–2·32)	13·0<br>(9·27–17·5)	1·30<br>(0·485–2·87)
Central African Republic	6·67<br>(4·44–9·59)	1·63<br>(0·571–3·60)	12·9<br>(8·41–18·2)	0·850<br>(0·325–1·81)
Congo	12·8<br>(8·80–17·3)	3·99<br>(1·72–7·59)	15·7<br>(10·9–21·3)	0·632<br>(0·239–1·38)
Democratic Republic of the Congo	4·51<br>(2·91–6·70)	0·469<br>(0·164–0·987)	17·1<br>(12·4–23·0)	0·650<br>(0·231–1·42)
Equatorial Guinea	23·1<br>(17·2–29·2)	8·39<br>(3·86–15·5)	22·9<br>(16·7–30·1)	1·03<br>(0·387–2·26)
Gabon	16·1<br>(12·3–20·6)	8·17<br>(4·35–14·0)	23·2<br>(17·6–29·7)	2·99<br>(1·13–6·28)
Burundi	3·02<br>(1·93–4·41)	0·910<br>(0·353–2·02)	9·64<br>(6·68–13·4)	1·37<br>(0·503–3·20)
Comoros	15·5<br>(11·9–19·6)	6·15<br>(3·22–10·6)	19·9<br>(14·6–26·2)	1·75<br>(0·639–3·67)
Djibouti	15·8<br>(11·6–20·3)	6·34<br>(3·30–11·3)	25·8<br>(18·7–33·7)	3·75<br>(1·54–8·04)
Eritrea	3·39<br>(2·17–5·02)	0·548<br>(0·183–1·21)	11·5<br>(7·70–16·5)	0·969<br>(0·383–2·12)
Ethiopia	2·07<br>(1·29–3·06)	0·382<br>(0·135–0·839)	6·71<br>(4·72–9·19)	1·12<br>(0·462–2·29)
Kenya	6·25<br>(4·28–8·93)	2·20<br>(1·01–4·11)	13·2<br>(9·79–17·1)	1·96<br>(0·827–3·82)
Madagascar	17·3<br>(13·4–22·2)	4·23<br>(2·07–7·66)	31·4<br>(24·4–39·3)	1·25<br>(0·472–2·75)
Malawi	5·89<br>(4·09–8·37)	2·58<br>(1·19–4·94)	12·9<br>(9·45–16·6)	0·994<br>(0·377–2·11)
Mozambique	6·15<br>(4·11–8·56)	2·54<br>(1·11–4·92)	15·3<br>(10·7–20·9)	5·22<br>(2·15–9·96)
Rwanda	3·11<br>(2·05–4·52)	0·553<br>(0·220–1·22)	10·6<br>(7·54–14·4)	1·03<br>(0·408–2·24)
Somalia	6·39<br>(4·19–9·28)	2·72<br>(1·06–5·74)	15·5<br>(10·3–22·7)	1·39<br>(0·497–3·04)
South Sudan	7·95<br>(5·43–11·0)	1·90<br>(0·768–3·87)	15·4<br>(10·2–21·8)	1·45<br>(0·518–3·17)
Uganda	7·53<br>(5·54–9·85)	5·00<br>(2·63–8·87)	8·16<br>(5·84–10·8)	1·39<br>(0·546–2·86)

United Republic of Tanzania	4·77<br>(3·26–6·87)	1·89<br>(0·793–3·89)	11·3<br>(8·23–15·1)	2·04<br>(0·854–4·32)
Zambia	15·7<br>(12·2–19·8)	9·14<br>(5·02–15·2)	17·0<br>(13·2–21·2)	4·73<br>(2·10–9·28)
Botswana	22·5<br>(17·6–28·2)	8·14<br>(4·66–12·9)	27·6<br>(21·9–33·9)	6·85<br>(3·69–11·3)
Eswatini	7·55<br>(5·19–10·6)	2·56<br>(1·13–4·89)	11·7<br>(8·47–15·8)	0·967<br>(0·366–2·01)
Lesotho	19·1<br>(14·5–25·0)	2·61<br>(1·10–5·27)	44·6<br>(37·7–51·1)	0·499<br>(0·191–1·11)
Namibia	14·8<br>(10·8–19·2)	11·7<br>(6·43–19·6)	19·5<br>(15·0–24·7)	4·77<br>(2·14–9·06)
South Africa	20·9<br>(16·8–25·6)	16·4<br>(9·40–25·1)	34·7<br>(29·8–39·4)	9·21<br>(4·65–15·4)
Zimbabwe	12·3<br>(9·05–16·5)	4·62<br>(2·27–8·36)	23·2<br>(18·8–28·4)	1·23<br>(0·475–2·51)
Benin	4·38<br>(2·93–6·21)	0·979<br>(0·400–2·08)	7·43<br>(5·02–10·3)	1·57<br>(0·611–3·06)
Burkina Faso	8·69<br>(5·89–12·5)	1·26<br>(0·445–2·76)	19·0<br>(14·1–24·6)	1·68<br>(0·651–3·66)
Cabo Verde	4·55<br>(3·02–6·72)	2·01<br>(0·798–4·19)	6·17<br>(4·01–8·98)	1·36<br>(0·516–2·98)
Cameroon	4·98<br>(3·44–7·03)	1·76<br>(0·860–3·19)	9·66<br>(6·71–13·4)	1·30<br>(0·503–2·81)
Chad	6·59<br>(4·45–9·46)	2·50<br>(1·03–5·19)	14·2<br>(9·95–19·4)	2·58<br>(1·08–5·31)
Côte d'Ivoire	16·2<br>(11·7–21·6)	4·46<br>(1·90–8·67)	23·0<br>(17·0–29·5)	2·62<br>(0·968–5·61)
Gambia	15·3<br>(11·6–19·4)	2·86<br>(1·37–5·20)	19·1<br>(14·2–24·7)	0·589<br>(0·216–1·35)
Ghana	5·09<br>(3·56–7·27)	1·70<br>(0·741–3·24)	3·01<br>(1·86–4·51)	0·738<br>(0·298–1·53)
Guinea	15·6<br>(11·0–21·5)	1·57<br>(0·605–3·39)	25·7<br>(18·1–34·5)	1·57<br>(0·583–3·37)
Guinea-Bissau	3·16<br>(2·03–4·76)	0·789<br>(0·283–1·73)	7·06<br>(4·39–10·5)	0·763<br>(0·285–1·73)
Liberia	3·73<br>(2·51–5·59)	3·78<br>(1·81–6·71)	6·29<br>(4·03–9·63)	2·26<br>(0·939–4·59)
Mali	13·3<br>(9·47–17·9)	1·22<br>(0·486–2·52)	20·2<br>(15·3–25·8)	0·836<br>(0·309–1·78)
Mauritania	17·7<br>(14·4–21·5)	10·7<br>(6·99–15·0)	37·3<br>(27·9–48·8)	8·51<br>(3·80–16·5)
Niger	12·2<br>(8·55–16·6)	2·35<br>(0·998–4·69)	16·3<br>(12·1–22·0)	1·97<br>(0·856–4·11)
Nigeria	1·98<br>(1·24–2·90)	0·500<br>(0·189–1·08)	5·91<br>(3·99–8·25)	0·476<br>(0·182–1·07)
São Tomé and Príncipe	1·10<br>(0·683–1·69)	0·478<br>(0·174–1·11)	4·45<br>(2·88–6·66)	0·657<br>(0·237–1·37)
Senegal	9·91<br>(7·27–13·1)	2·12<br>(0·950–4·04)	13·3<br>(10·1–17·2)	0·930<br>(0·338–1·95)
Sierra Leone	14·4<br>(11·2–18·4)	6·98<br>(3·70–11·8)	20·4<br>(15·0–27·0)	7·68<br>(3·42–14·7)
Togo	7·48<br>(5·26–10·4)	1·92<br>(0·807–3·54)	7·13<br>(4·79–9·89)	0·859<br>(0·292–1·86)
